# Supplementary material for: Differences between gridded population data impact measures of geographic access to healthcare in sub-Saharan Africa
Source: Commun Med (Lond). 2022 Sep 16;2:117. doi: 10.1038/s43856-022-00179-4 (PMC9481590; doi:10.1038/s43856-022-00179-4)
Supplement: Supplementary file 2 — Description of Additional Supplementary Files [file 43856_2022_179_MOESM2_ESM.pdf]

Description of Additional Supplementary Files

File Name: Supplementary Data 1

Description: Travel scenario as developed for the current study. The dataset lists a travel speed (km/h) for each unique road category and land cover class in the different countries in sub-Saharan Africa. A glossary for the column names is provided in the Read_me sheet of the dataset.

File Name: Supplementary Data 2

Description: Healthcare coverage statistics at administrative level 1 and 2 for sub-Saharan Africa using the six different gridded population datasets. Coverages have been calculated for 30, 60, 90, 120, 150, and 180 minutes travel time. The file comprises two distinct sheets: (1) Coverage_statistics_admin_1, (2) Coverage_statistics_admin_2. The first sheet presents the coverage statistics for administrative level 1 in sub-Saharan Africa. The second sheet presents all the coverage statistics at administrative level 2. A glossary for the column names is provided in the Read_me sheet of the dataset
