# Supplementary material for: Differences between gridded population data impact measures of geographic access to healthcare in sub-Saharan Africa
Source: Commun Med (Lond). 2022 Sep 16;2:117. doi: 10.1038/s43856-022-00179-4 (PMC9481590; doi:10.1038/s43856-022-00179-4)
Supplement: Supplementary file 4 — Supplementary Material [file 43856_2022_179_MOESM4_ESM.pdf]

Supplementary Table 1 – Country spatial projection system.

Country code	Country name	EPSG code	Projection
AGO	Angola	22033	Camacupa 1948 / UTM zone 33S
BDI	Burundi	32736	WGS 84 / UTM zone 36S
BEN	Benin	32631	WGS 84 / UTM zone 31N
BFA	Burkina Faso	32630	WGS 84 / UTM zone 30N
BWA	Botswana	20934	Arc 1950 / UTM zone 34S
CAF	Central African Republic	32635	WGS 84 / UTM zone 35N
CIV	Côte d'Ivoire	2043	Abidjan 1987 / UTM zone 29N
CMR	Cameroon	2215	Manoca 1962 / UTM zone 32N
COD	Democratic Republic of the Congo	7992	Malongo 1987 / UTM zone 33S
COG	Republic of Congo	7992	Malongo 1987 / UTM zone 33S
COM	Comoros	2999	Grand Comoros / UTM zone 38S
CPV	Cape Verde	4826	WGS 84 / Cape Verde National
DJI	Djibouti	32637	WGS 84 / UTM zone 37N
DZA	Algeria	30729	Nord Sahara 1959 / UTM zone 29N
EGY	Egypt	32637	WGS 84 / UTM zone 37N
ERI	Eritrea	26237	Massawa / UTM zone 37N
ESH	Western Sahara	26195	Merchich / Sahara Sud
ETH	Ethiopia	20138	Adindan / UTM zone 38N
GAB	Gabon	26692	M'poraloko / UTM zone 32S
GHA	Ghana	2136	Accra / Ghana National Grid
GIN	Guinea	3462	Dabola 1981 / UTM zone 29N
GMB	Gambia	6894	Gambia
GNB	Guinea-Bissau	2095	Bissau / UTM zone 28N
GNQ	Equatorial Guinea	6883	Bioko
KEN	Kenya	21097	Arc 1960 / UTM zone 37N
LBR	Liberia	4251	Liberia 1964
LBY	Libya	3177	LGD2006 / Libya TM
LSO	Lesotho	4222	Cape
MAR	Morocco	4261	Merchich
MDG	Madagascar	29739	Tananarive / UTM zone 39S
MLI	Mali	32630	WGS 84 / UTM zone 30N
MOZ	Mozambique	5629	Moznet / UTM zone 38S
MRT	Mauritania	3345	Mauritania 1999 / UTM zone 30N
MUS	Mauritius	3337	Le Pouce 1934 / Mauritius Grid
MWI	Malawi	20936	Arc 1950 / UTM zone 36S
NAM	Namibia	29333	Schwarzeck / UTM zone 33S
NER	Niger	2931	Beduaram / TM 13 NE
NGA	Nigeria	4263	Minna
RWA	Rwanda	32736	WGS 84 / UTM zone 36S
SDN	Sudan	20135	Adindan / UTM zone 35N
SEN	Senegal	31028	Yoff / UTM zone 28N
SLE	Sierra Leone	2162	Sierra Leone 1968 / UTM zone 29N
SOM	Somalia	20539	Afgooye / UTM zone 39N
SSD	South Sudan	20135	Adindan / UTM zone 35N
STP	São Tomé and Príncipe	4823	Sao Tome
SWZ	Swaziland	22291	Cape / Lo31
SYC	Seychelles	6915	South East Island 1943 / UTM zone 40N
TCD	Chad	32434	WGS 72BE / UTM zone 34N
TGO	Togo	25231	Lome / UTM zone 31N
TUN	Tunisia	22332	Carthage / UTM zone 32N
TZA	Tanzania	21037	Arc 1960 / UTM zone 37S
UGA	Uganda	21095	Arc 1960 / UTM zone 35N
ZAF	South Africa	22293	Cape / Lo33
ZMB	Zambia	20934	Arc 1950 / UTM zone 34S
ZWE	Zimbabwe	20936	Arc 1950 / UTM zone 36S

Supplementary Table 2 – Description of gridded population datasets as adapted from[19, 20, 22].

Gridded population dataset	Name dataset	Producer	Resolution	Year	Method	Covariates	Distribution	Citation
**GHS-POP**	GHS POP MT GLOBE R2019A	European Commission, Joint Research Centre (JRC)	250m (World Mollweide)	2015	Binary dasymetric. Population is proportionally redistributed to built-up areas from the Global Human Settlement Layer Built-Up Extent (GHS-BUILT), extracted from 30m Landsat imagery.	Built-up areas from the GHS-BUILT	Open access (Creative commons Attribution 4.0 International License)	13
**GPWv4**	UN WPP-Adjusted Population Count, v4.11	CIESN, Columbia University	30 arc-second (approximately 1km)	2020	Aerial weighting. Equal redistribution of census within administrative boundaries	Protected areas and water bodies	Open access (Creative commons Attribution 4.0 International License)	14
**LandScan**	Landscan Global Population Database	Oak Ridge National Laboratory	30 arc-second (approximately 1km)	2018	Multivariable dasymetric mapping with 4 covariates to estimate the 24h average ambient population	Roads, land cover, built structures, cities or urban areas infrastructure, environmental data, protected areas and water bodies.	Open access for academic research, humanitarian purposes, and for US government agencies or staff. Commercial license available for other entities.	17
**HRSL**	High Resolution Settlement Layer	Facebook Connectivity Lab and CIESN	1 arc-second (approximately 30m)	2015	Binary dasymetric. Population is redistributed to cells with built structures as extracted from 0.5m Maxar imagery (0.5m resolution).	Built structures	Open access	16
**WorldPop**	Constrained (top-down)	WorldPop, University of Southampton	3 arc-second (approximately 100m)	2020	Random Forest model with 24 covariates and binary dasymetric reallocation of population constrained to cells with settlements.	Roads, land cover, built structures, cities or urban areas, nighttime lights, infrastructure, environmental data, protected areas and water bodies. Constrained to built structures.	Open access	12
	Unconstrained (top-down)	WorldPop, University of Southampton	3 arc-second (approximately 100m)	2020	Random Forest model with 24 covariates and dasymetric redistribution.	Roads, land cover, built structures, cities or urban areas, nighttime lights, infrastructure, environmental data, protected areas and water bodies.	Open access	12

Supplementary Table 3 – Relative accessibility coverage per country (1-hour travel time).

Country	HRSL	GHS-POP	GPWv4	LandScan	WorldPop constrained	WorldPop unconstrained
Angola	84	91.1	62.7	74.6	85.2	77.1
Benin	96.3	97.9	82.2	95.9	93.7	88.8
Botswana	97.5	96.9	57.6	93.8	95.8	81.2
Burkina Faso	91.1	93.2	79.5	88.7	88.2	84.6
Burundi	99.9	98.8	98.2	99.2	99.9	98.6
Cameroon	96.9	97.7	69.8	94.1	95.7	84.6
Cape Verde		99.6	98.5	99.6	99.1	98.9
Central African Republic	93.5	94.3	49.4	85.9	92.1	75.4
Chad	73.8	87.7	44.7	74.1	66.9	53.4
Comoros	99.9	99.9	80.2	100	99.5	89.1
Côte d'Ivoire	96	98	82.1	95.7	93.9	89.9
Democratic Republic of the Congo	93.5	95.4	57	89.1	90.2	79.3
Djibouti	96.8	90.8	64.8	94.1	89.1	79.4
Equatorial Guinea	96.1	97.5	39.6	82.8	94.3	49.5
Eritrea	81.9	75.5	40.9	79.7	68.5	62.5
Ethiopia		94.2	70.4	89.9	83.6	80.7
Gabon	94.4	94.6	46.2	90.5	93.7	54.6
Gambia	72	80.9	54.9	72.2	72.1	66.3
Ghana	98.3	98.8	88.2	97.5	96.3	93.5
Guinea	94.2	95.6	75.8	90.7	92.6	84.5
Guinea-Bissau	62.1	66.5	47.7	59.2	61.4	52.4
Kenya	97.3	93.1	90.3	94.7	95.1	92.1
Lesotho	99.2	97	90.9	96.9	98.4	92.8
Liberia	93.9	94	69.3	89.5	89.4	77.2
Madagascar	75.1	69.9	58.7	76.6	68.7	65
Malawi	99.5	97.5	97.6	97.5	99.1	97.4
Mali	92.5	89.7	83.1	89.3	91.9	87.2
Mauritania	79.9	77.3	61	72	74.3	71.2
Mauritius	98.1	97.9	96.9	98.1	98	97
Mozambique	85.9	85.8	63.4	79.8	83.6	76.1
Namibia	87.3	84.2	82.6	83	87	86.7
Niger	85.6	87.5	68.5	82.3	82.8	78.8
Nigeria	97.7	96.5	89.4	96.9	97	93.5
Republic of Congo	90.5	93.1	34.6	89.9	79.3	41.6
Rwanda	99.5	98.3	96.6	97.3	99.3	97.3
Sao Tome and Principe	99.3	96.9	90.1	99.7	99	91.1
Senegal	95.5	97.4	82.9	94.4	95.2	89.5
Seychelles	98.5	98.9	94.9	98.9	97.7	95.9
Sierra Leone	97.9	95.7	89	95.4	96.5	92.9
Somalia	100	86	62.6	81.8	84.3	70.5
South Africa	99.6	99.1	98.7	98	99.5	99.2
South Sudan		80.9	35.5	80.2	82	43.7
Sudan		54.6	18.8	46.2	40.7	33.2
Swaziland	99.6	90.9	96.9	98.6	99.5	97.2
Tanzania	96.1	93.3	86.7	94.2	95.1	91.7
Togo	97.9	97.5	89.6	96.7	97	93.9
Uganda	99.1	97.4	93.7	97.5	98.6	96.1
Zambia	89.3	89.7	64.5	86.5	86.7	76.4
Zimbabwe	97.9	88.1	89.6	96.7	97.4	93.6

Supplementary Table 4 – Comparison of accessibility coverage estimates for seven provinces in the Democratic Republic of the Congo for which bottom-up estimates were recently released. For each province we indicated the absolute number of people covered in 1-hour travel time, the relative number of people covered, and the percentage of the population falling on barriers (i.e. rivers and lakes).

	Haut-Katanga			Haut-Lomami			Ituri			Kasaï			Kasaï-Oriental			Lomami			Sud-Kivu		
	nr. covered	% covered	% barrier	nr. covered	% covered	% barrier	nr. covered	% covered	% barrier	nr. covered	% covered	% barrier	nr. covered	% covered	% barrier	nr. covered	% covered	% barrier	nr. covered	% covered	% barrier
HRSL	5,747,046	95.5	0.2	4,023,749	90.1	0.2	4,380,453	98.9	0.4	3,688,383	92.7	0.3	3,909,065	98.9	0.1	2,767,965	95.4	0	4,362,380	95.8	1.5
GHS-POP	5,789,005	98.3	0.8	4,023,916	92.1	0.3	4,320,053	99.4	0.5	3,684,768	93.7	0.6	3,858,273	99.4	0.4	2,697,294	94.8	0.2	4,221,054	94.5	4.8
GPWv4	5,263,534	63.8	1	2,560,661	41.6	0.9	3,782,259	77.6	1.2	1,995,482	40.4	0.7	4,084,164	92.7	3.5	2,302,284	71.2	0.9	3,227,441	65.6	3.3
GRID3	4,580,262	93.3	0.2	2,275,209	84.3	0.6	5,058,822	97.6	0.5	2,650,634	89.5	0.5	2,176,677	96.6	0.2	2,551,022	93.5	0	4,575,325	96.3	1.3
LandScan	3,076,949	93.7	0.7	1,678,176	80	0.8	5,132,628	97.8	1.1	3,030,880	85.7	0.6	3,394,953	97.5	1.1	2,755,803	93.6	0.2	5,807,295	87.5	5.7
WorldPop constrained	7,545,390	89.7	0.4	5,253,516	84	0.6	4,727,189	96.8	0.5	4,368,401	88.4	0.4	4,371,462	98.3	0.2	3,019,200	92.5	0.1	4,662,320	94.8	1.3
WorldPop unconstrained	7,167,589	85	1.1	4,145,649	66.3	0.8	4,542,688	93.1	1	3,688,820	74.7	0.5	4,389,662	97.2	1.4	2,856,164	87.4	0.6	4,238,609	86.2	2.2

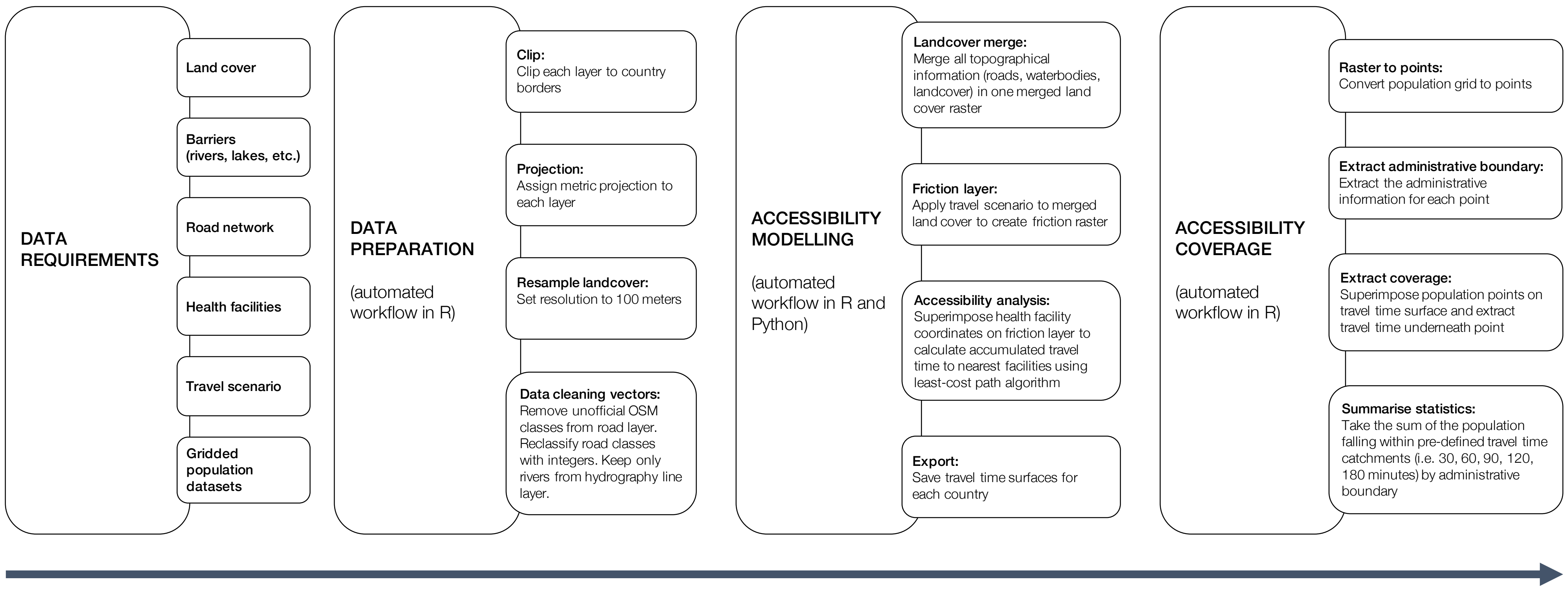

Supplementary Figure 1 – Visual overview of methodology with successive steps from left to right.

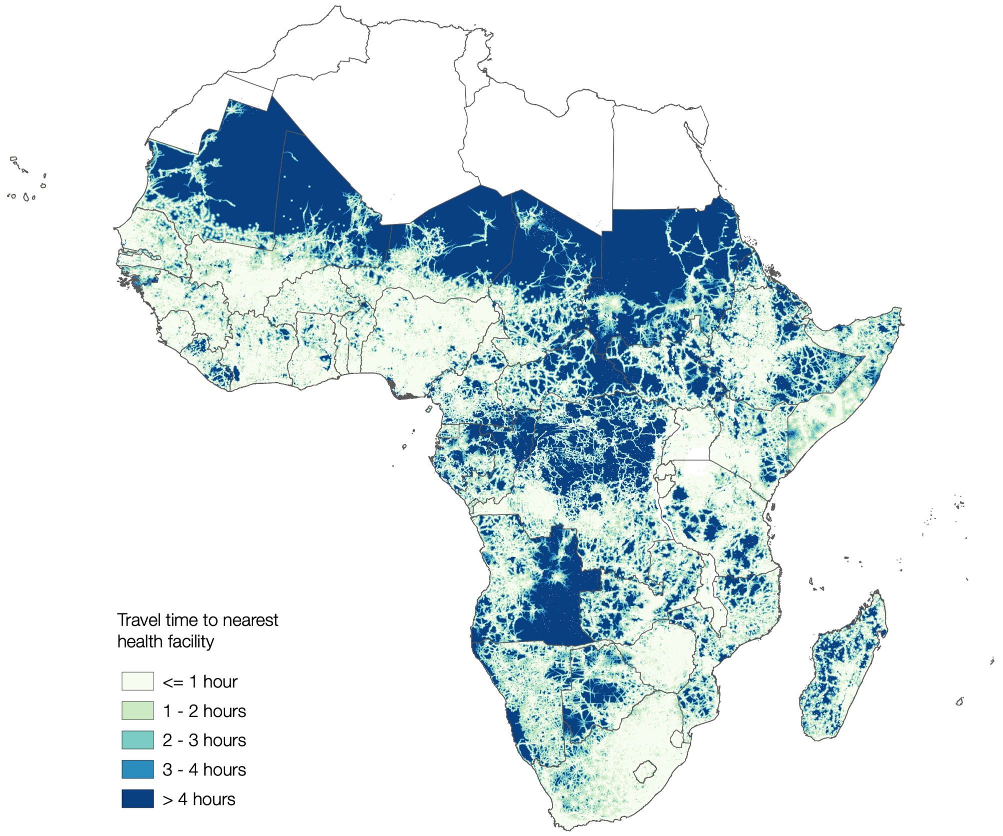

Supplementary Figure 2 – Travel time to nearest health facilities in sub-Saharan Africa (considering walking and motorized transport) for the first time at a resolution of 100 meters for sub-Saharan Africa. We would like to stipulate that widescale assumptions on travel speeds and modes have been made to construct the national maps of travel time to healthcare. These assumptions may not correspond to local realities of health seeking behavior. We therefore recognize that these maps should not be used for informed health system planning.

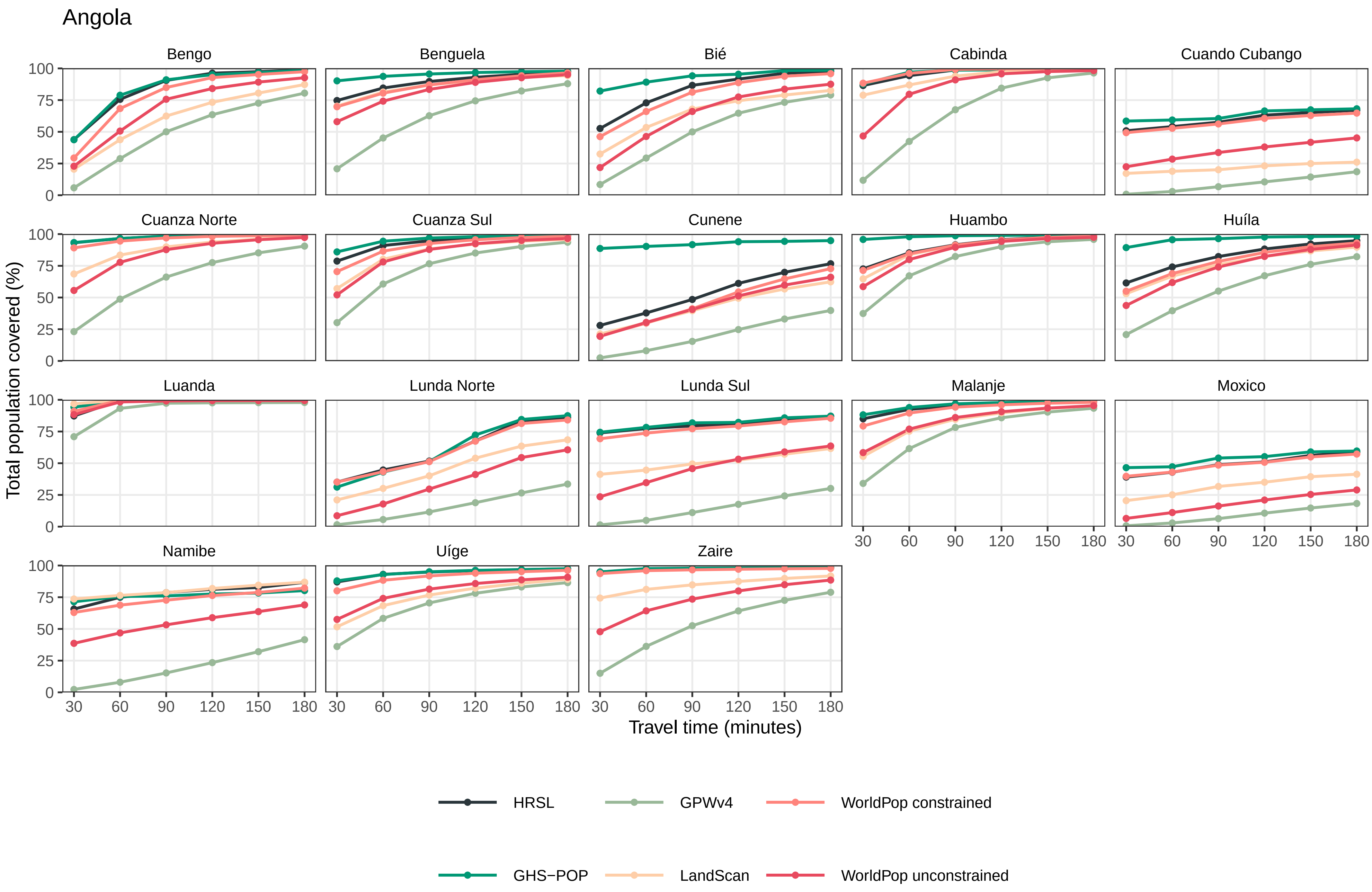

Supplementary Figure 3 − Accessibility coverage at 30, 60, 90, 120, and 150 minutes travel time at administrative level 1 for Angola.

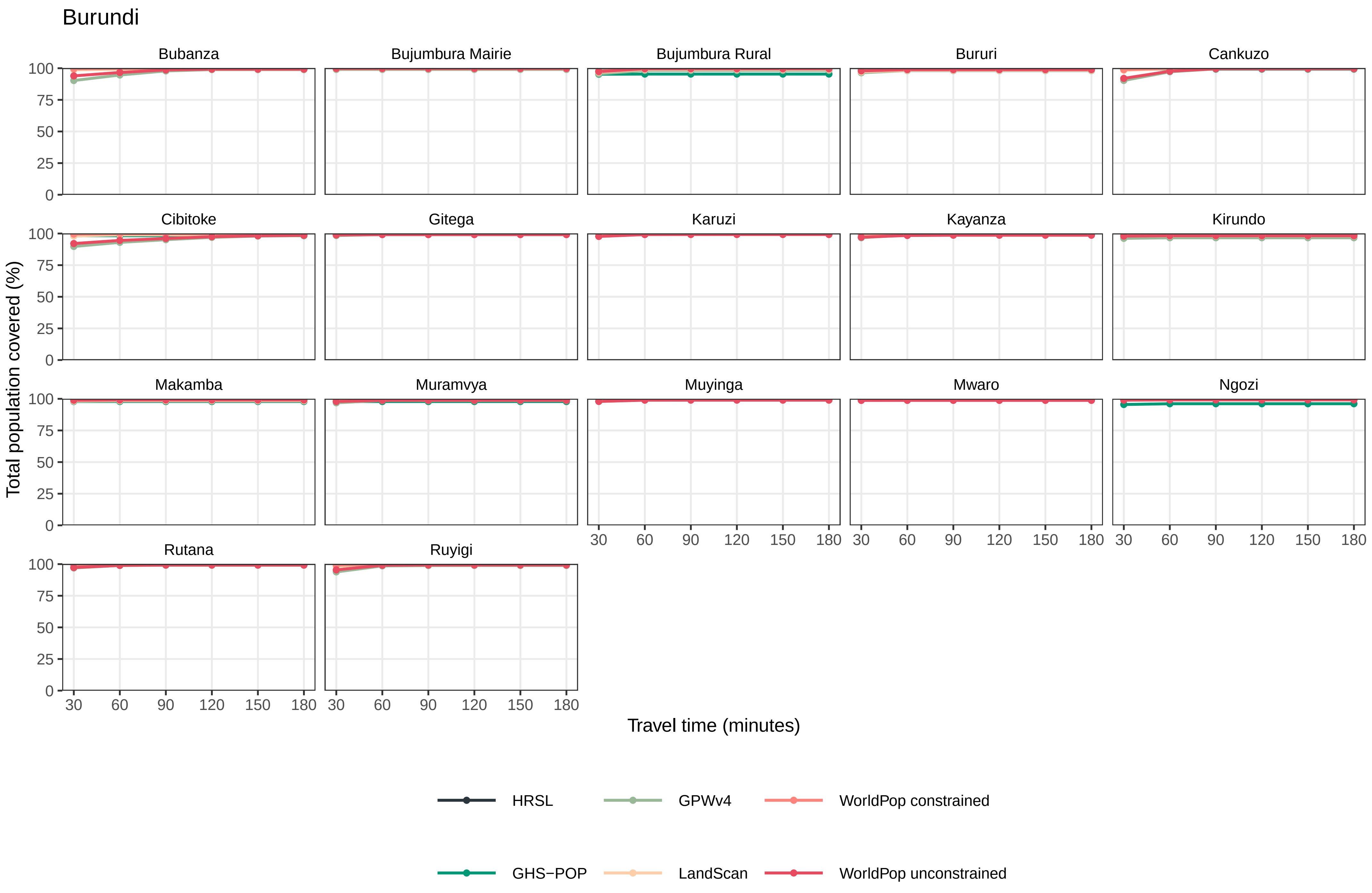

Supplementary Figure 4 − Accessibility coverage at 30, 60, 90, 120, and 150 minutes travel time at administrative level 1 for Burundi.

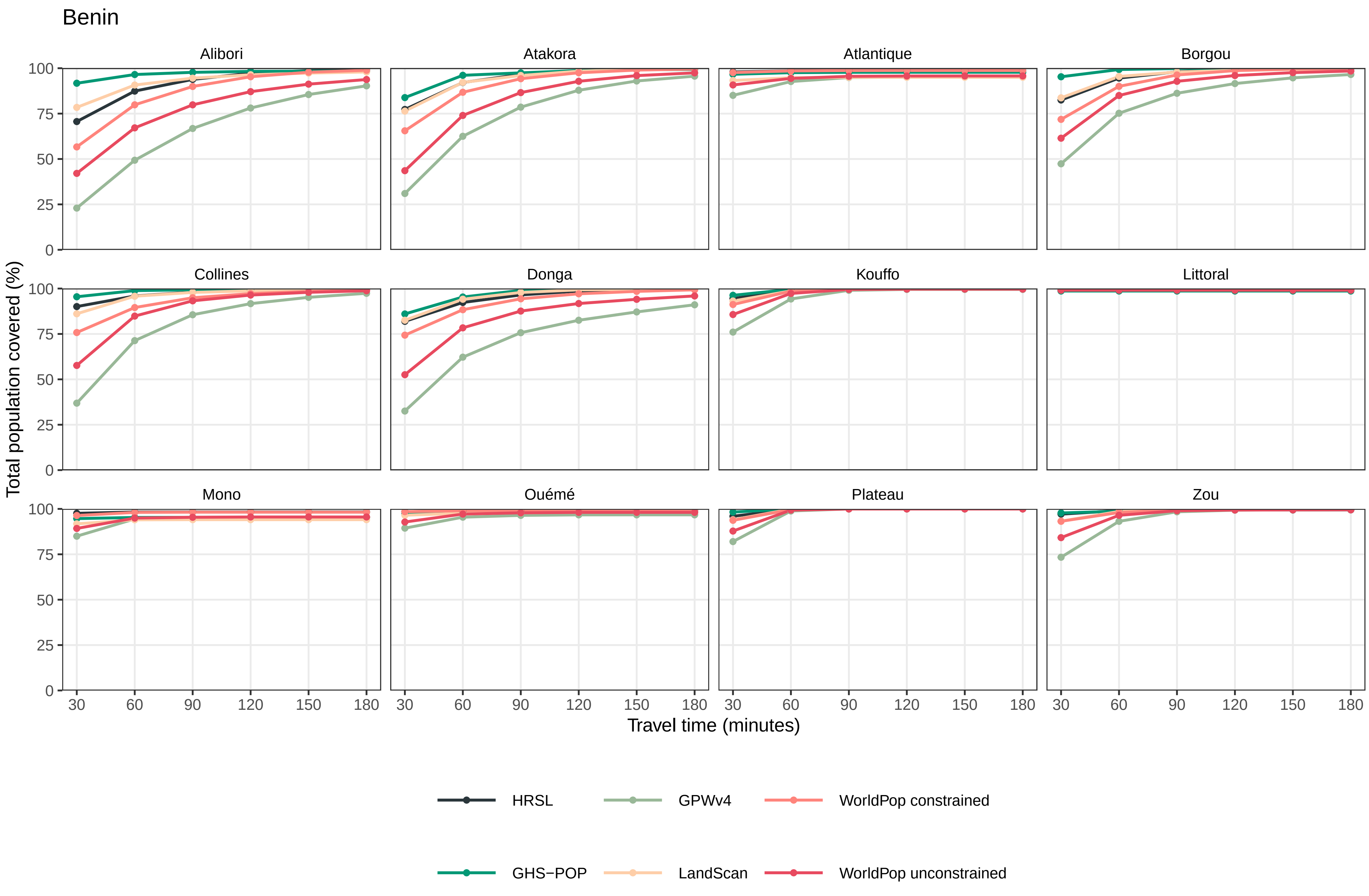

Supplementary Figure 5 − Accessibility coverage at 30, 60, 90, 120, and 150 minutes travel time at administrative level 1 for Benin.

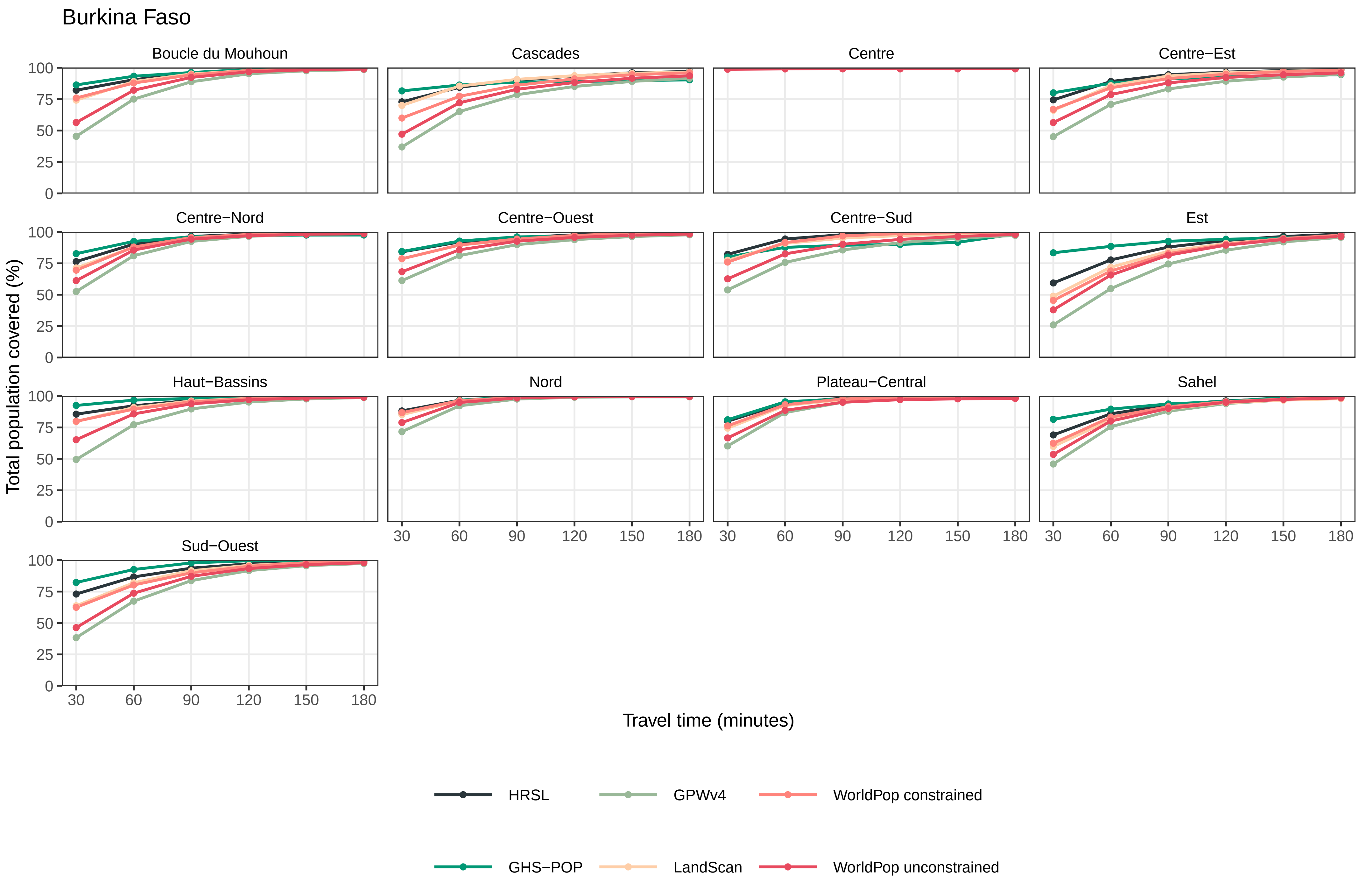

Supplementary Figure 6 − Accessibility coverage at 30, 60, 90, 120, and 150 minutes travel time at administrative level 1 for Burkina Faso.

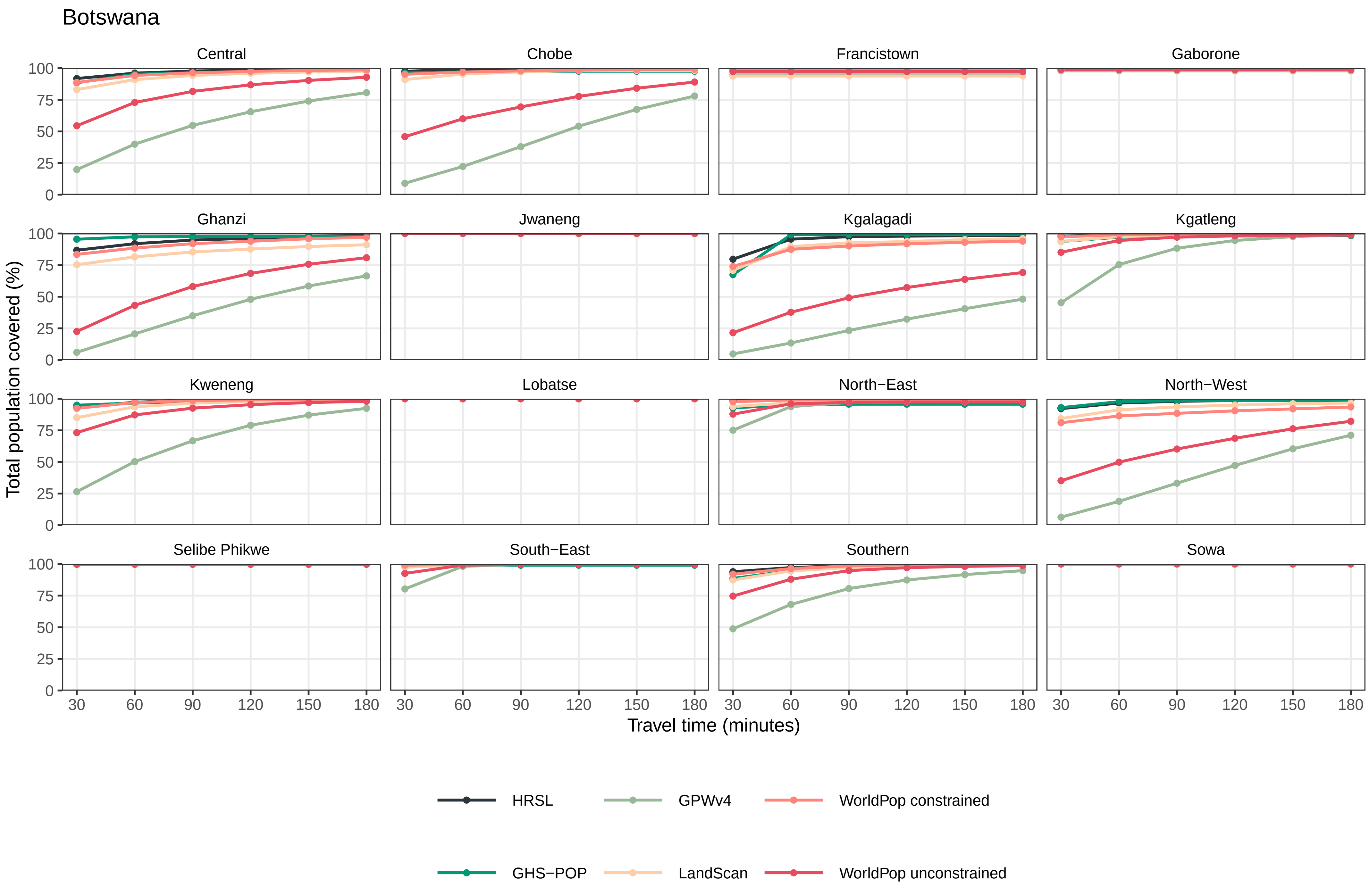

Supplementary Figure 7 − Accessibility coverage at 30, 60, 90, 120, and 150 minutes travel time at administrative level 1 for Botswana.

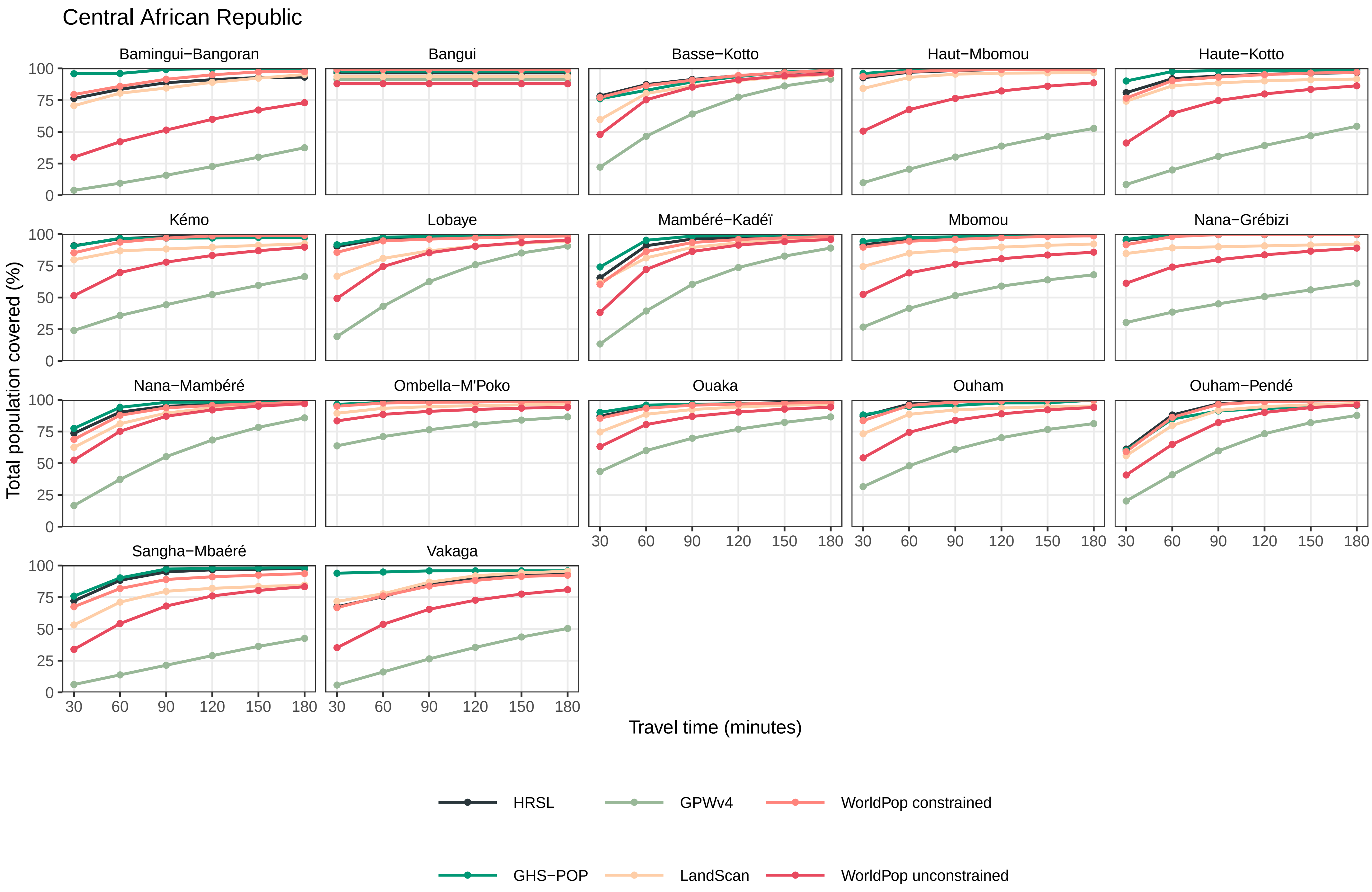

Supplementary Figure 8 − Accessibility coverage at 30, 60, 90, 120, and 150 minutes travel time at administrative level 1 for Central African Republic.

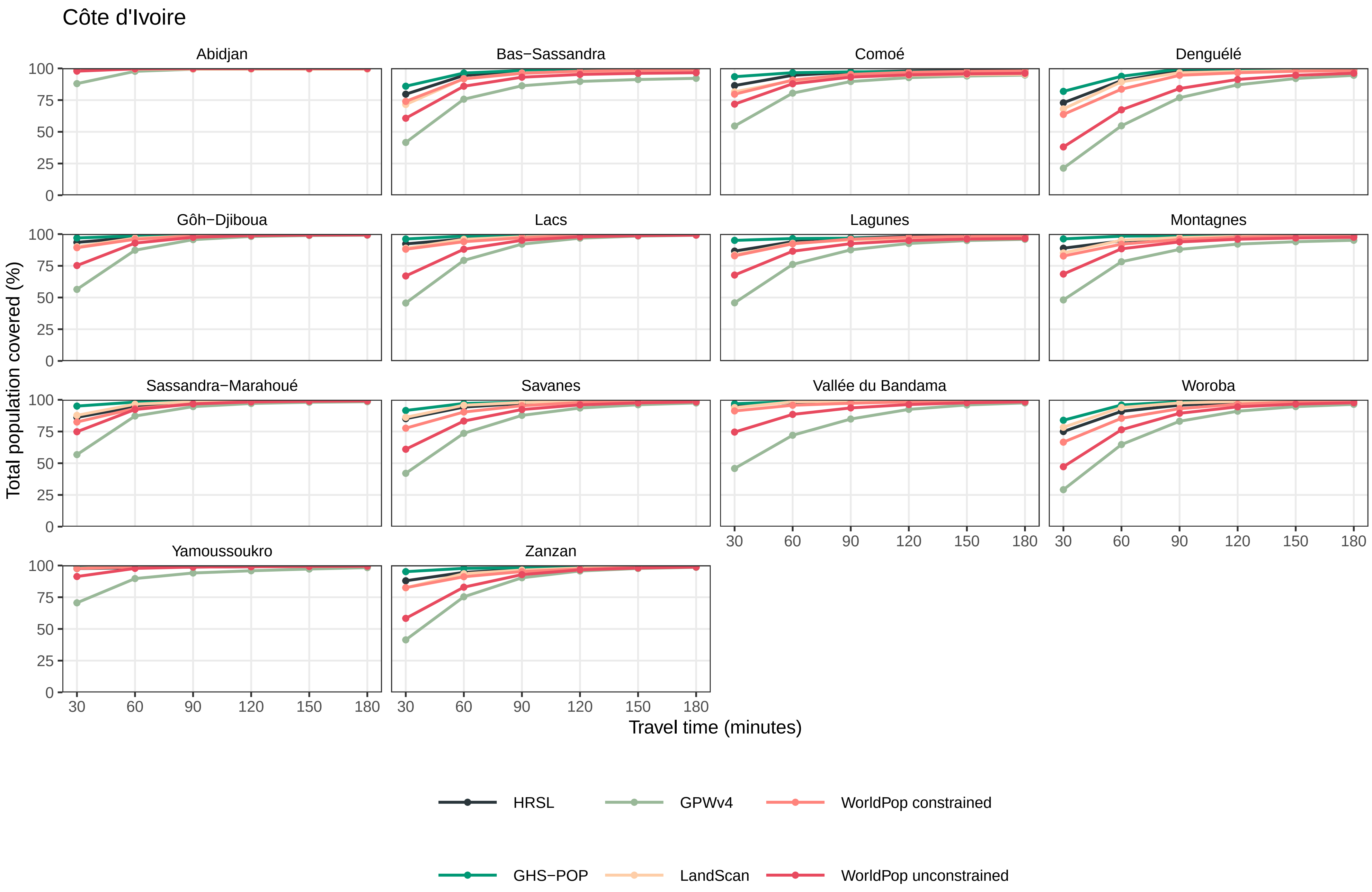

Supplementary Figure 9 − Accessibility coverage at 30, 60, 90, 120, and 150 minutes travel time at administrative level 1 for Côte d'Ivoire.

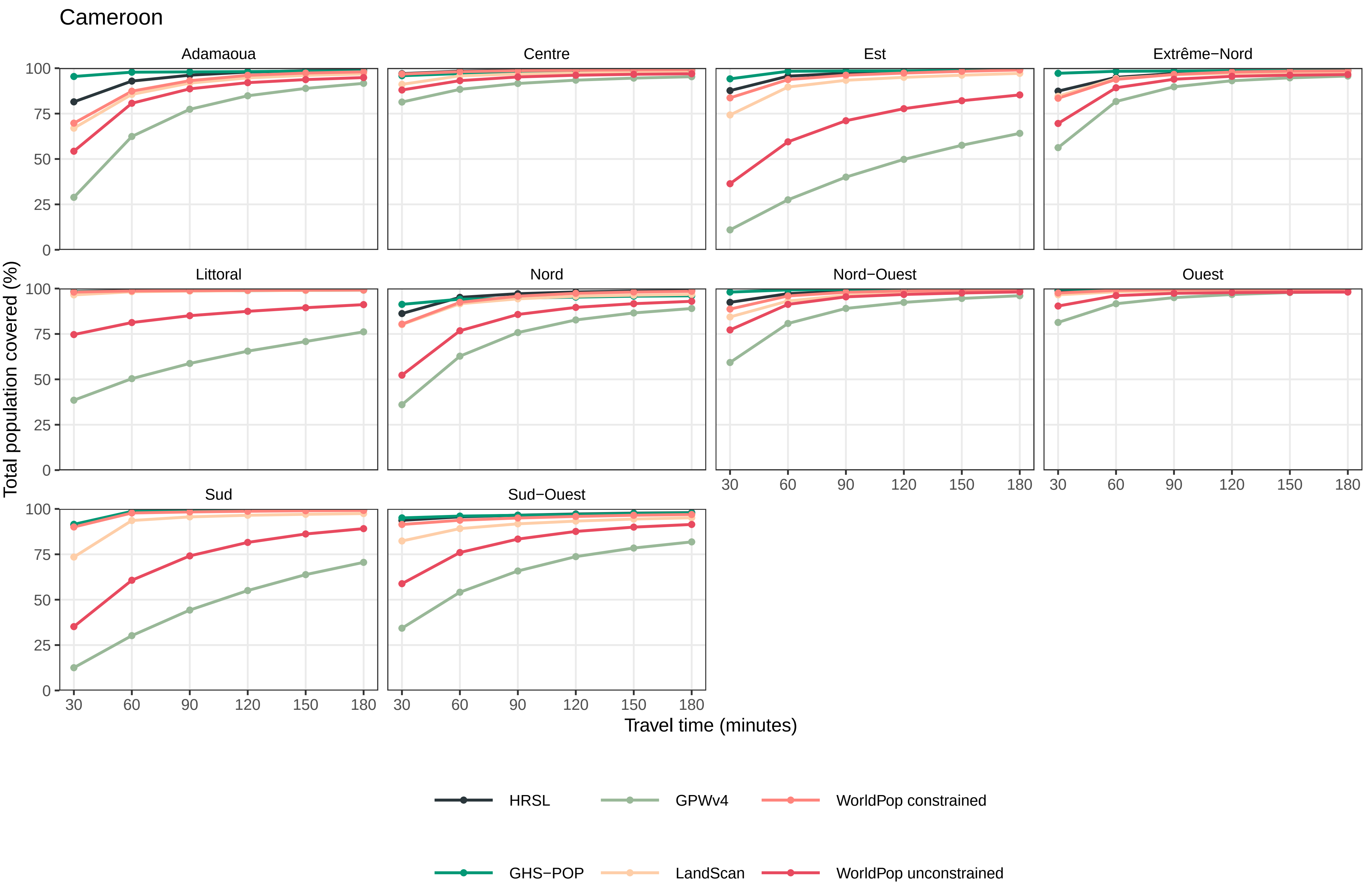

Supplementary Figure 10 − Accessibility coverage at 30, 60, 90, 120, and 150 minutes travel time at administrative level 1 for Cameroon.

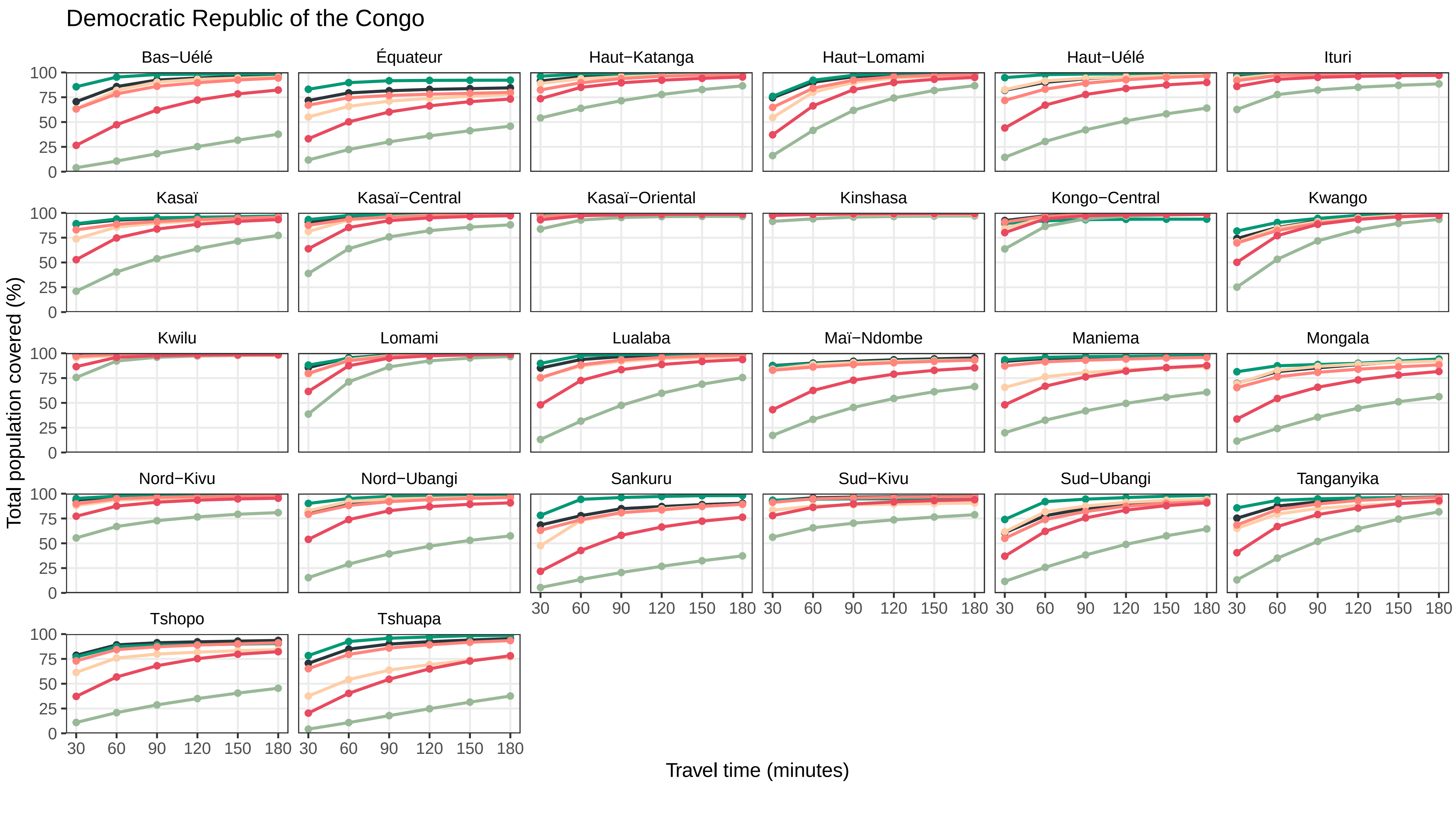

Supplementary Figure 11 − Accessibility coverage at 30, 60, 90, 120, and 150 minutes travel time at administrative level 1 for Democratic Republic of the Cong

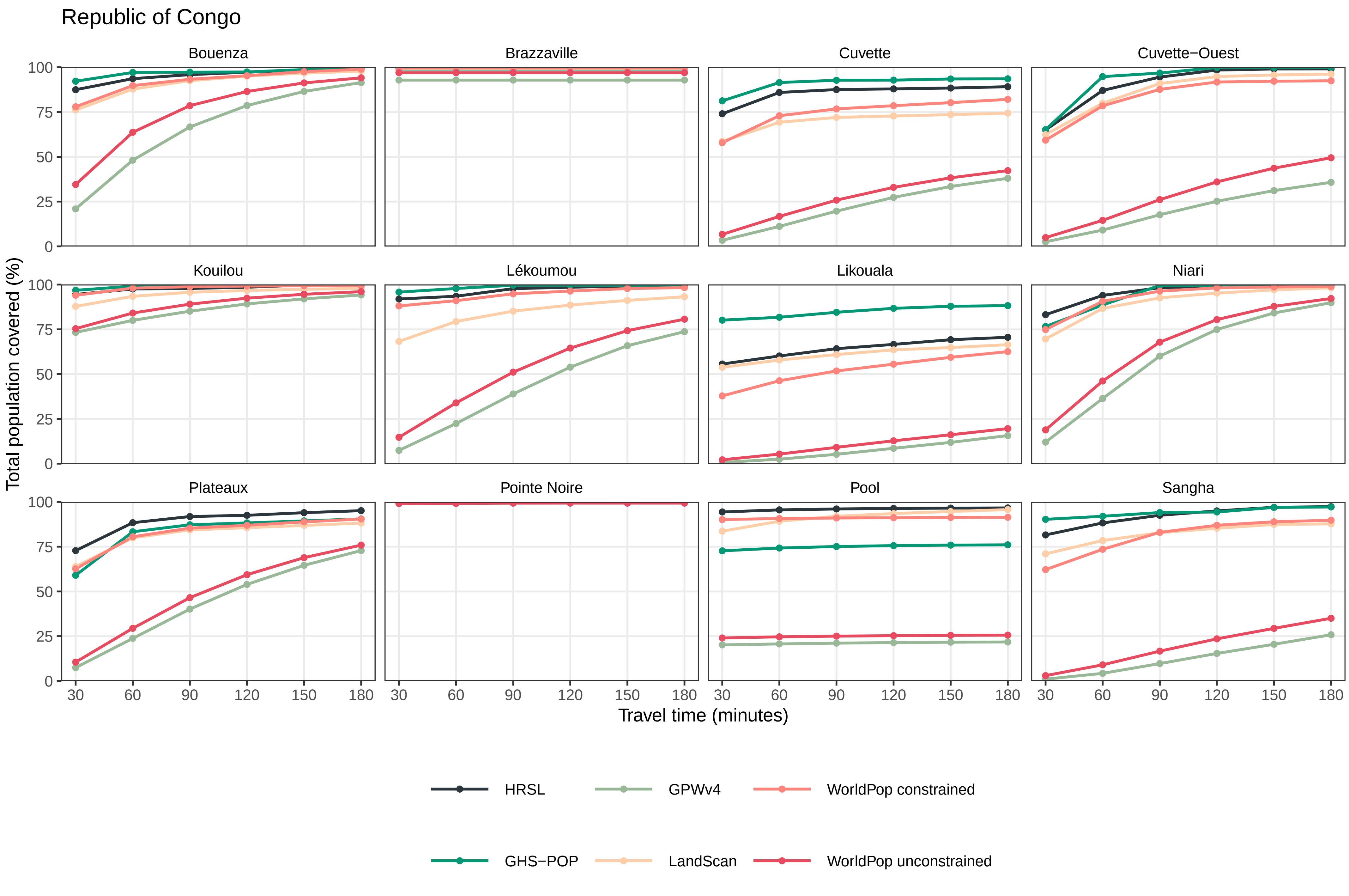

Supplementary Figure 12 − Accessibility coverage at 30, 60, 90, 120, and 150 minutes travel time at administrative level 1 for Republic of Congo.

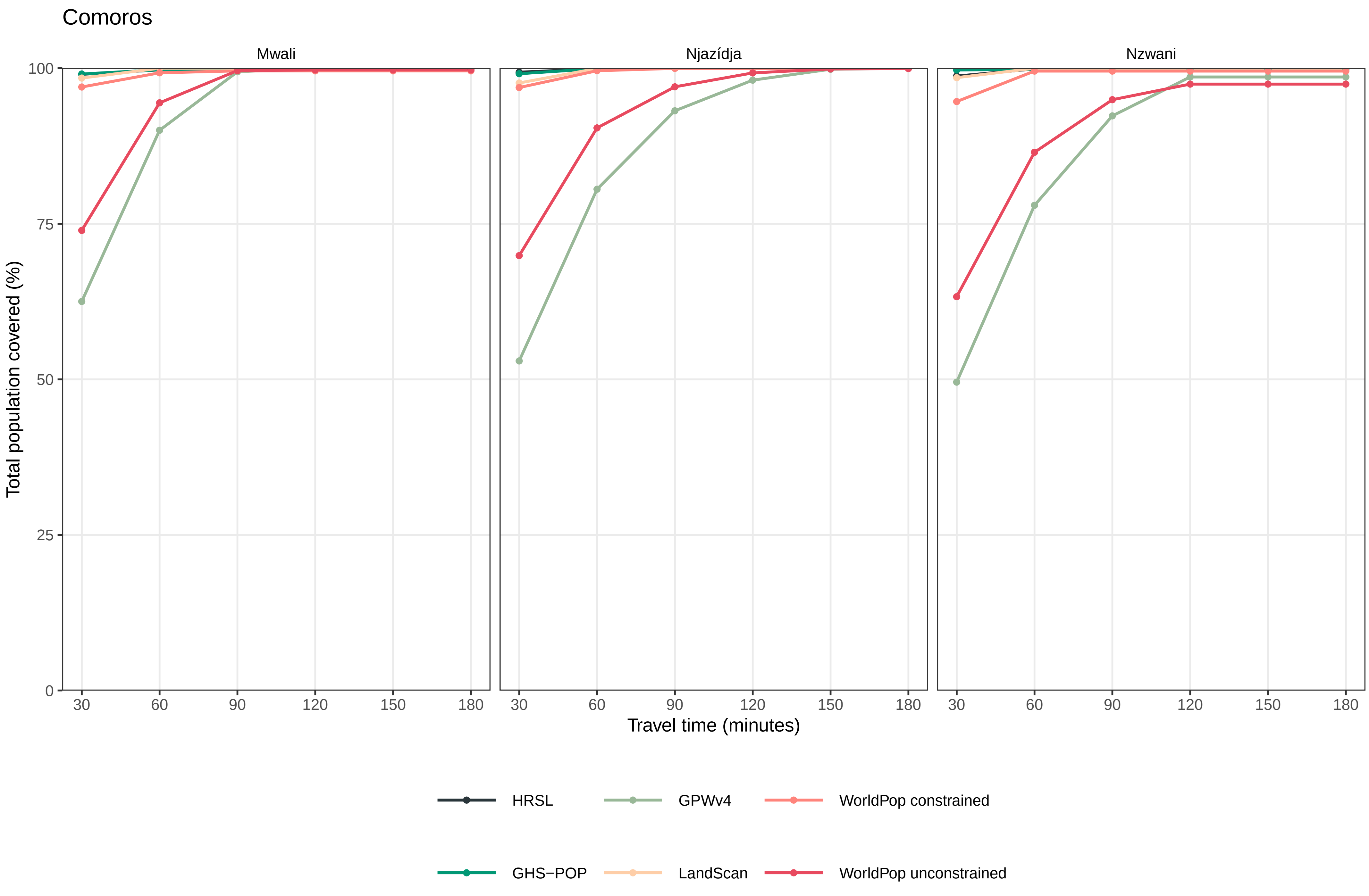

Supplementary Figure 13 − Accessibility coverage at 30, 60, 90, 120, and 150 minutes travel time at administrative level 1 for Comoros.

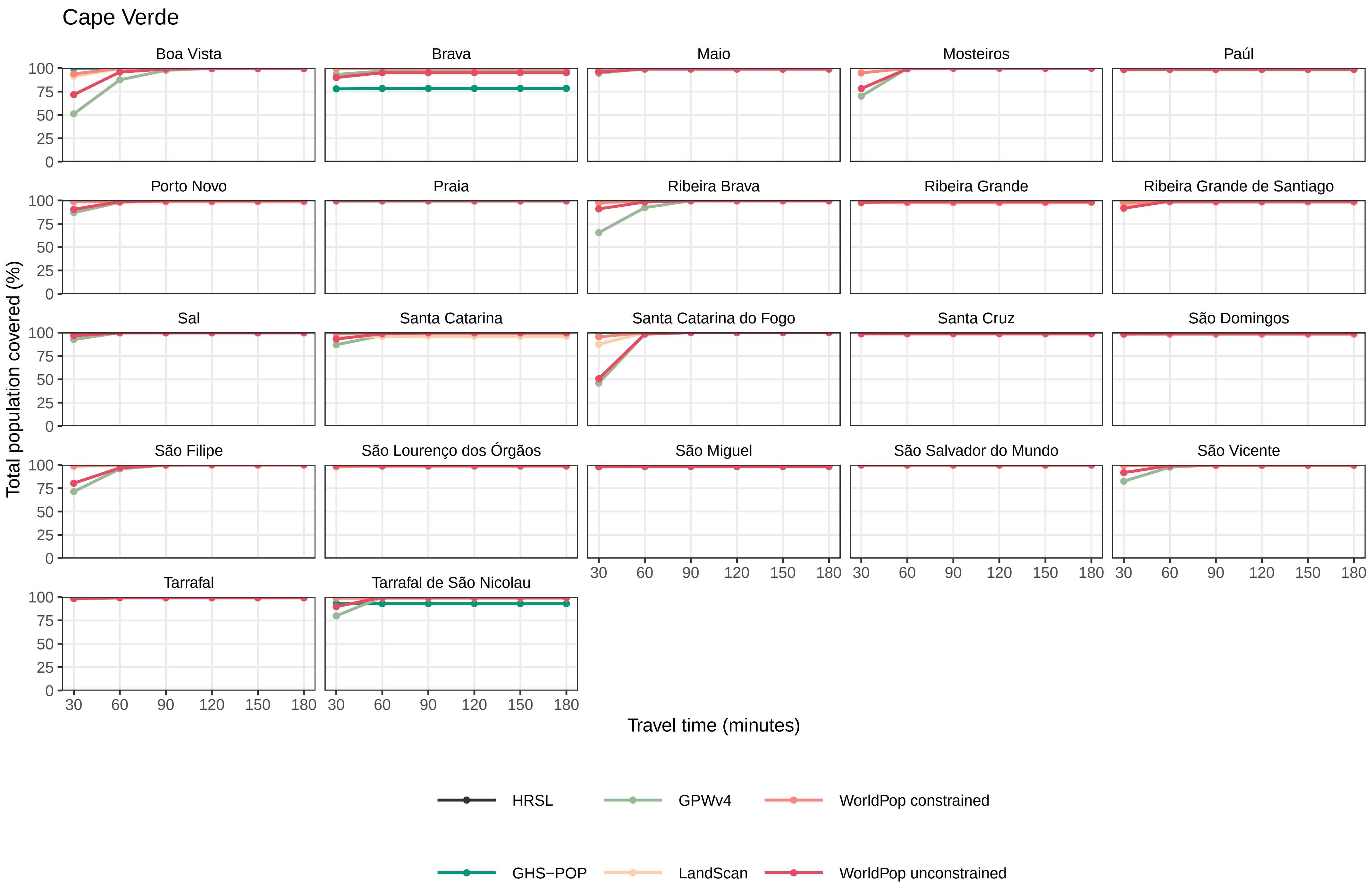

Supplementary Figure 14 − Accessibility coverage at 30, 60, 90, 120, and 150 minutes travel time at administrative level 1 for Cape Verde.

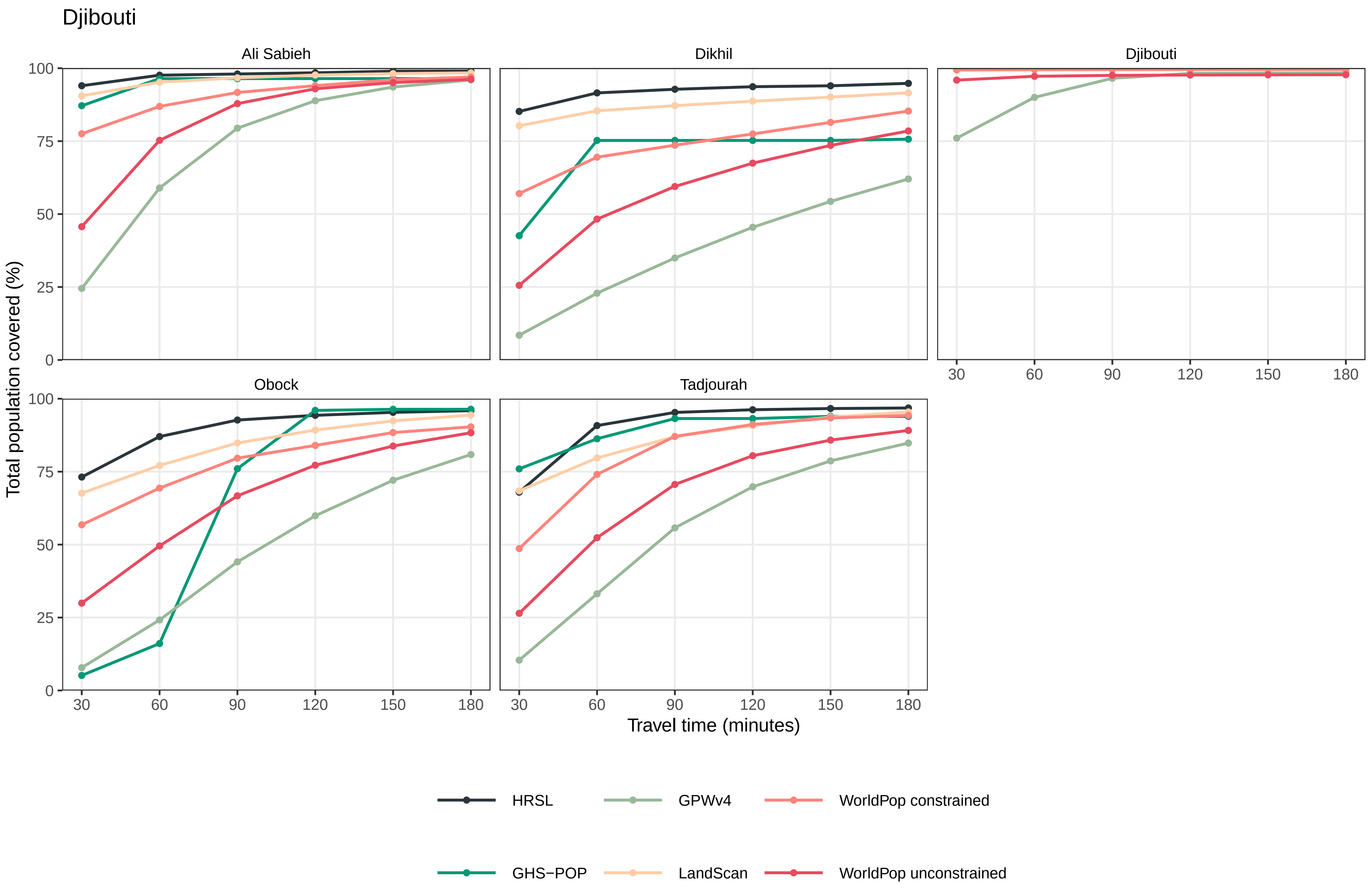

Supplementary Figure 15 − Accessibility coverage at 30, 60, 90, 120, and 150 minutes travel time at administrative level 1 for Djibouti.

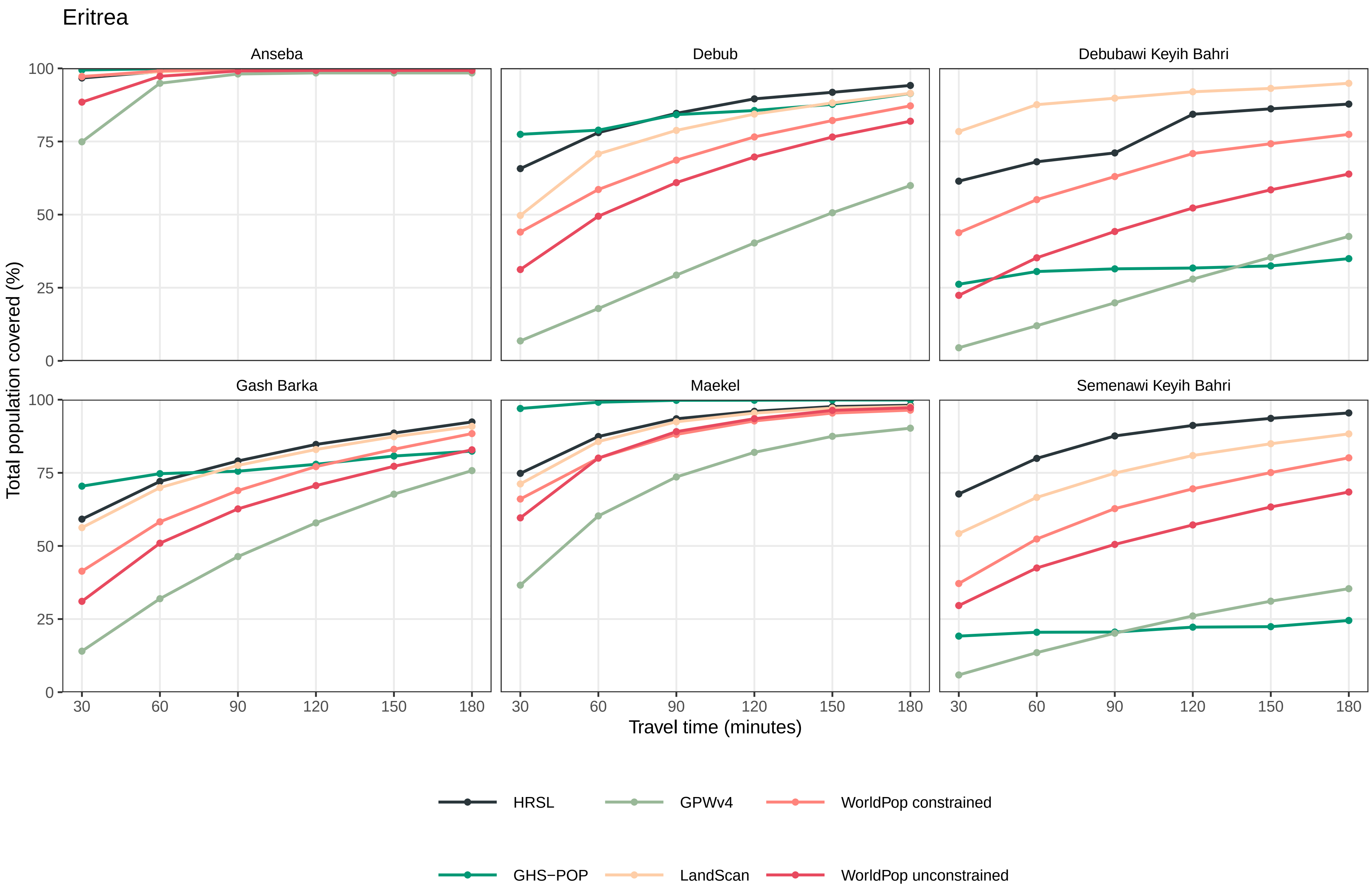

Supplementary Figure 16 − Accessibility coverage at 30, 60, 90, 120, and 150 minutes travel time at administrative level 1 for Eritrea.

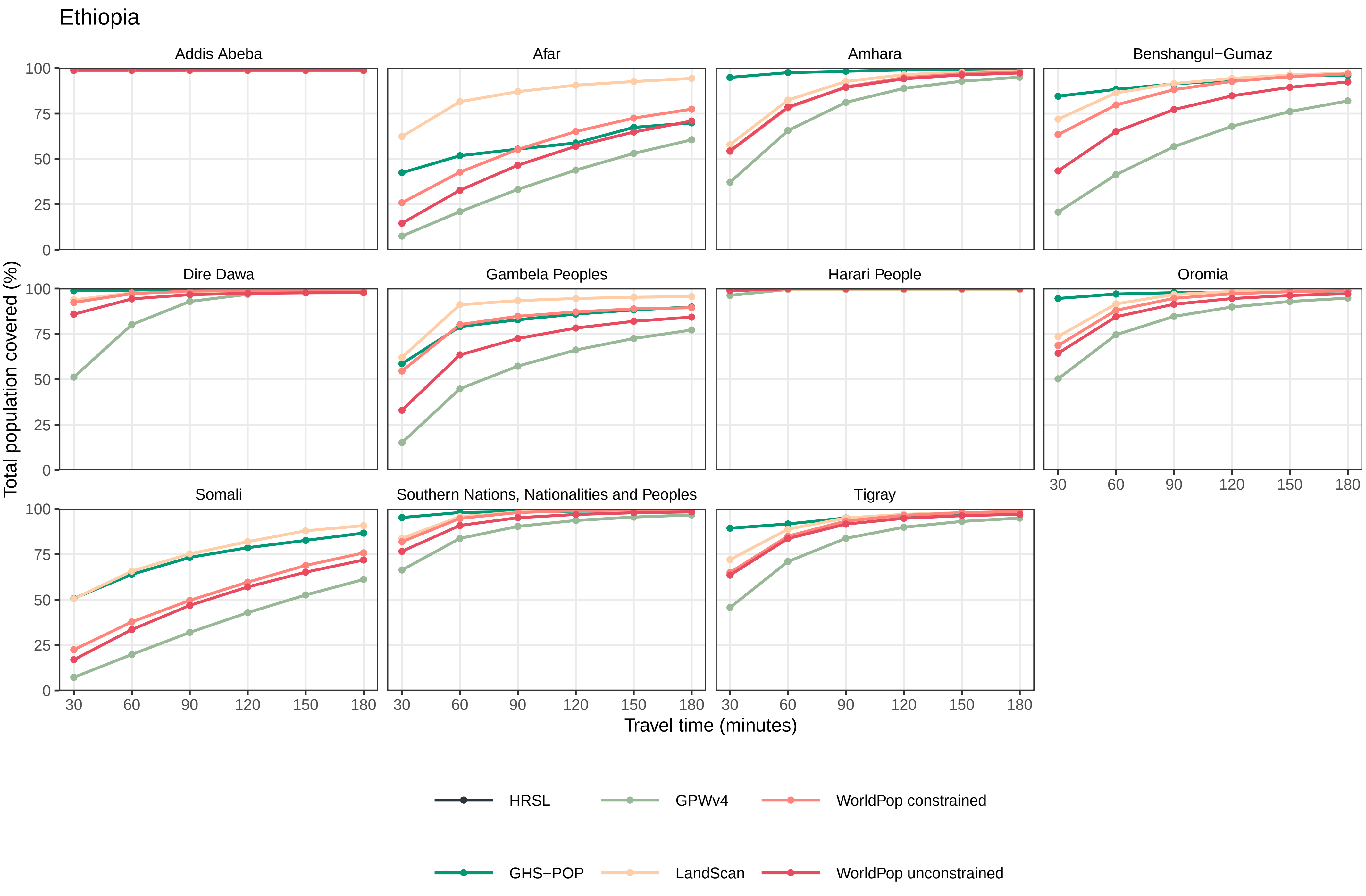

Supplementary Figure 17 − Accessibility coverage at 30, 60, 90, 120, and 150 minutes travel time at administrative level 1 for Ethiopia.

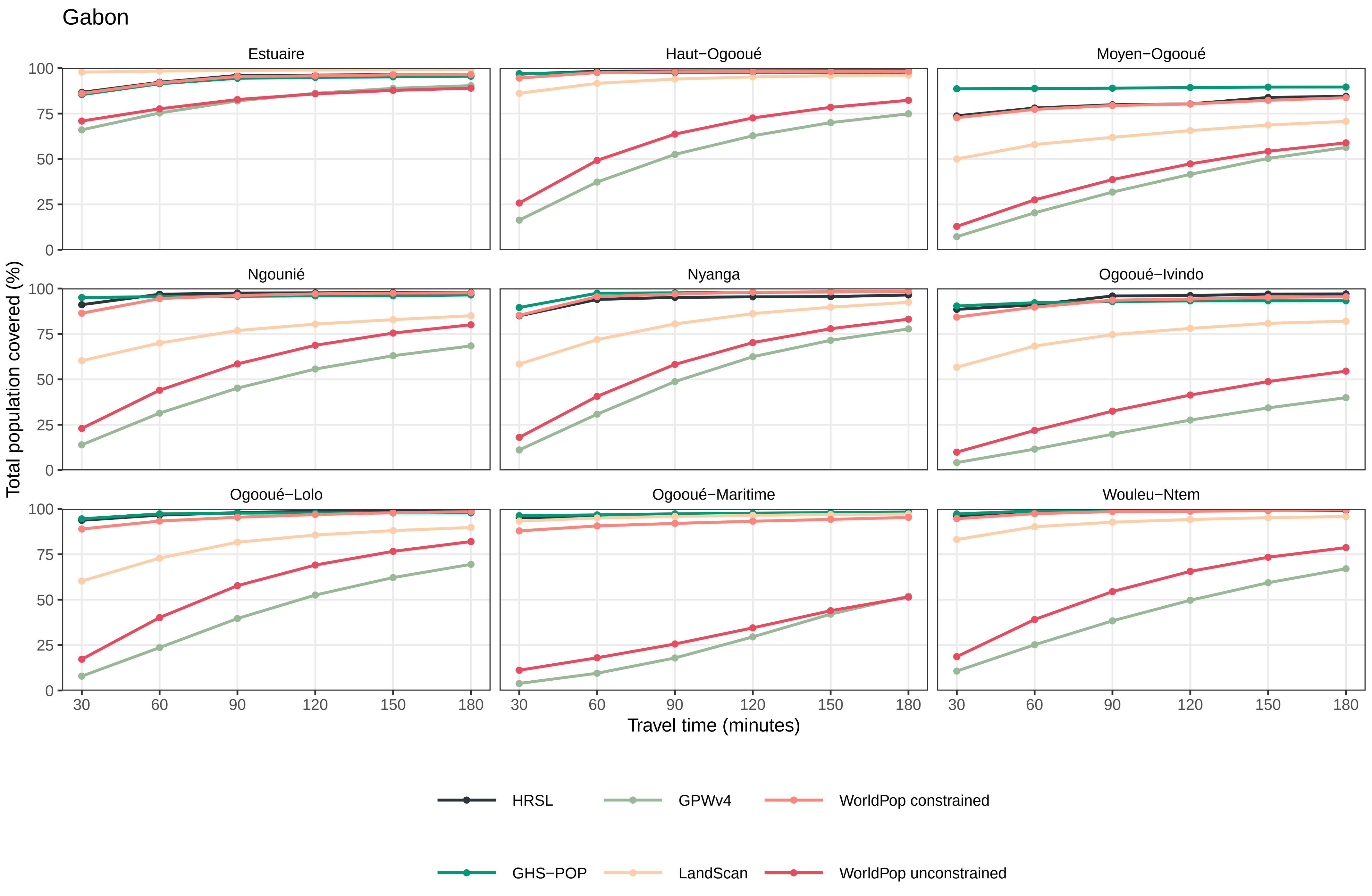

Supplementary Figure 18 − Accessibility coverage at 30, 60, 90, 120, and 150 minutes travel time at administrative level 1 for Gabon.

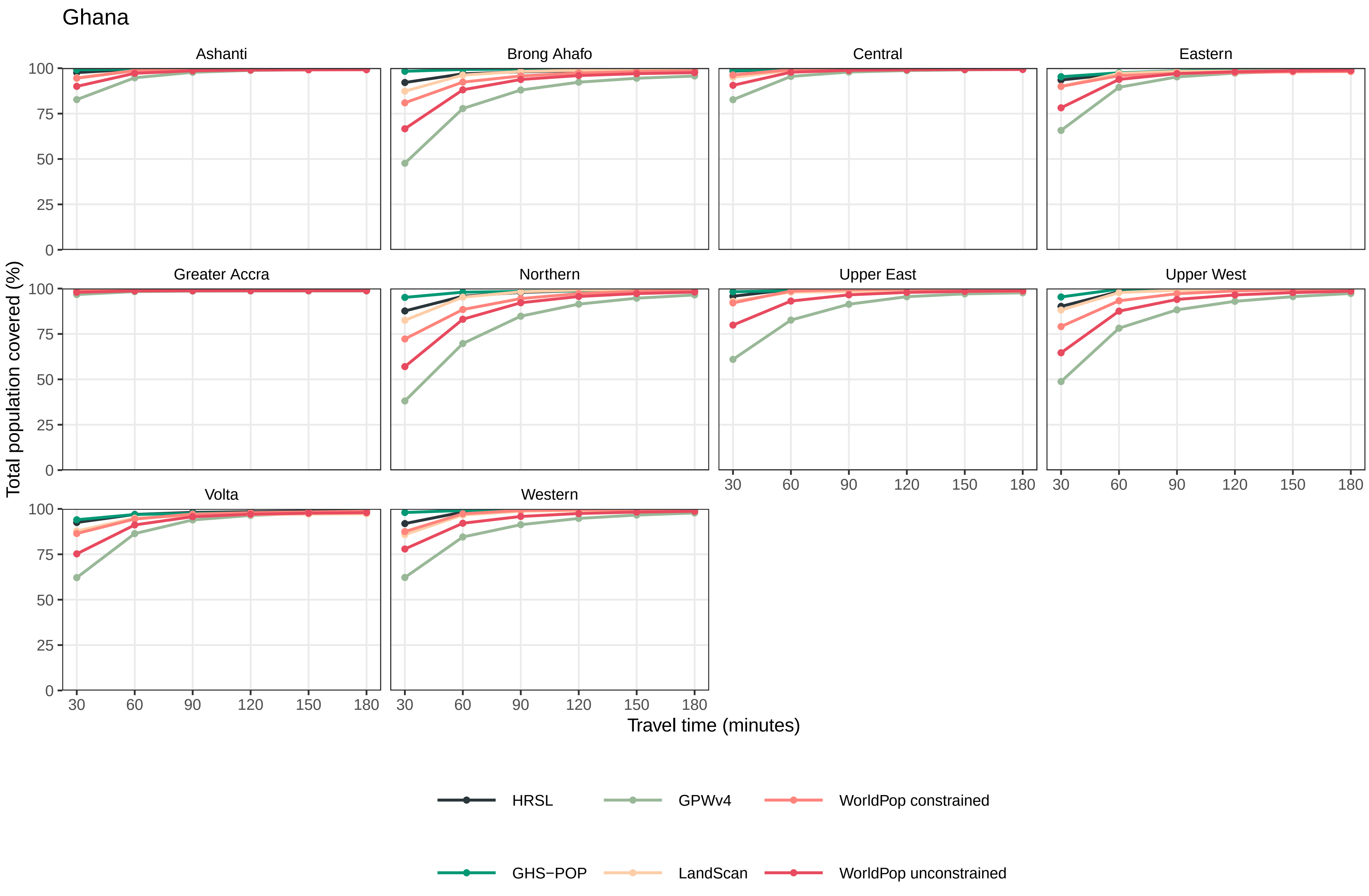

Supplementary Figure 19 − Accessibility coverage at 30, 60, 90, 120, and 150 minutes travel time at administrative level 1 for Ghana.

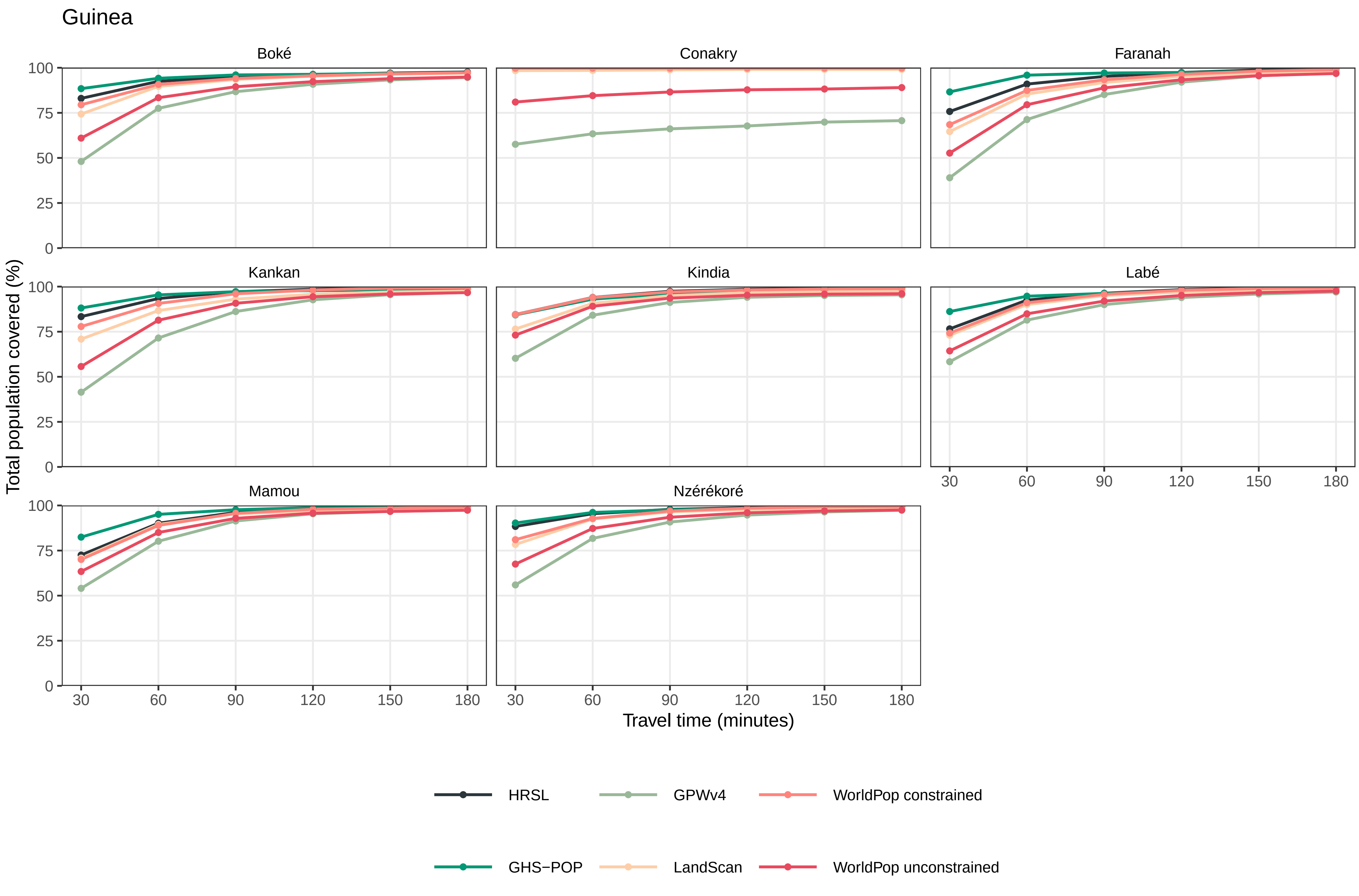

Supplementary Figure 20 − Accessibility coverage at 30, 60, 90, 120, and 150 minutes travel time at administrative level 1 for Guinea.

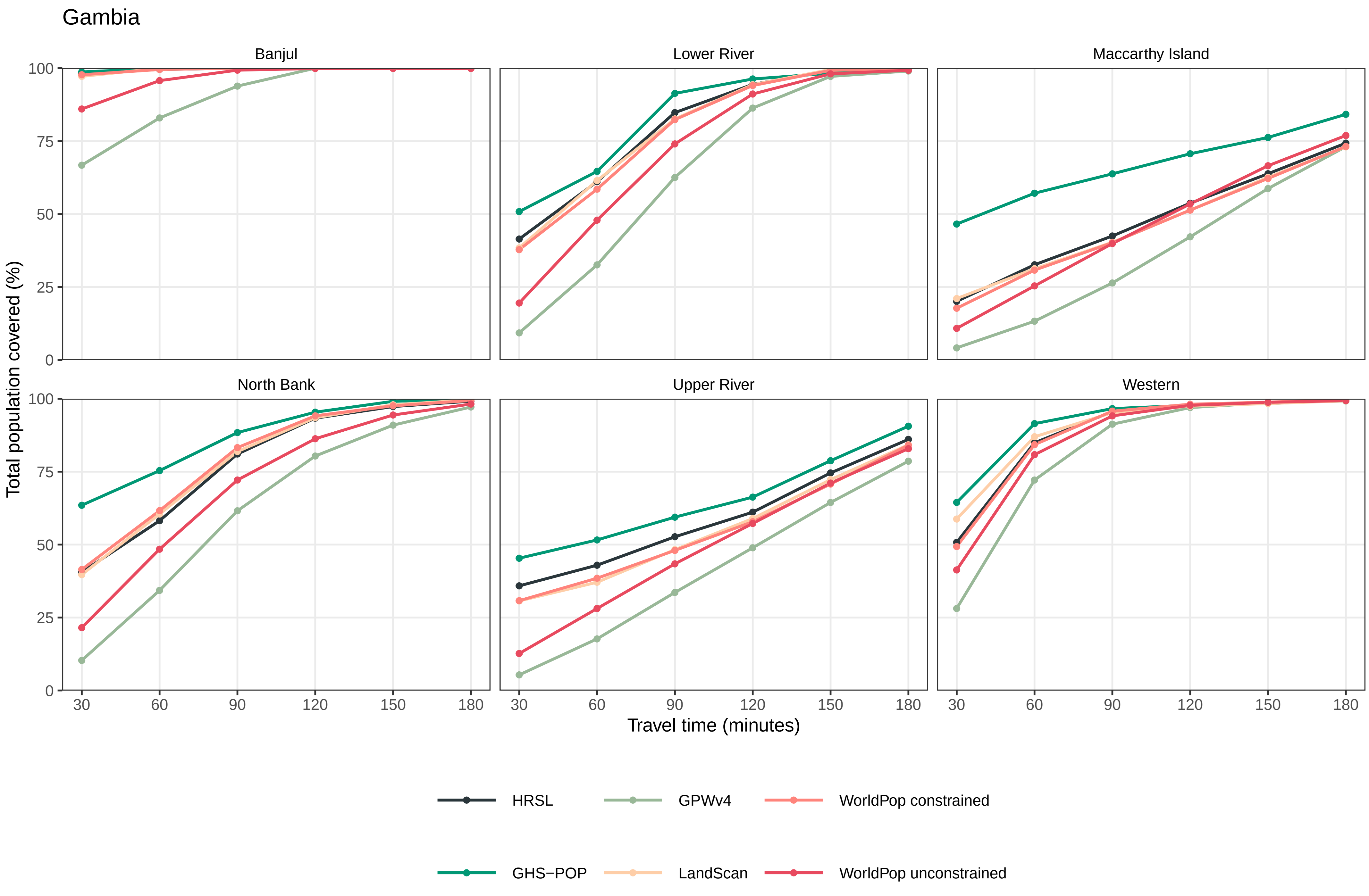

Supplementary Figure 21 − Accessibility coverage at 30, 60, 90, 120, and 150 minutes travel time at administrative level 1 for Gambia.

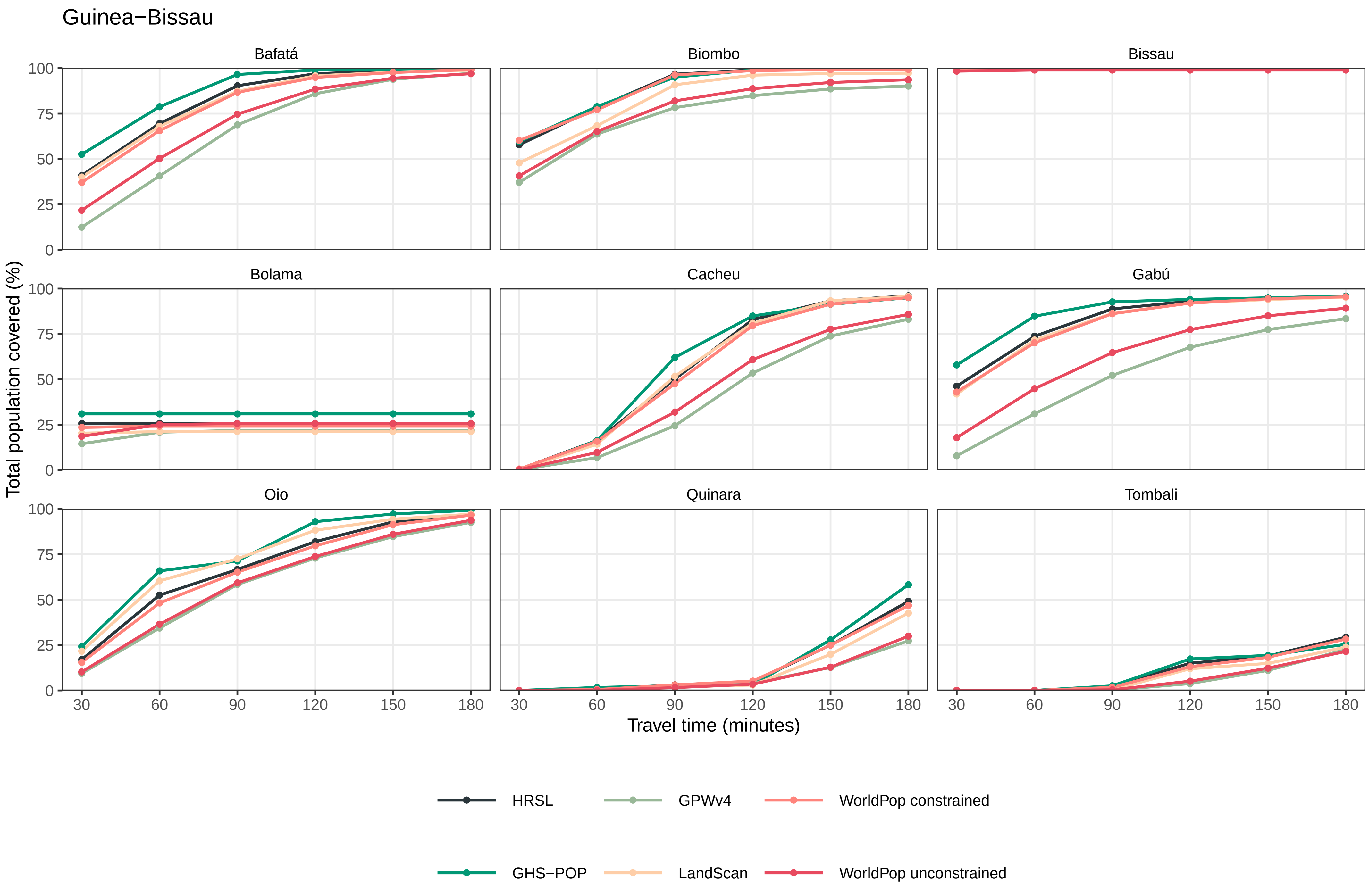

Supplementary Figure 22 − Accessibility coverage at 30, 60, 90, 120, and 150 minutes travel time at administrative level 1 for Guinea−Bissau.

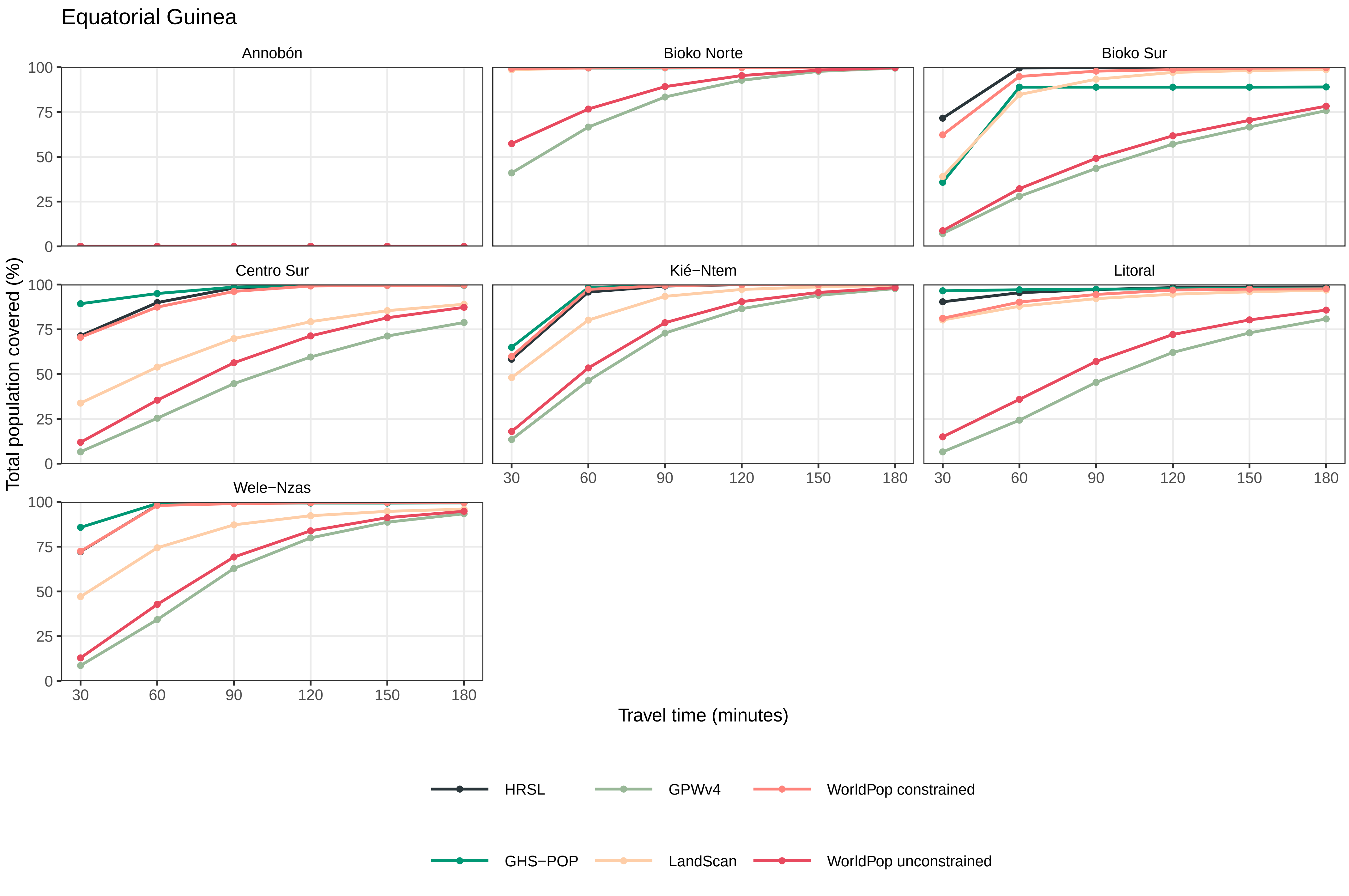

Supplementary Figure 23 − Accessibility coverage at 30, 60, 90, 120, and 150 minutes travel time at administrative level 1 for Equatorial Guinea.

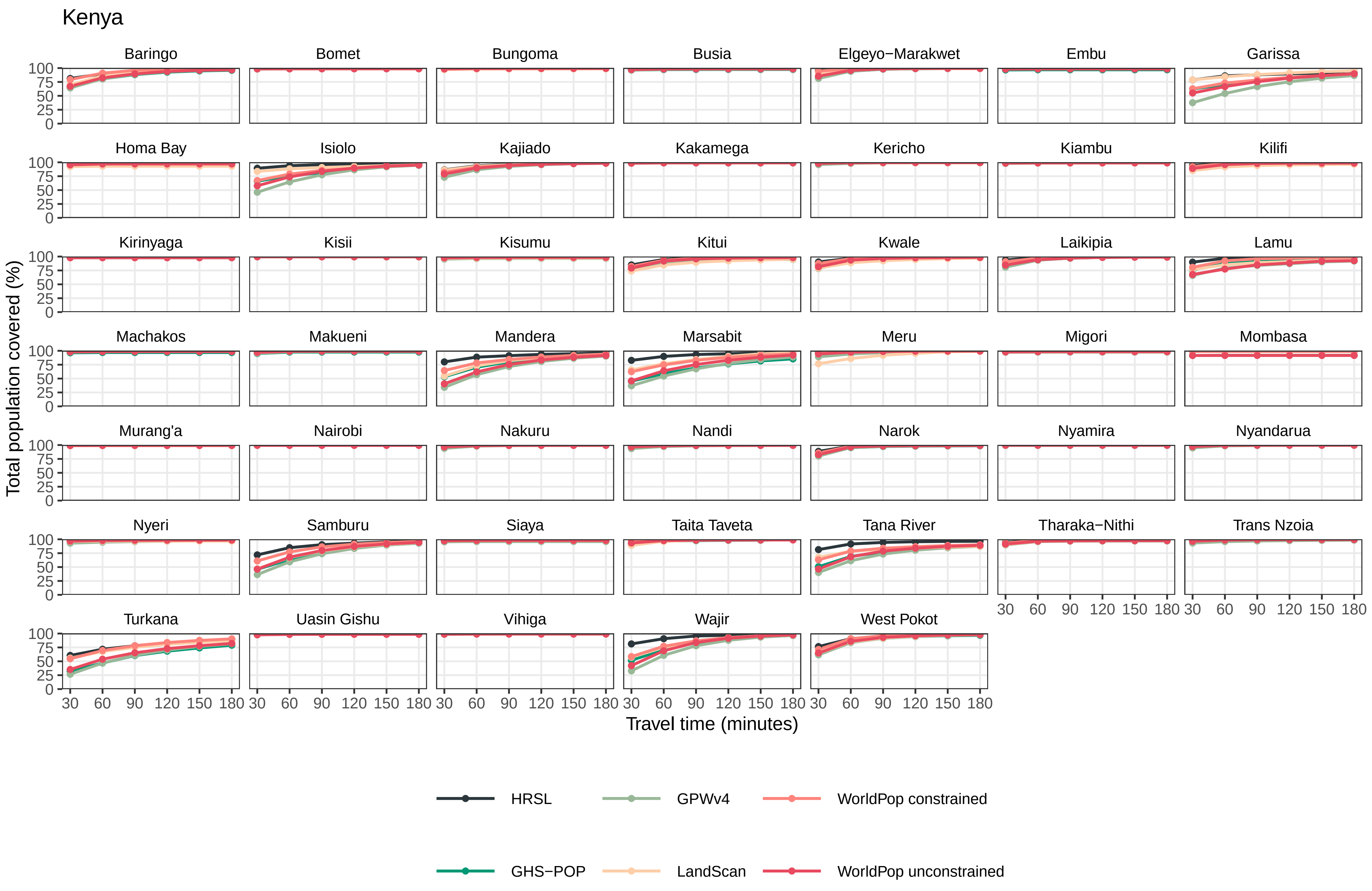

Supplementary Figure 24 − Accessibility coverage at 30, 60, 90, 120, and 150 minutes travel time at administrative level 1 for Kenya.

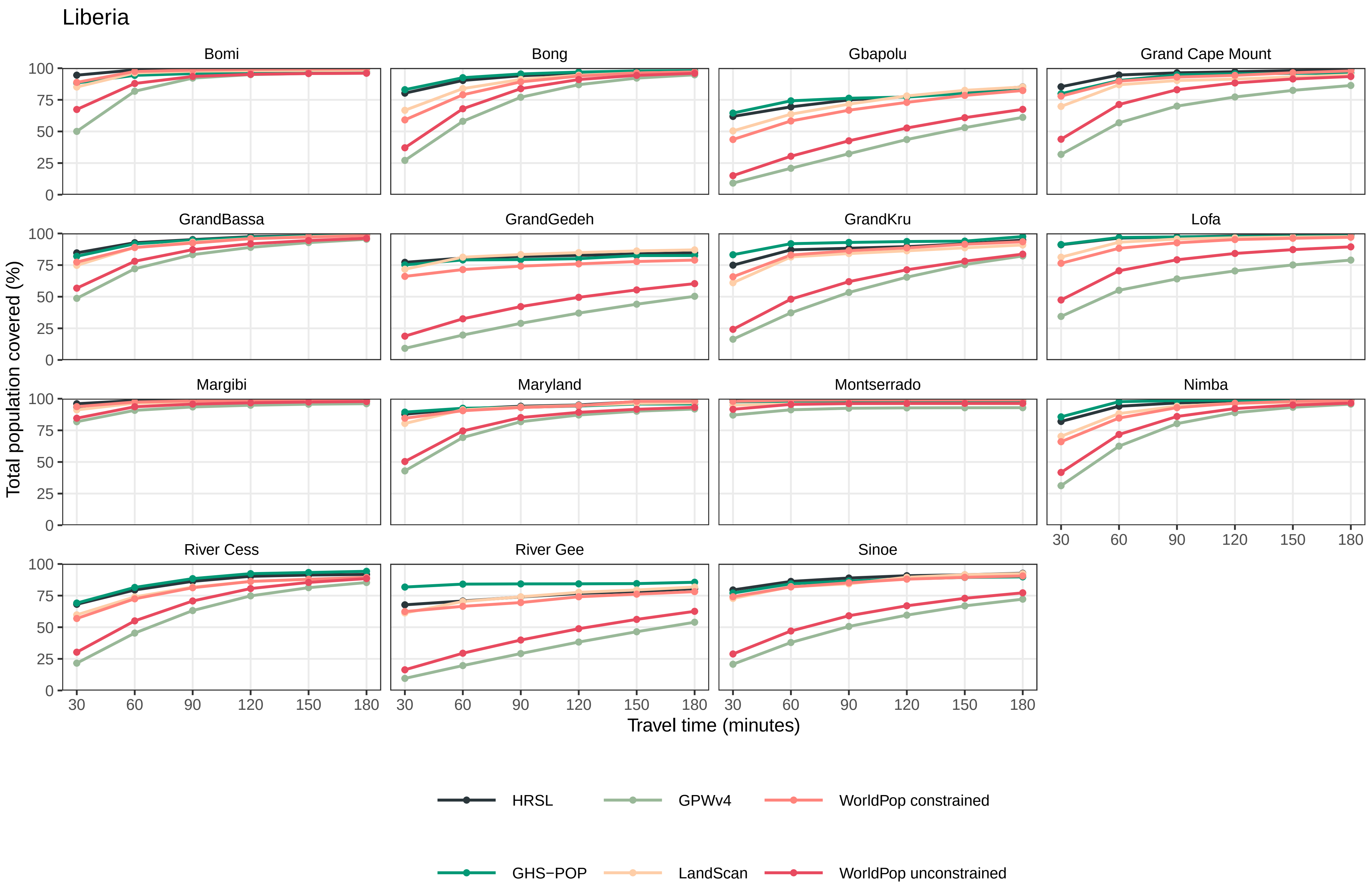

Supplementary Figure 25 − Accessibility coverage at 30, 60, 90, 120, and 150 minutes travel time at administrative level 1 for Liberia.

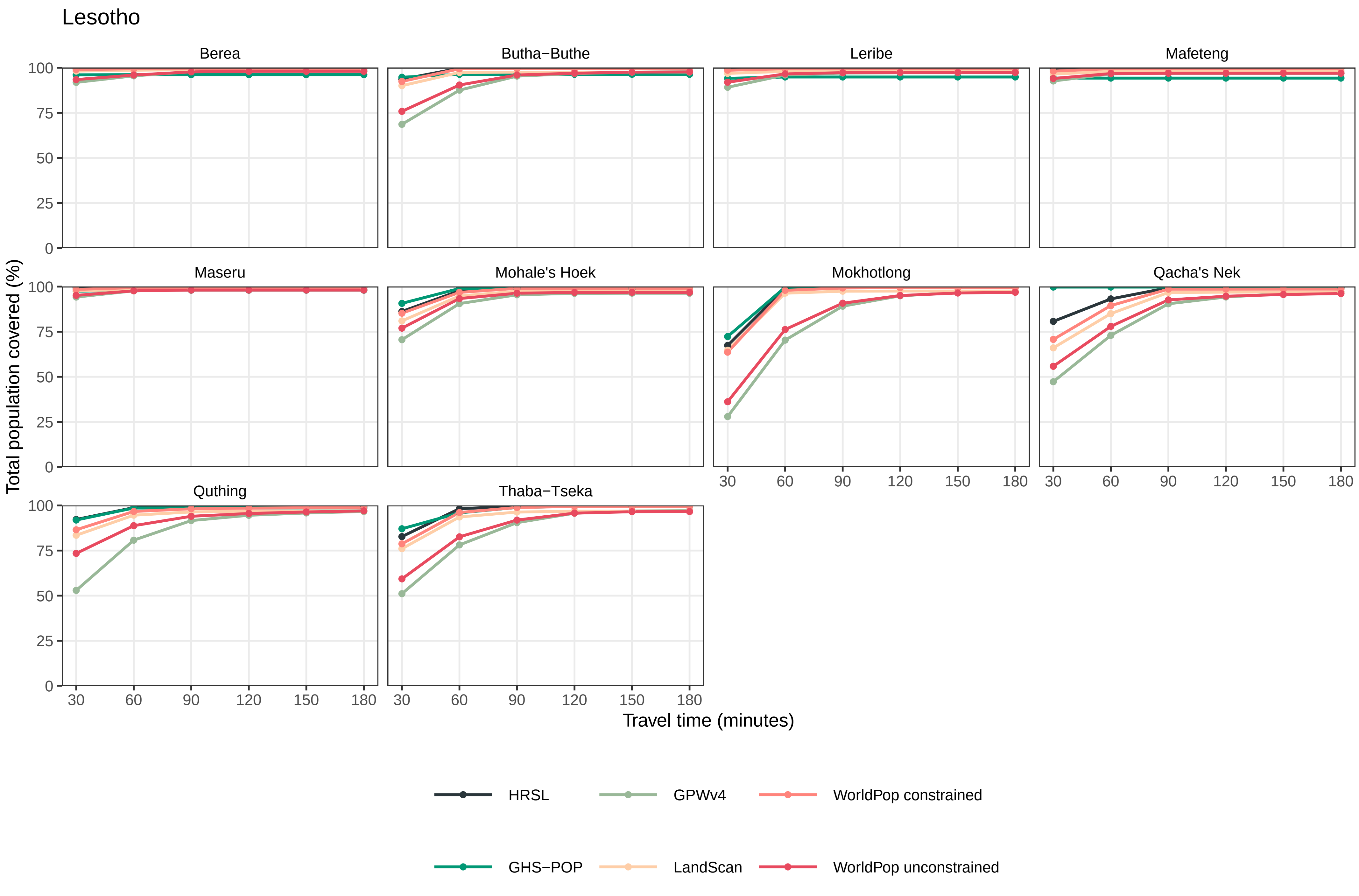

Supplementary Figure 26 − Accessibility coverage at 30, 60, 90, 120, and 150 minutes travel time at administrative level 1 for Lesotho.

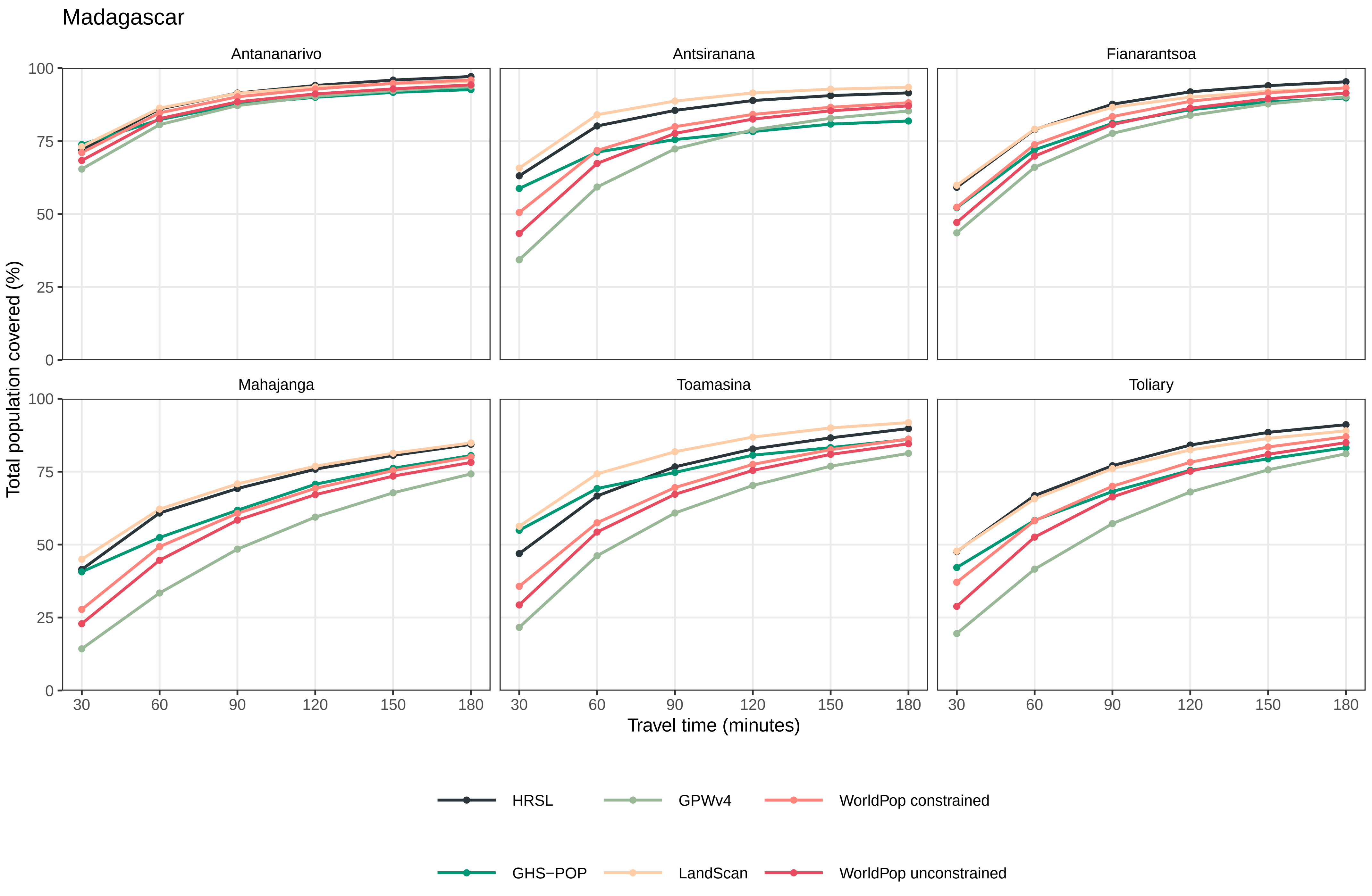

Supplementary Figure 27 − Accessibility coverage at 30, 60, 90, 120, and 150 minutes travel time at administrative level 1 for Madagascar.

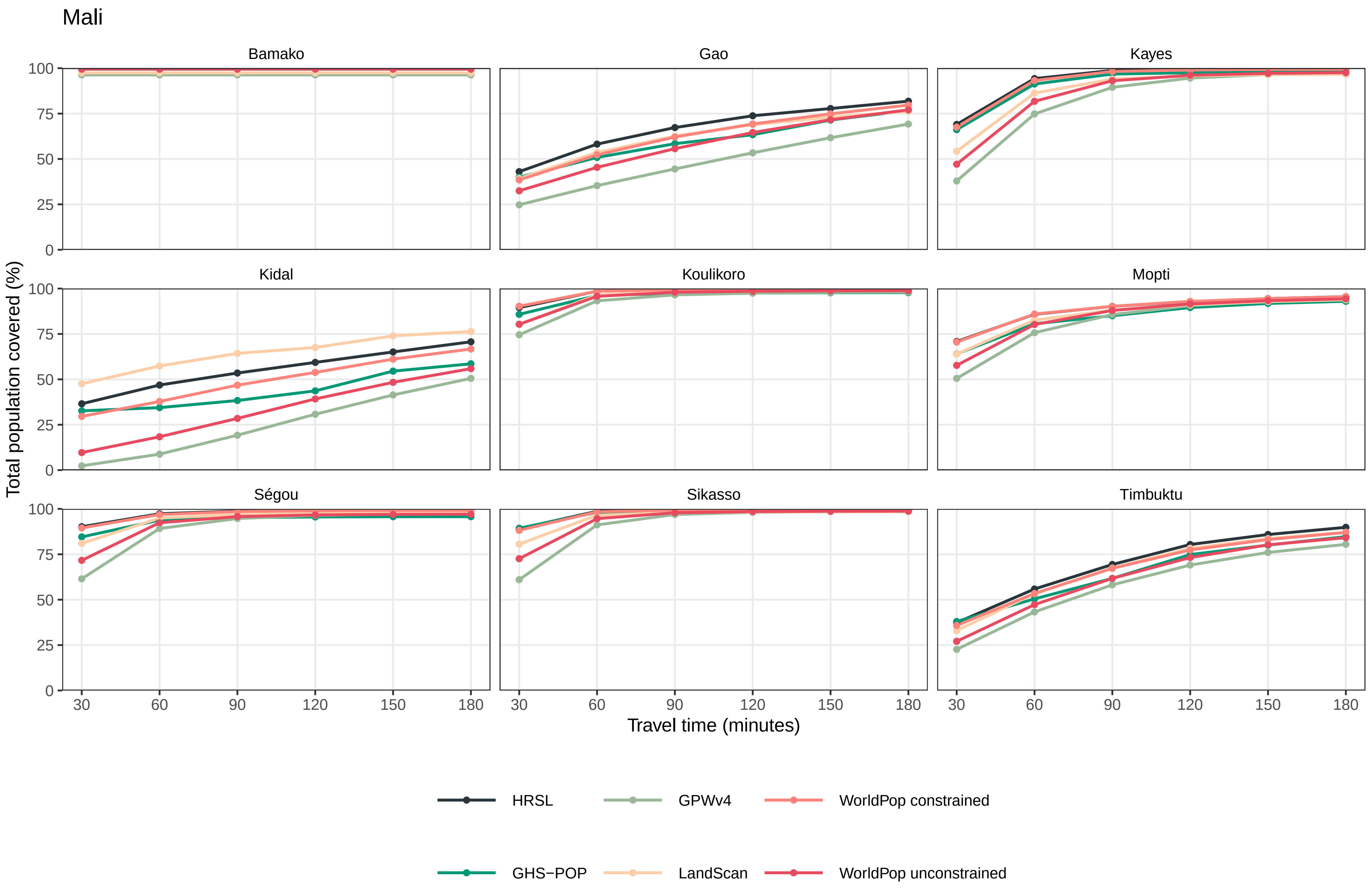

Supplementary Figure 28 − Accessibility coverage at 30, 60, 90, 120, and 150 minutes travel time at administrative level 1 for Mali.

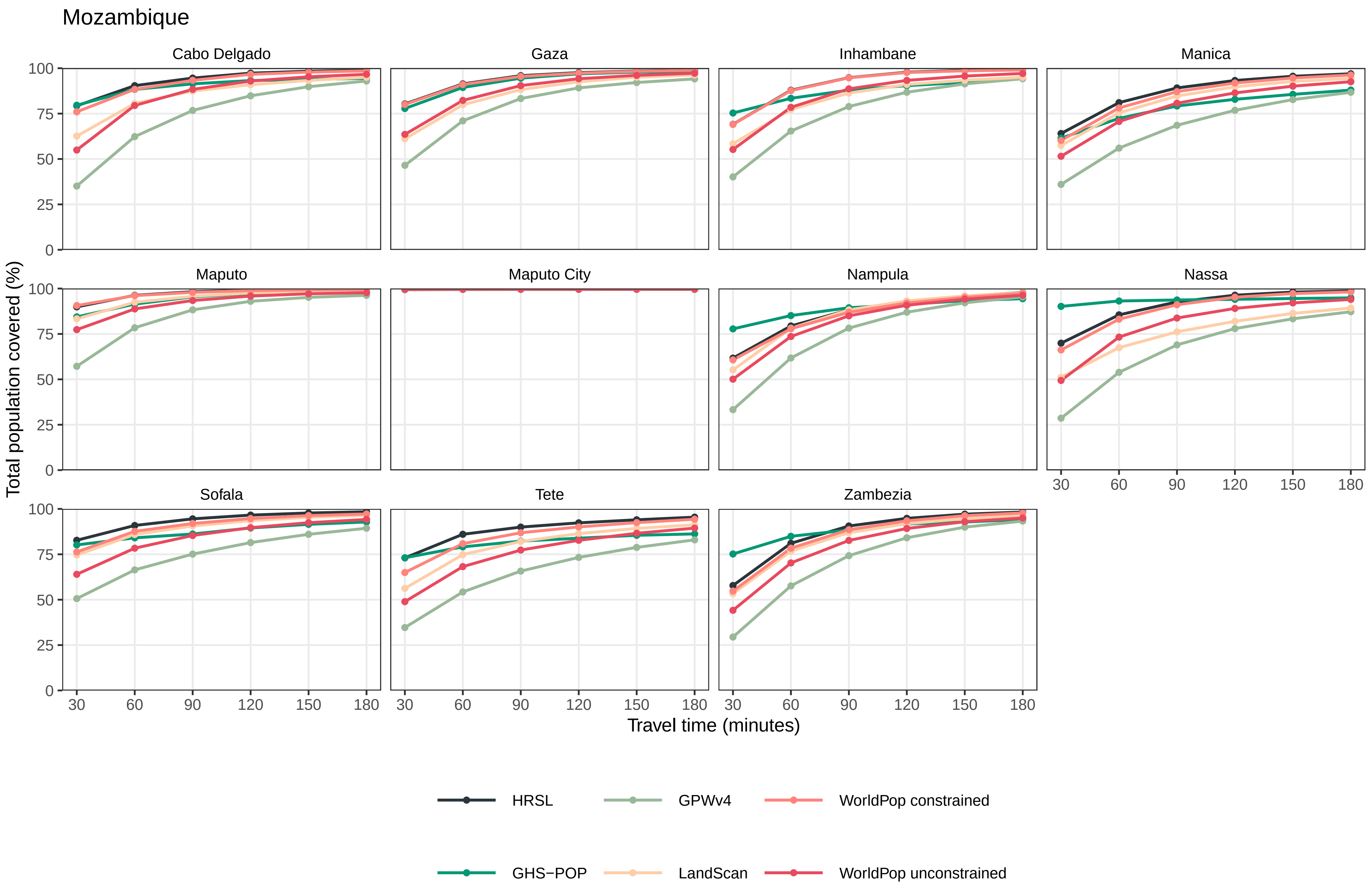

Supplementary Figure 29 − Accessibility coverage at 30, 60, 90, 120, and 150 minutes travel time at administrative level 1 for Mozambique.

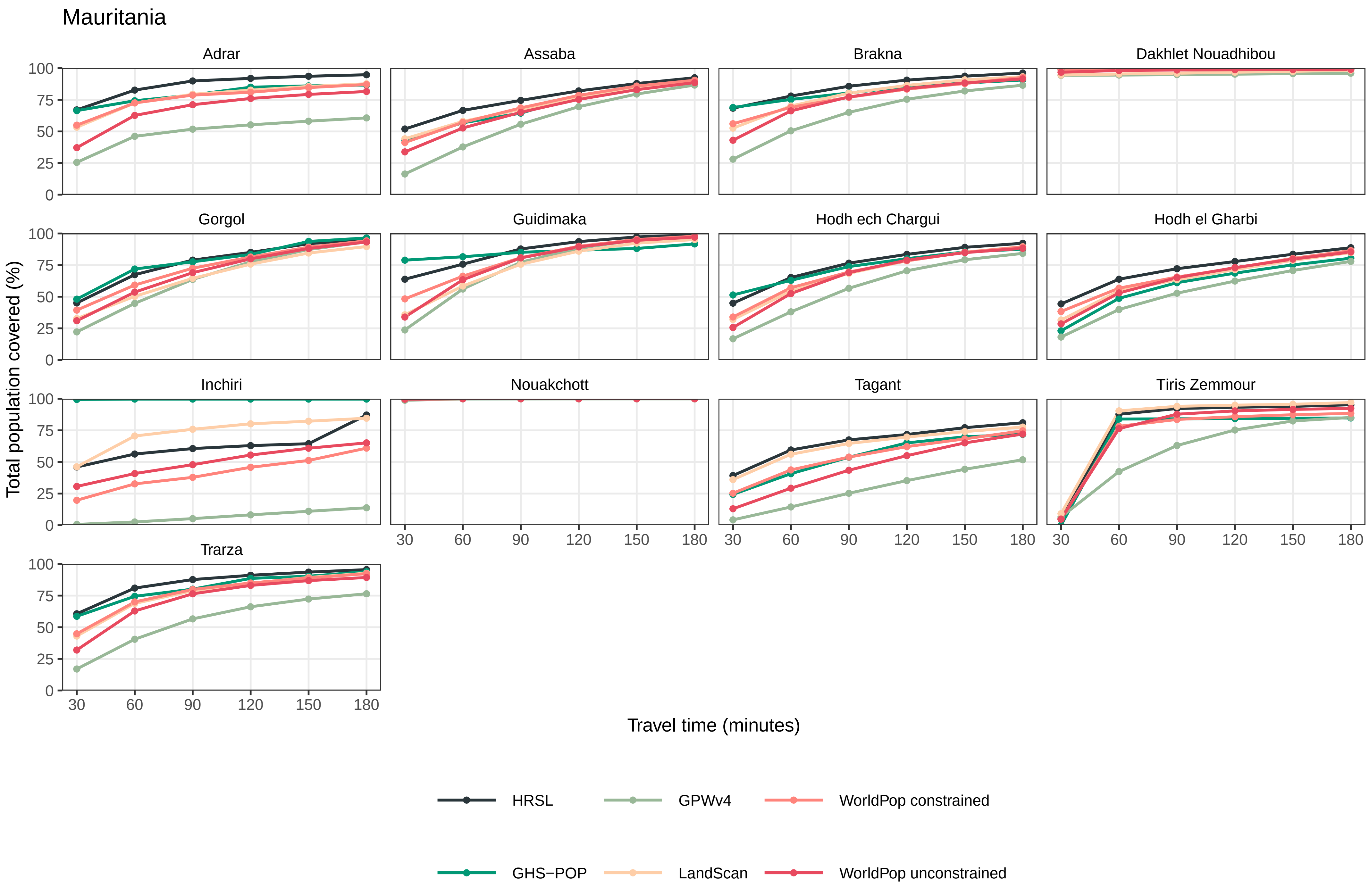

Supplementary Figure 30 − Accessibility coverage at 30, 60, 90, 120, and 150 minutes travel time at administrative level 1 for Mauritania.

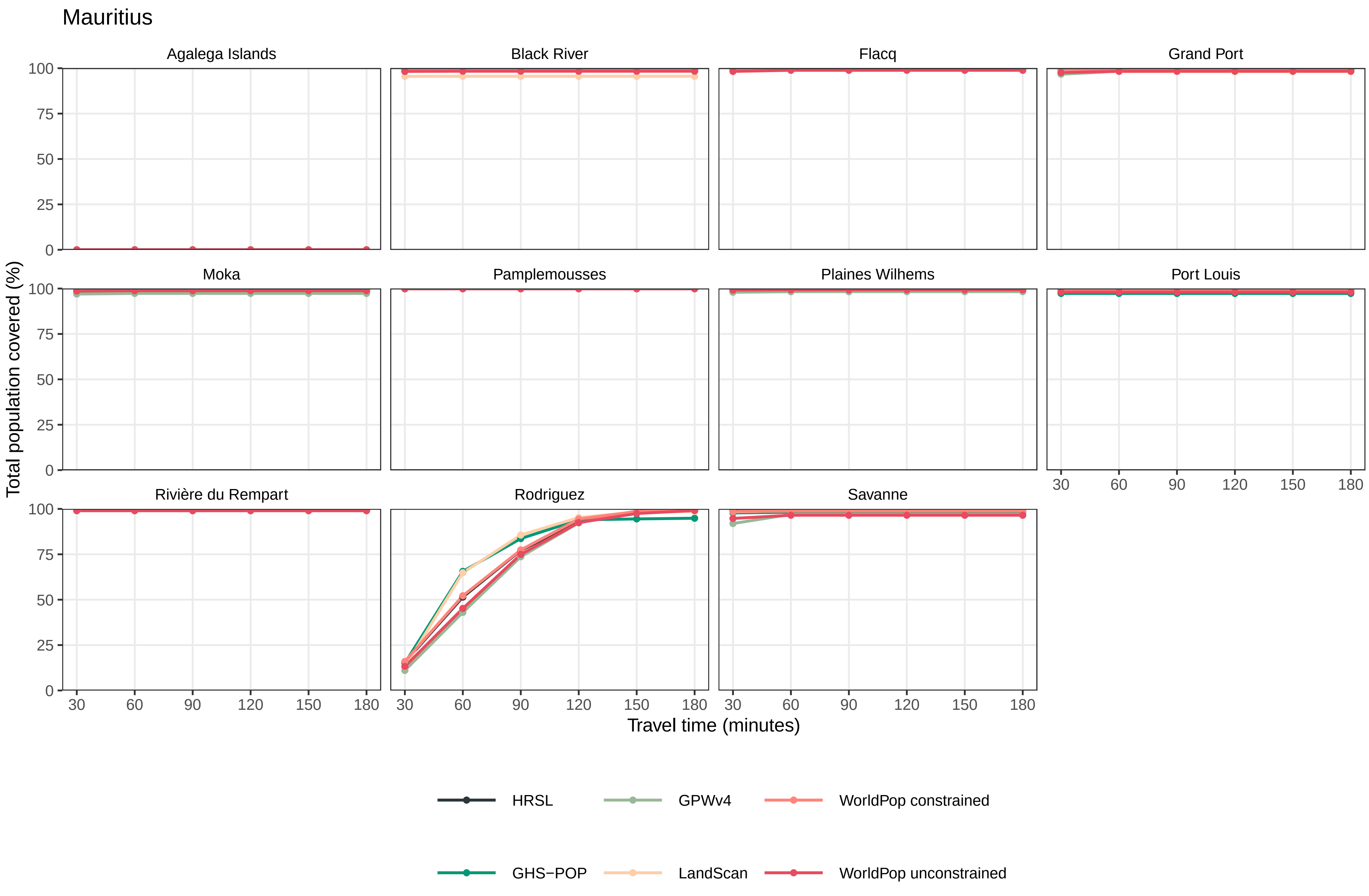

Supplementary Figure 31 − Accessibility coverage at 30, 60, 90, 120, and 150 minutes travel time at administrative level 1 for Mauritius.

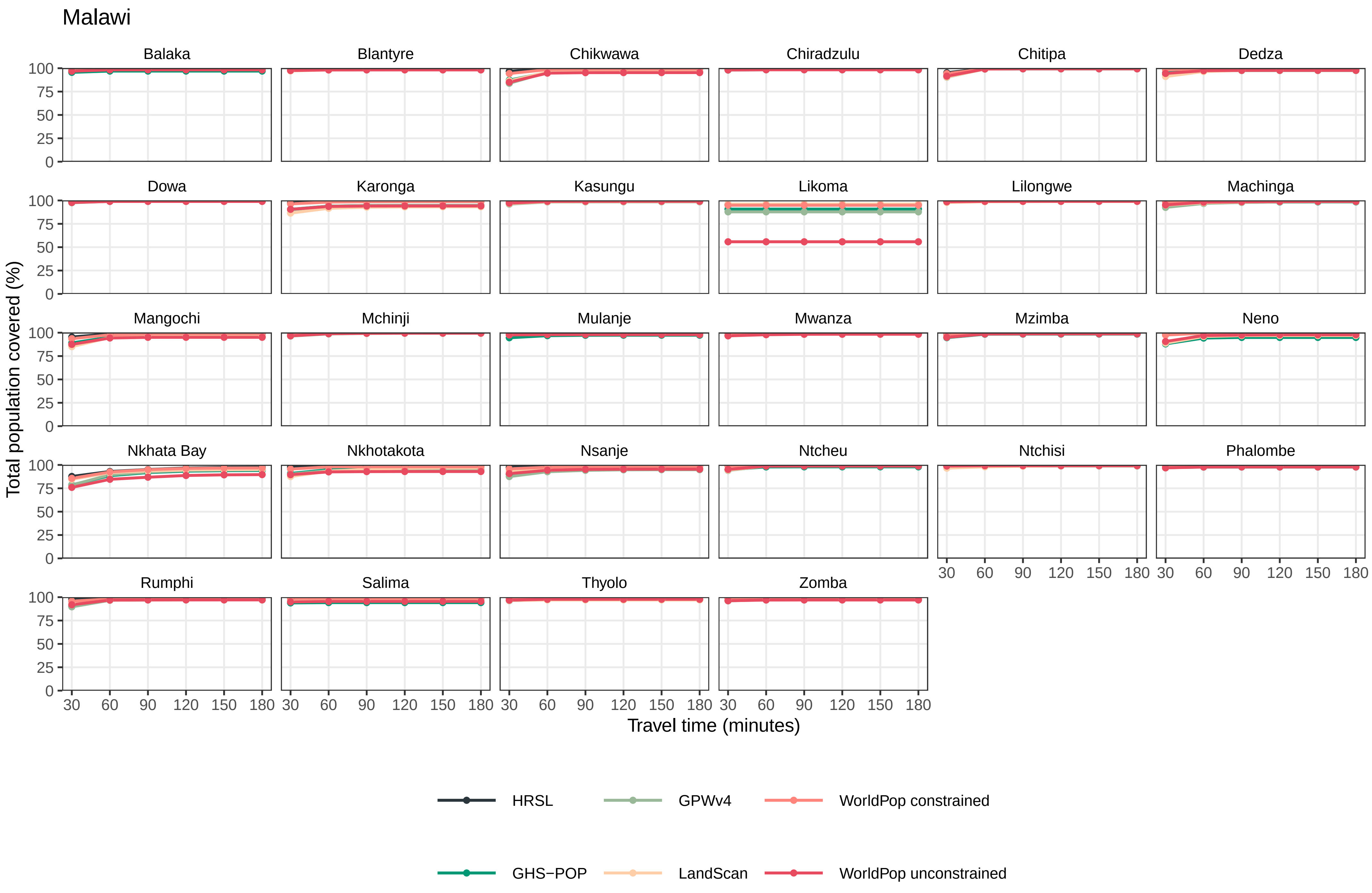

Supplementary Figure 32 − Accessibility coverage at 30, 60, 90, 120, and 150 minutes travel time at administrative level 1 for Malawi.

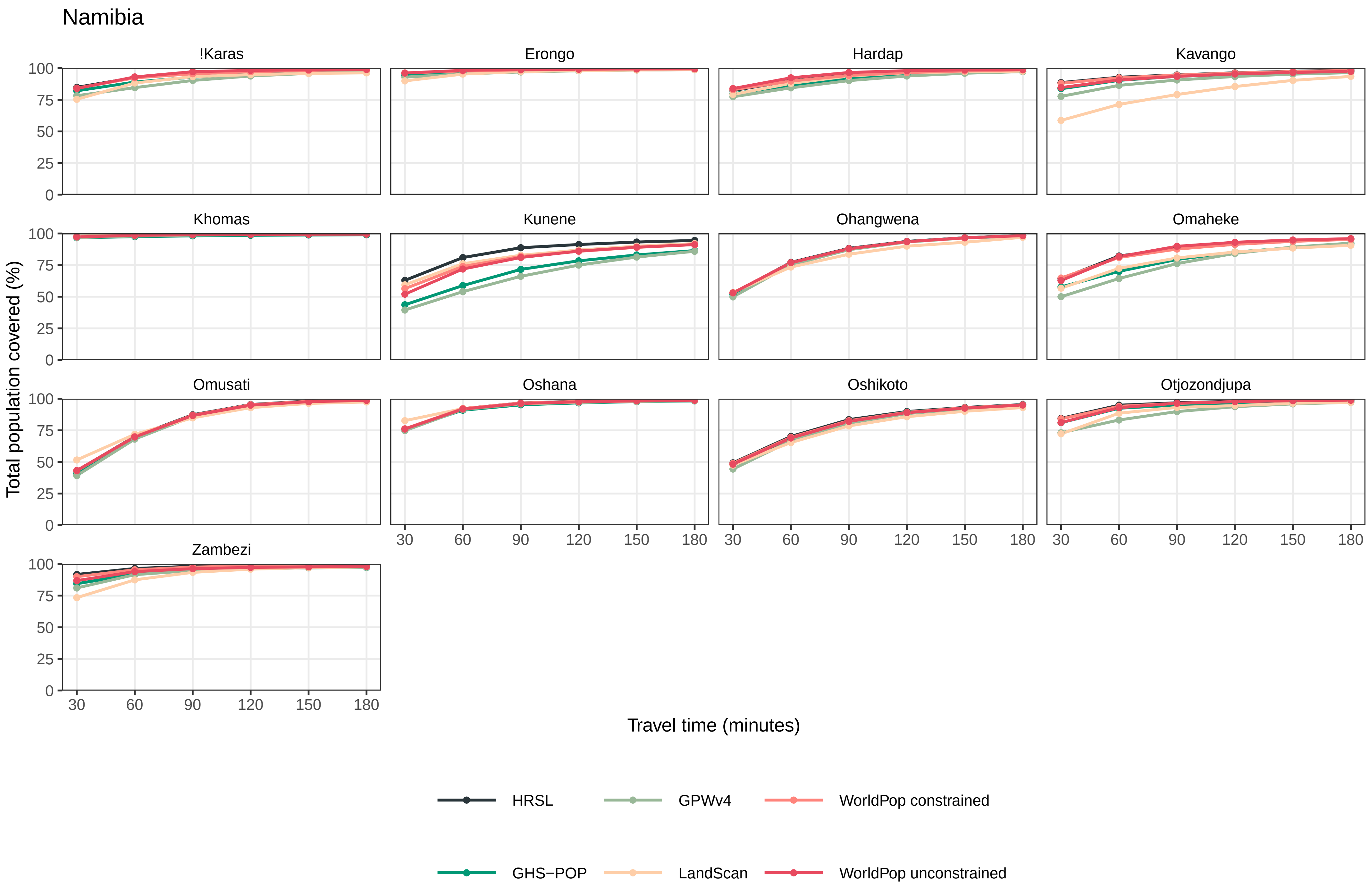

Supplementary Figure 33 − Accessibility coverage at 30, 60, 90, 120, and 150 minutes travel time at administrative level 1 for Namibia.

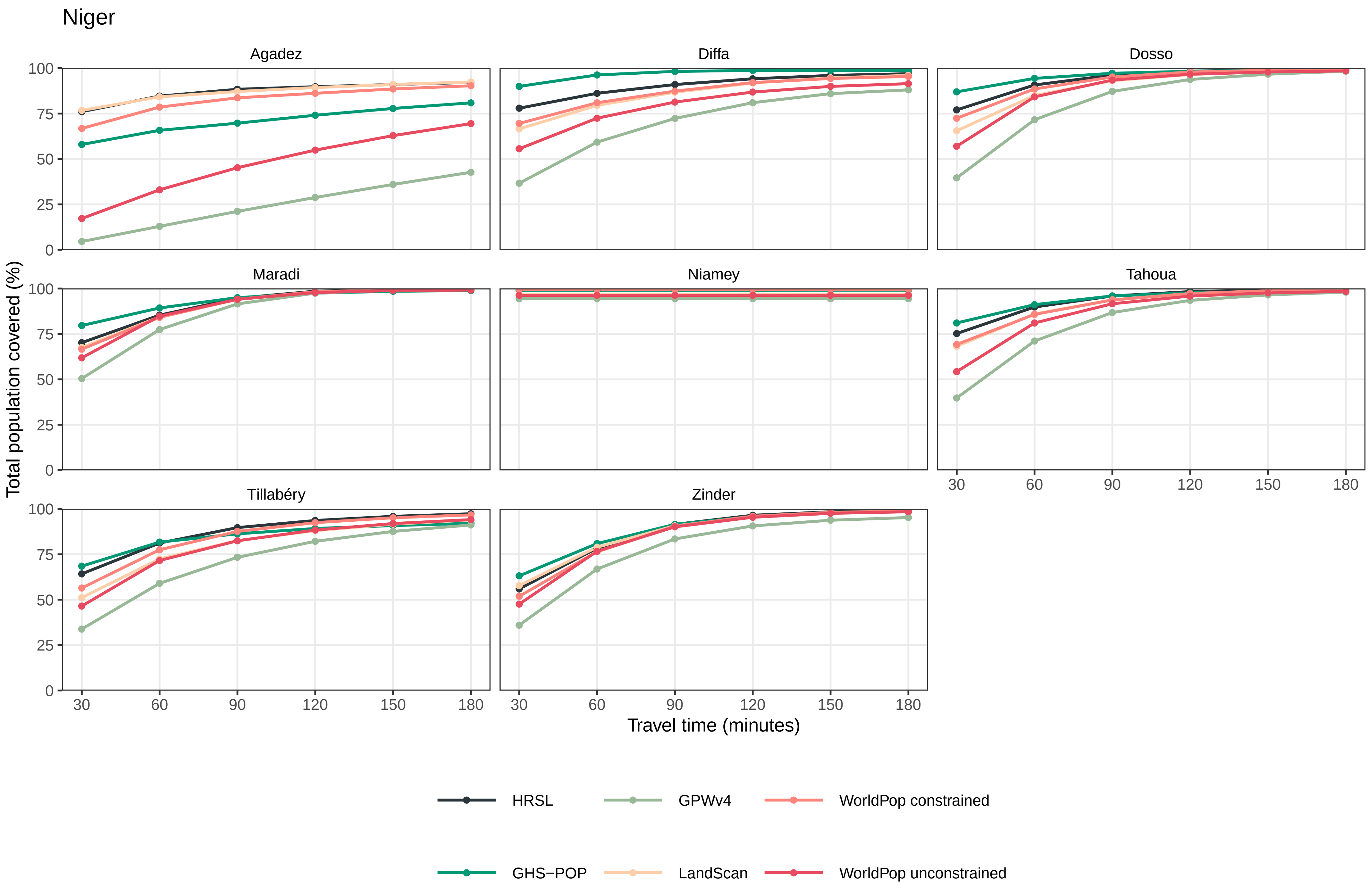

Supplementary Figure 34 − Accessibility coverage at 30, 60, 90, 120, and 150 minutes travel time at administrative level 1 for Niger.

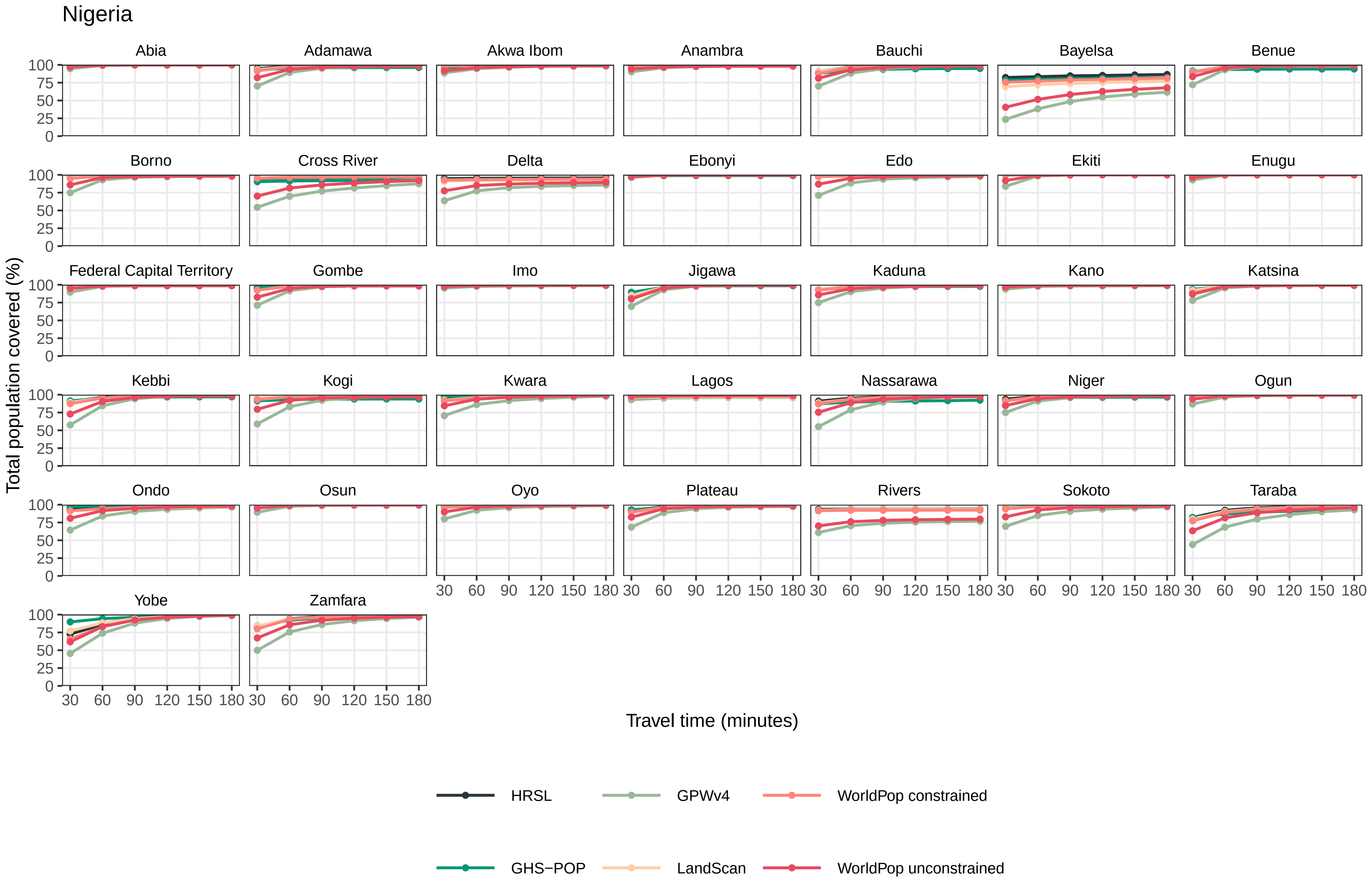

Supplementary Figure 35 − Accessibility coverage at 30, 60, 90, 120, and 150 minutes travel time at administrative level 1 for Nigeria.

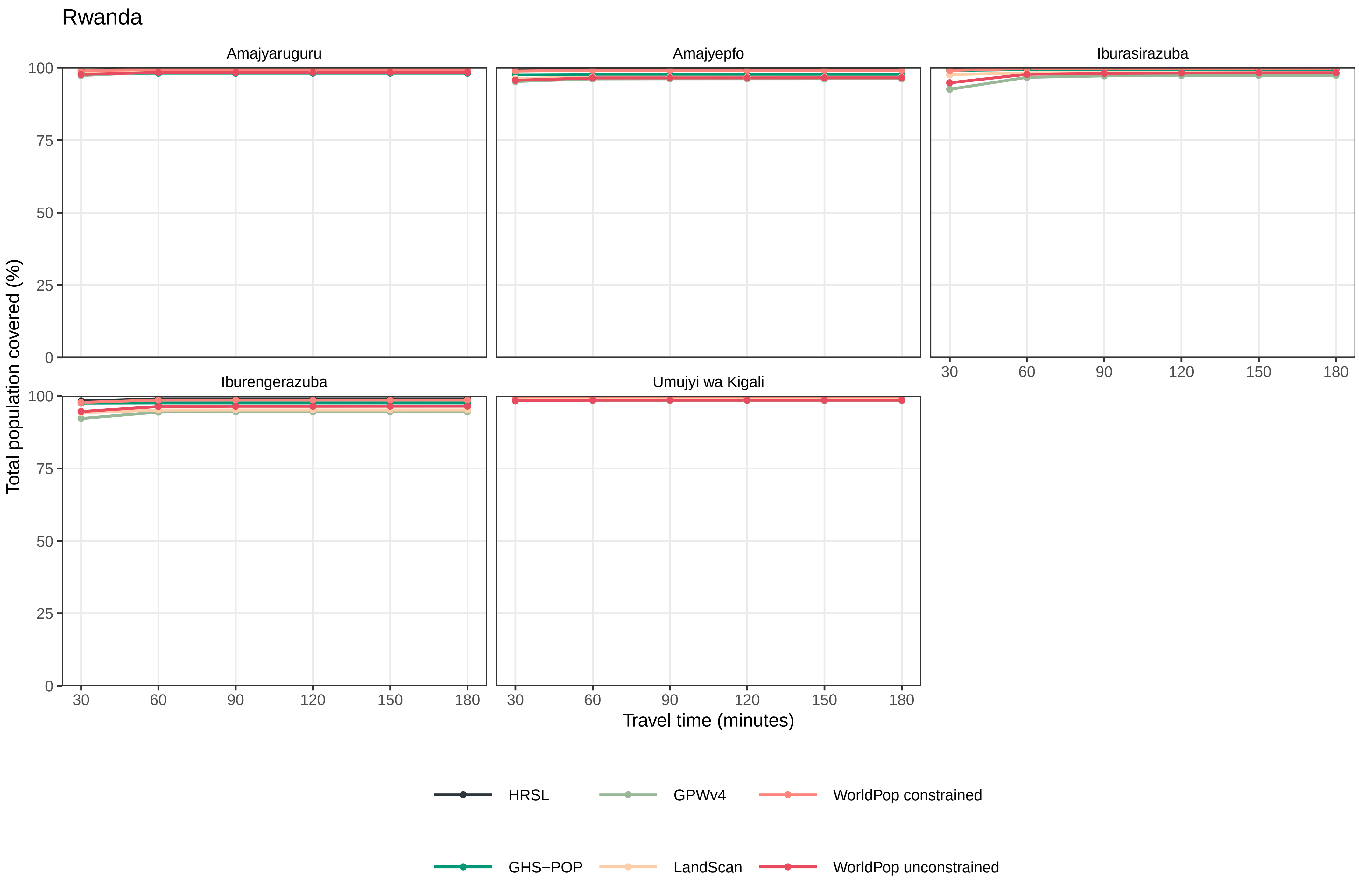

Supplementary Figure 36 − Accessibility coverage at 30, 60, 90, 120, and 150 minutes travel time at administrative level 1 for Rwanda.

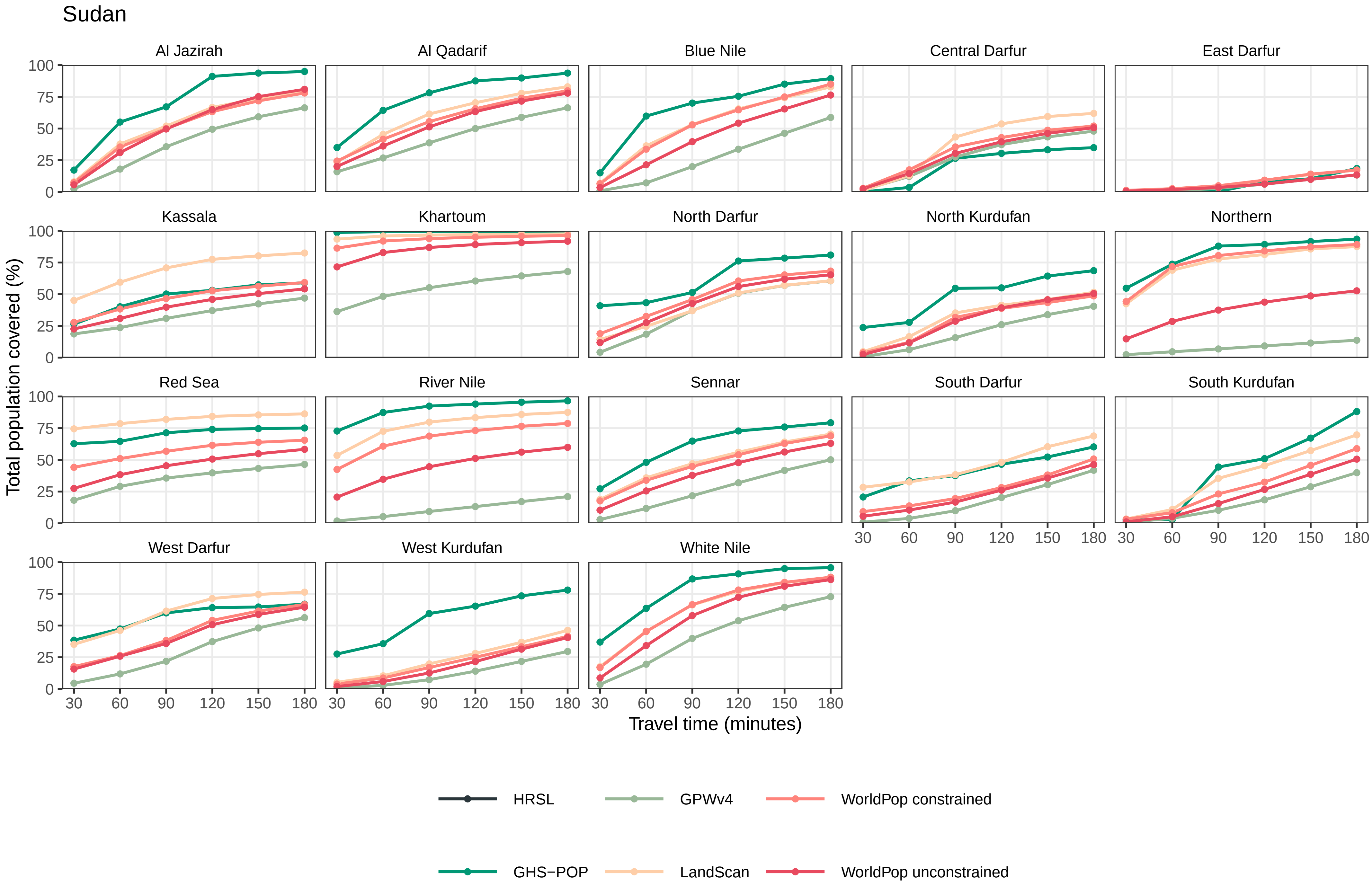

Supplementary Figure 37 − Accessibility coverage at 30, 60, 90, 120, and 150 minutes travel time at administrative level 1 for Sudan.

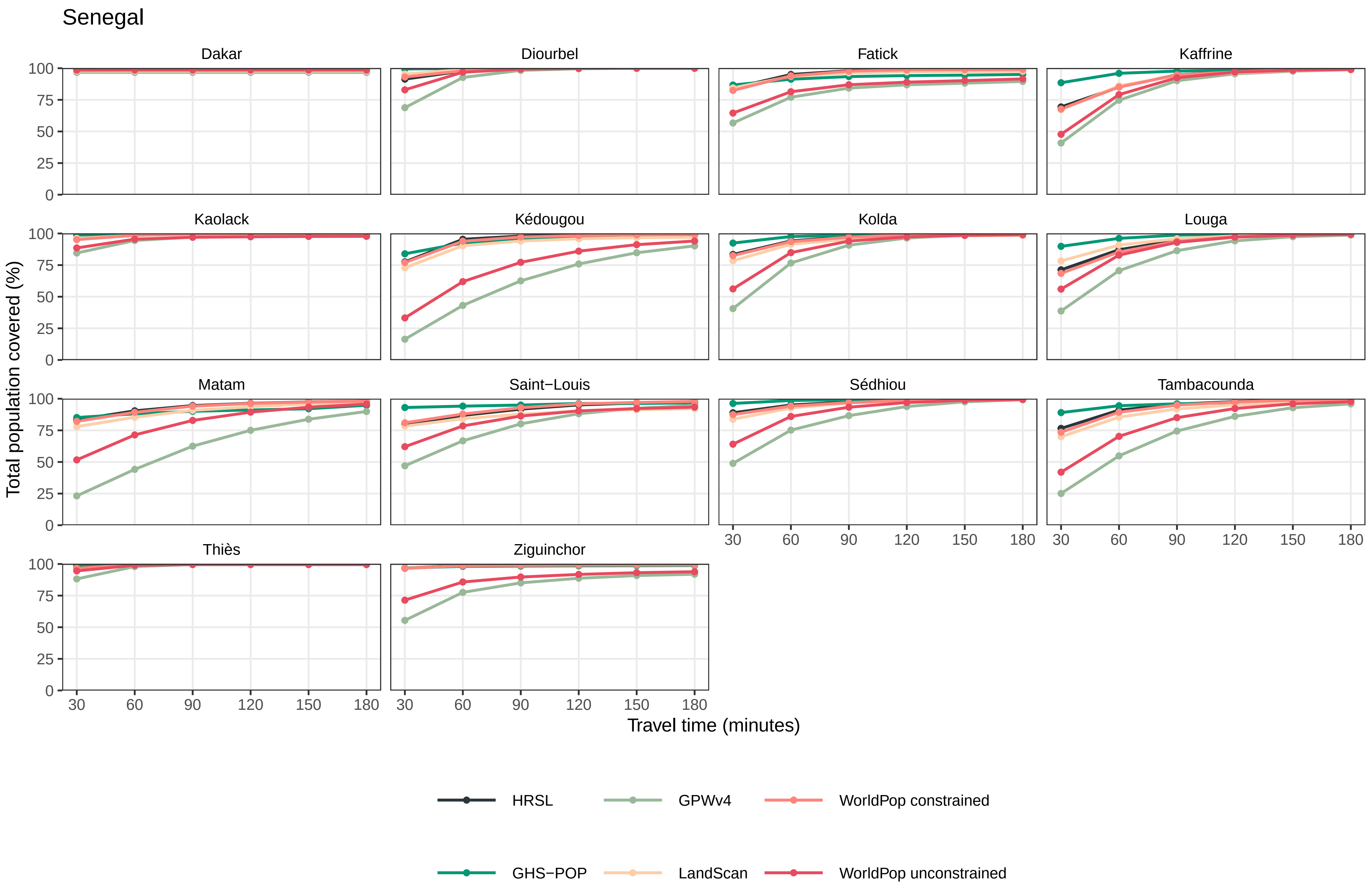

Supplementary Figure 38 − Accessibility coverage at 30, 60, 90, 120, and 150 minutes travel time at administrative level 1 for Senegal.

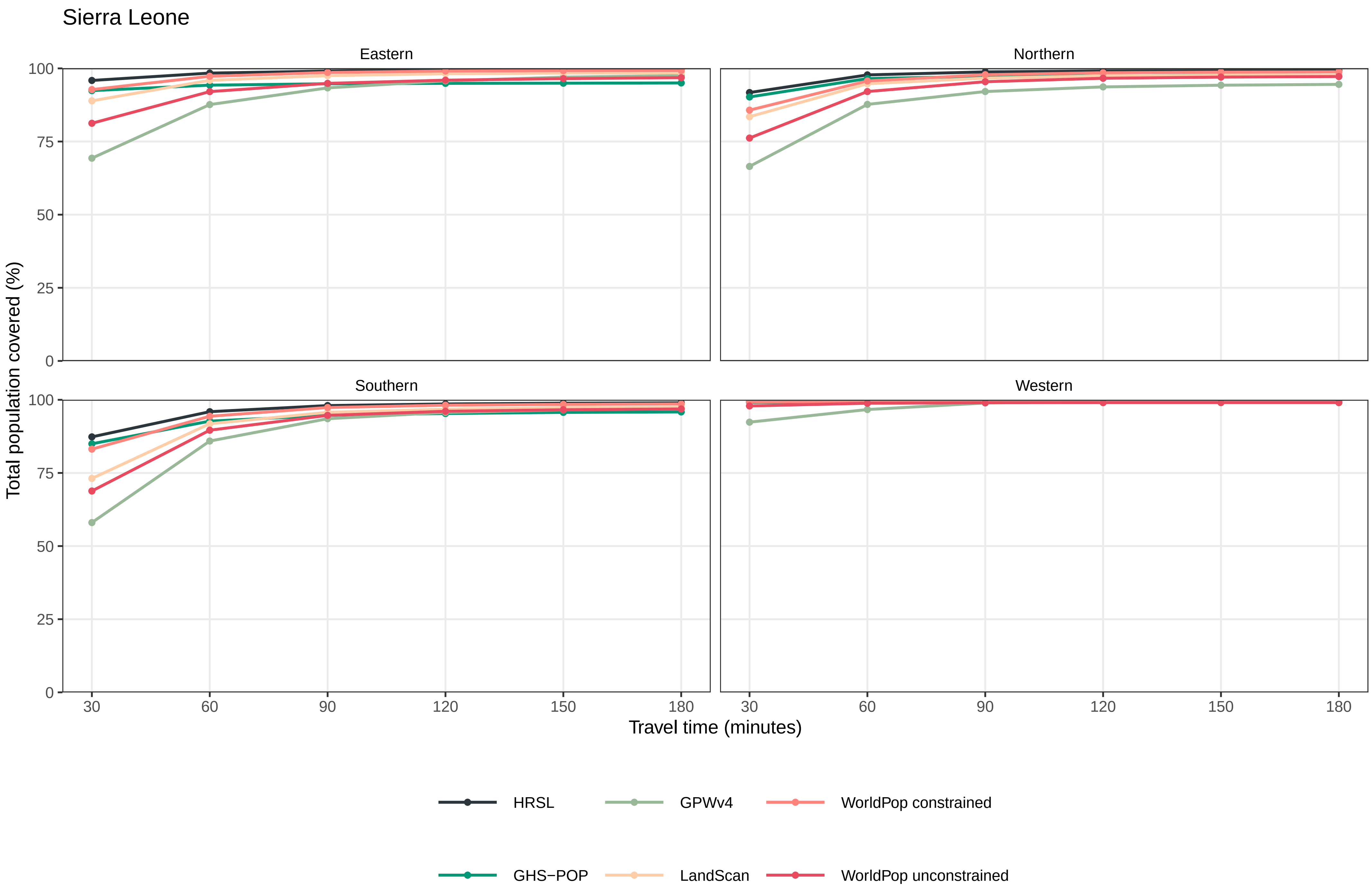

Supplementary Figure 39 − Accessibility coverage at 30, 60, 90, 120, and 150 minutes travel time at administrative level 1 for Sierra Leone.

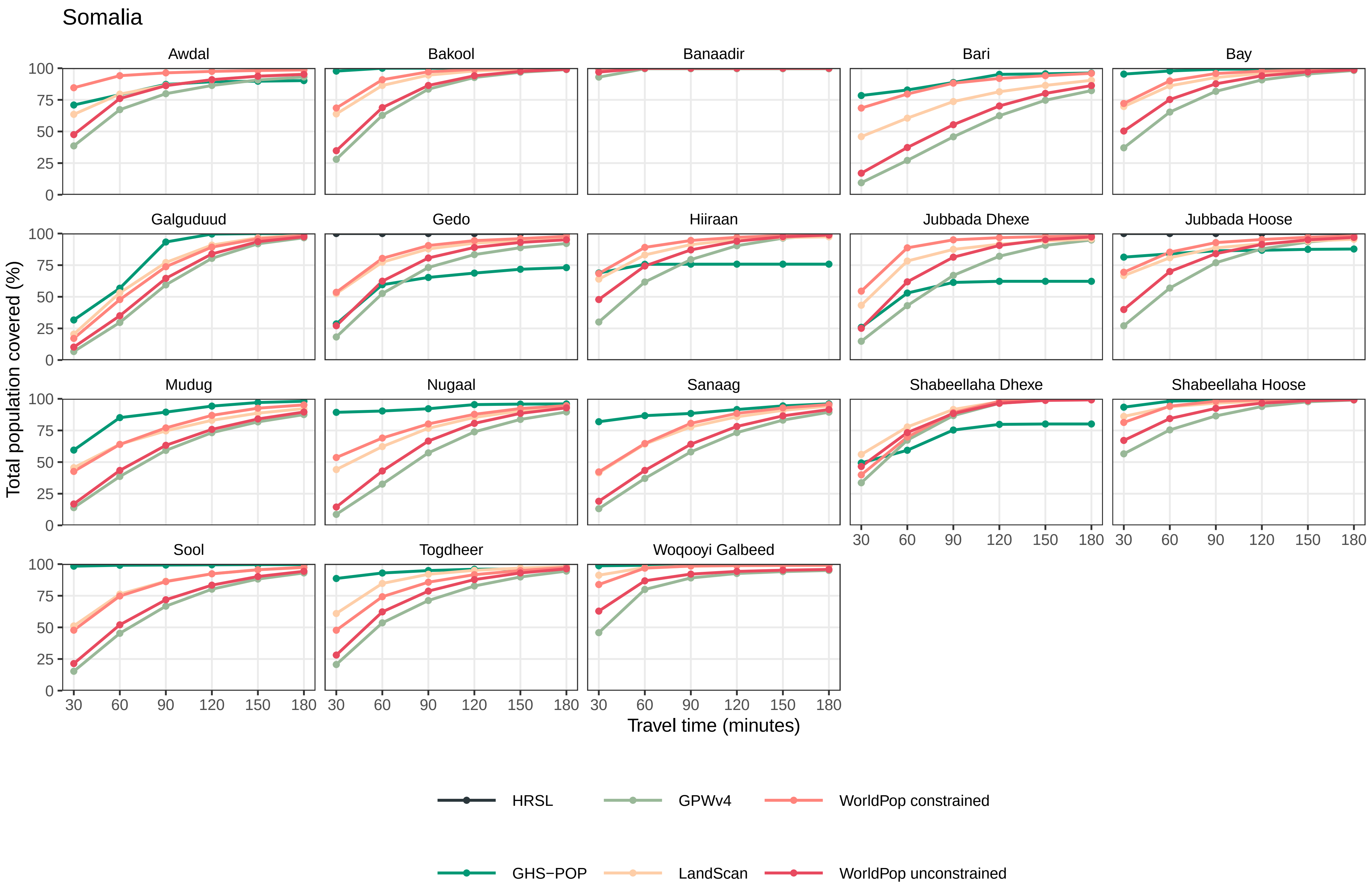

Supplementary Figure 40 − Accessibility coverage at 30, 60, 90, 120, and 150 minutes travel time at administrative level 1 for Somalia.

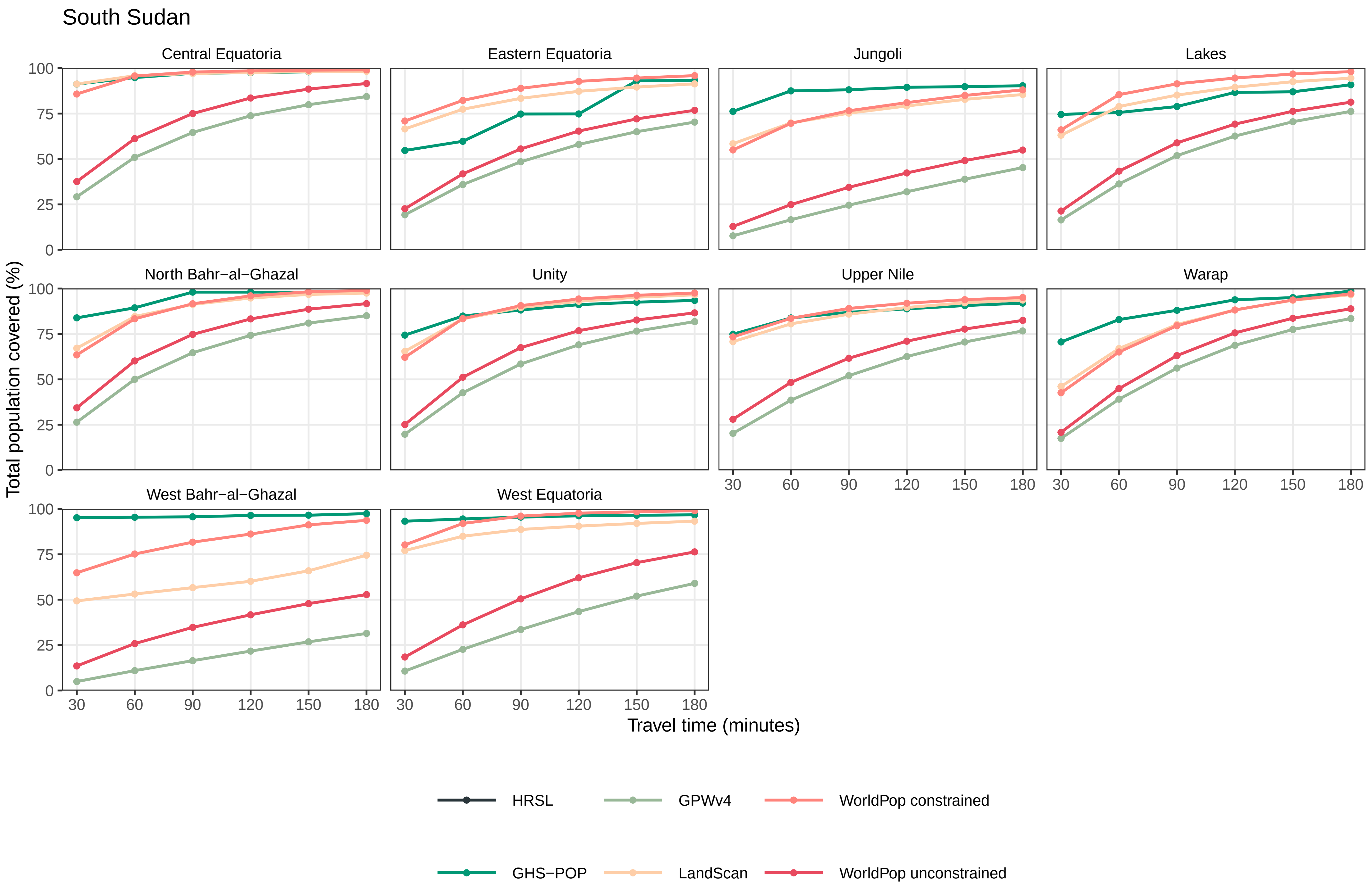

Supplementary Figure 41 − Accessibility coverage at 30, 60, 90, 120, and 150 minutes travel time at administrative level 1 for South Sudan.

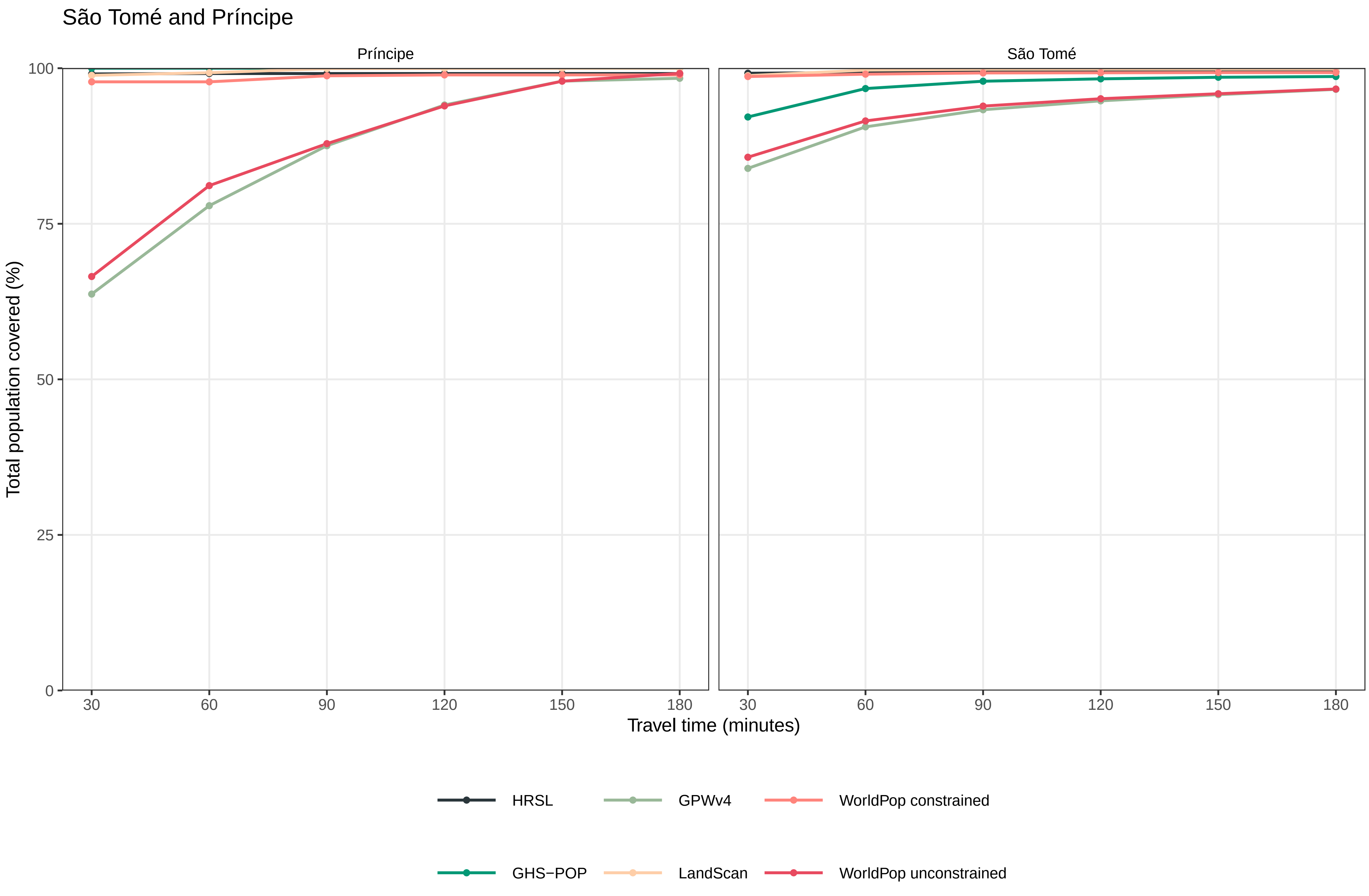

Supplementary Figure 42 − Accessibility coverage at 30, 60, 90, 120, and 150 minutes travel time at administrative level 1 for São Tomé and Príncipe.

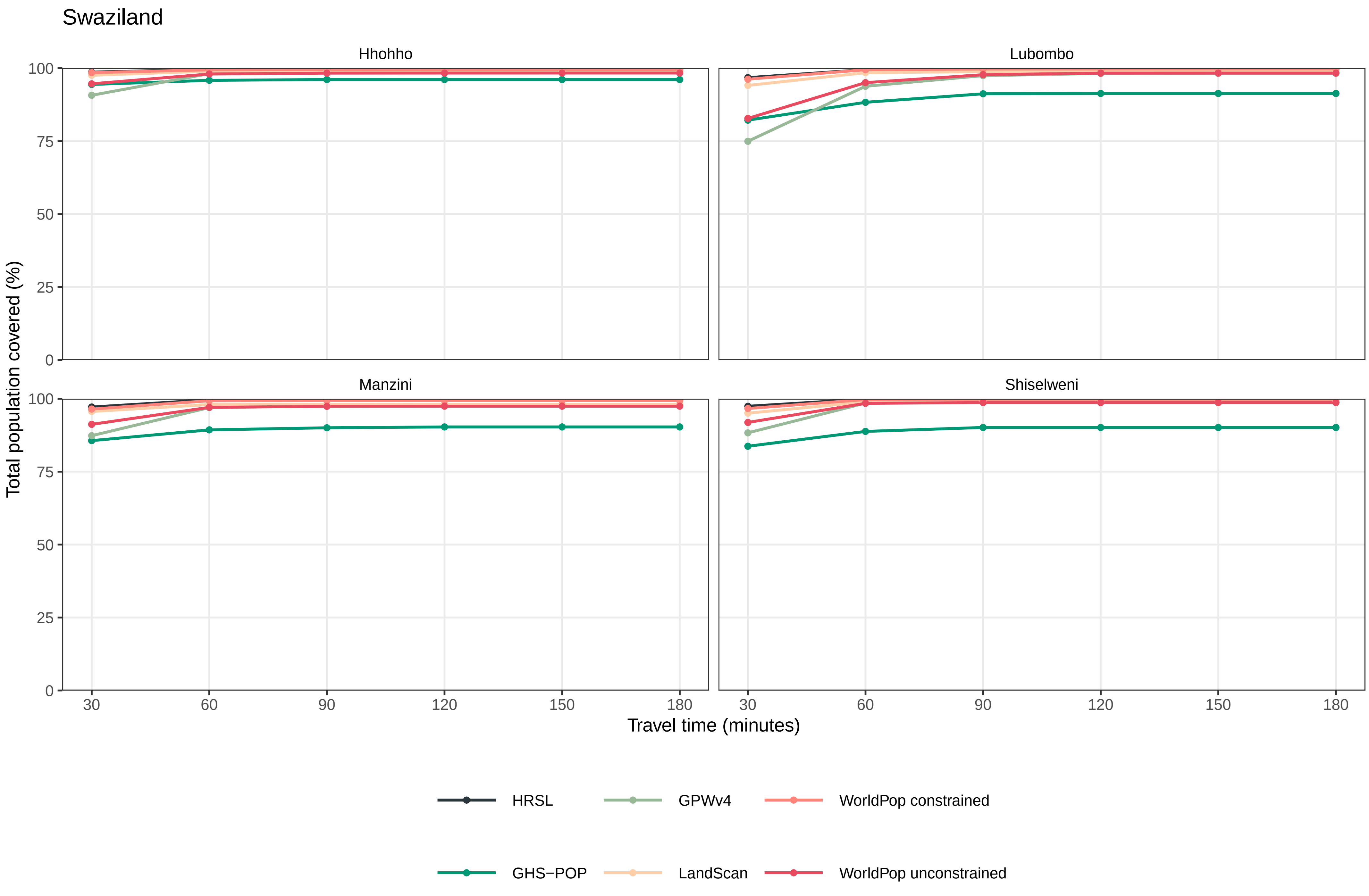

Supplementary Figure 43 − Accessibility coverage at 30, 60, 90, 120, and 150 minutes travel time at administrative level 1 for Swaziland.

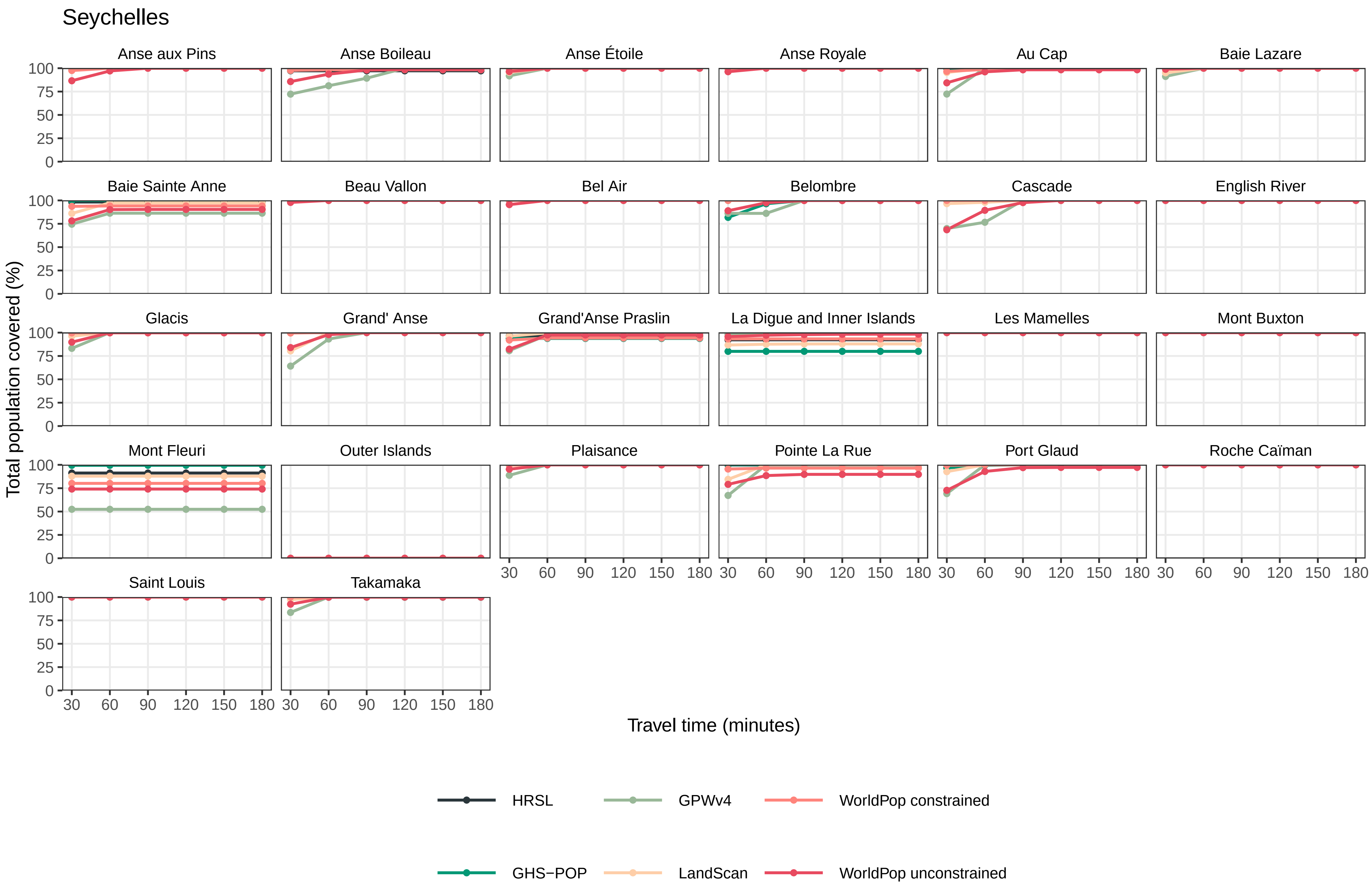

Supplementary Figure 44 − Accessibility coverage at 30, 60, 90, 120, and 150 minutes travel time at administrative level 1 for Seychelles.

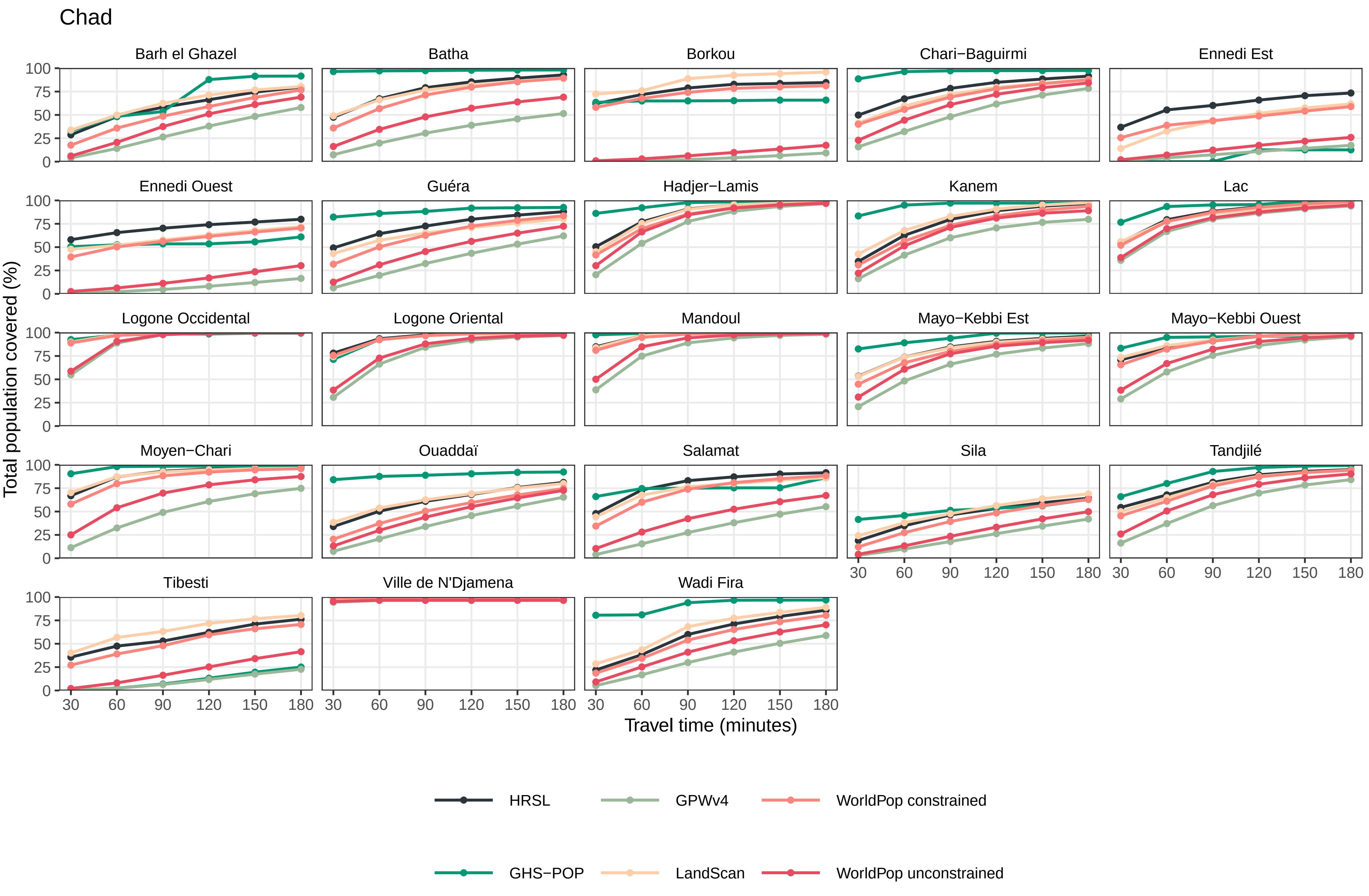

Supplementary Figure 45 − Accessibility coverage at 30, 60, 90, 120, and 150 minutes travel time at administrative level 1 for Chad.

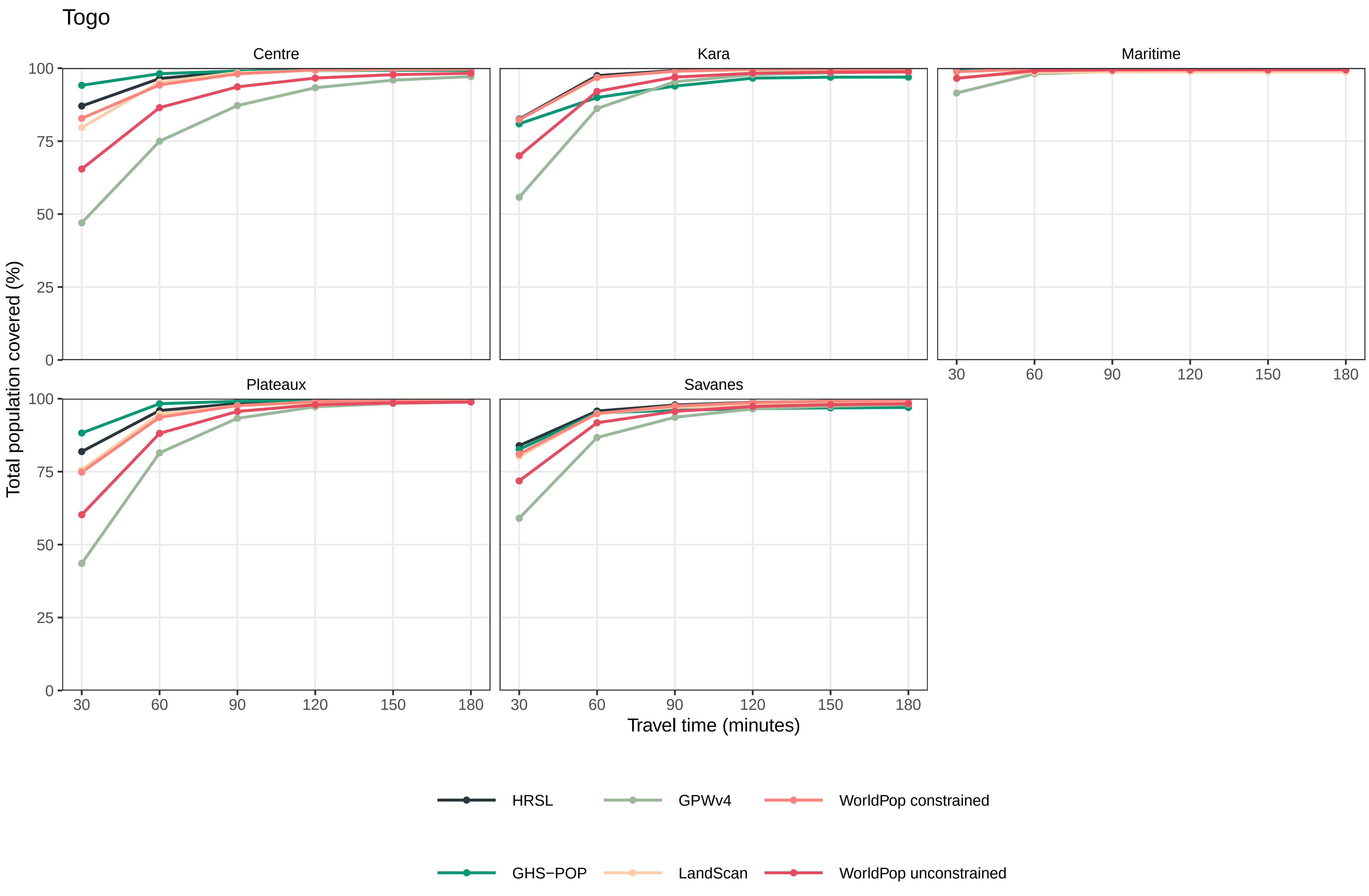

Supplementary Figure 46 − Accessibility coverage at 30, 60, 90, 120, and 150 minutes travel time at administrative level 1 for Togo.

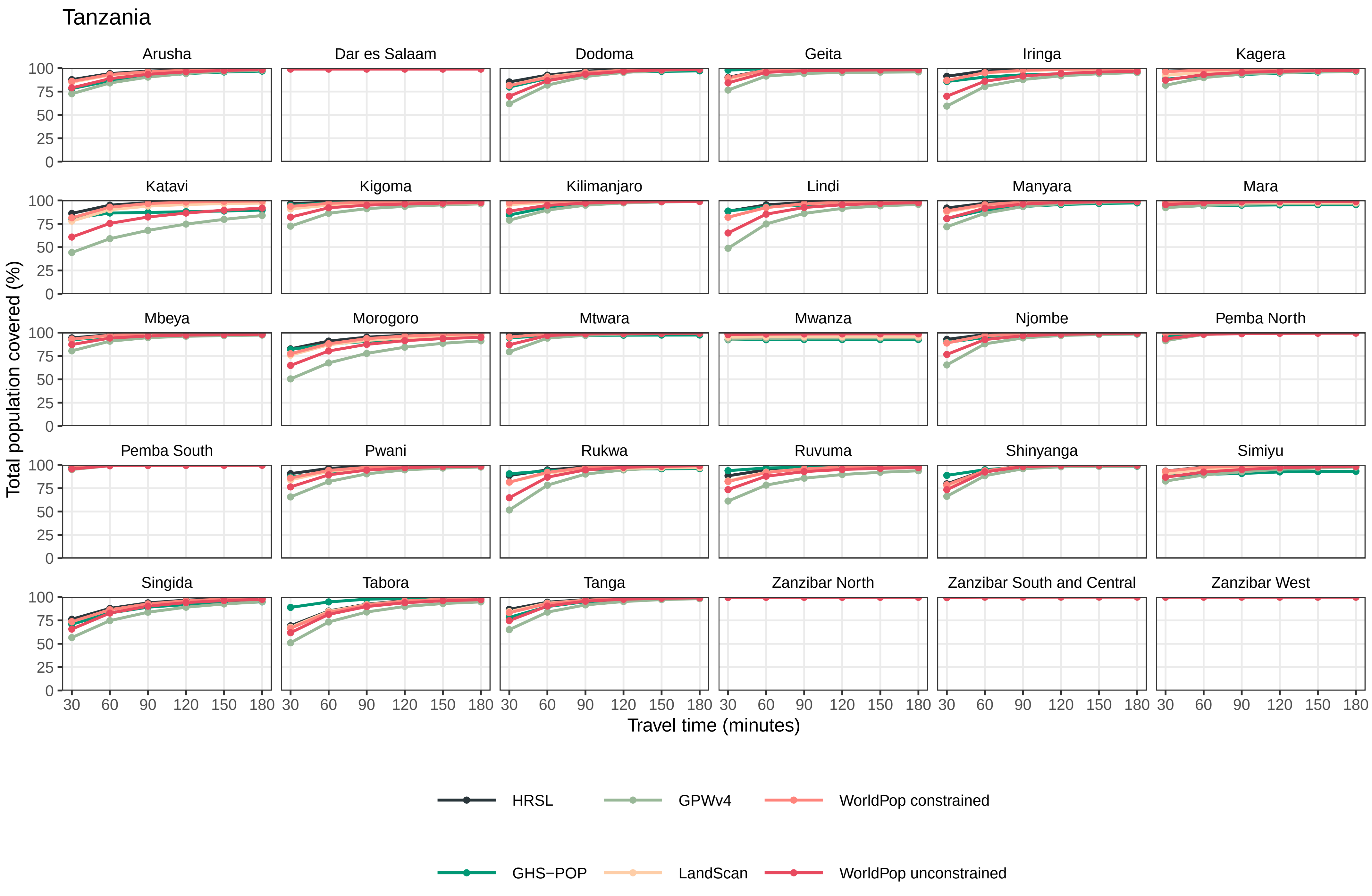

Supplementary Figure 47 − Accessibility coverage at 30, 60, 90, 120, and 150 minutes travel time at administrative level 1 for Tanzania.

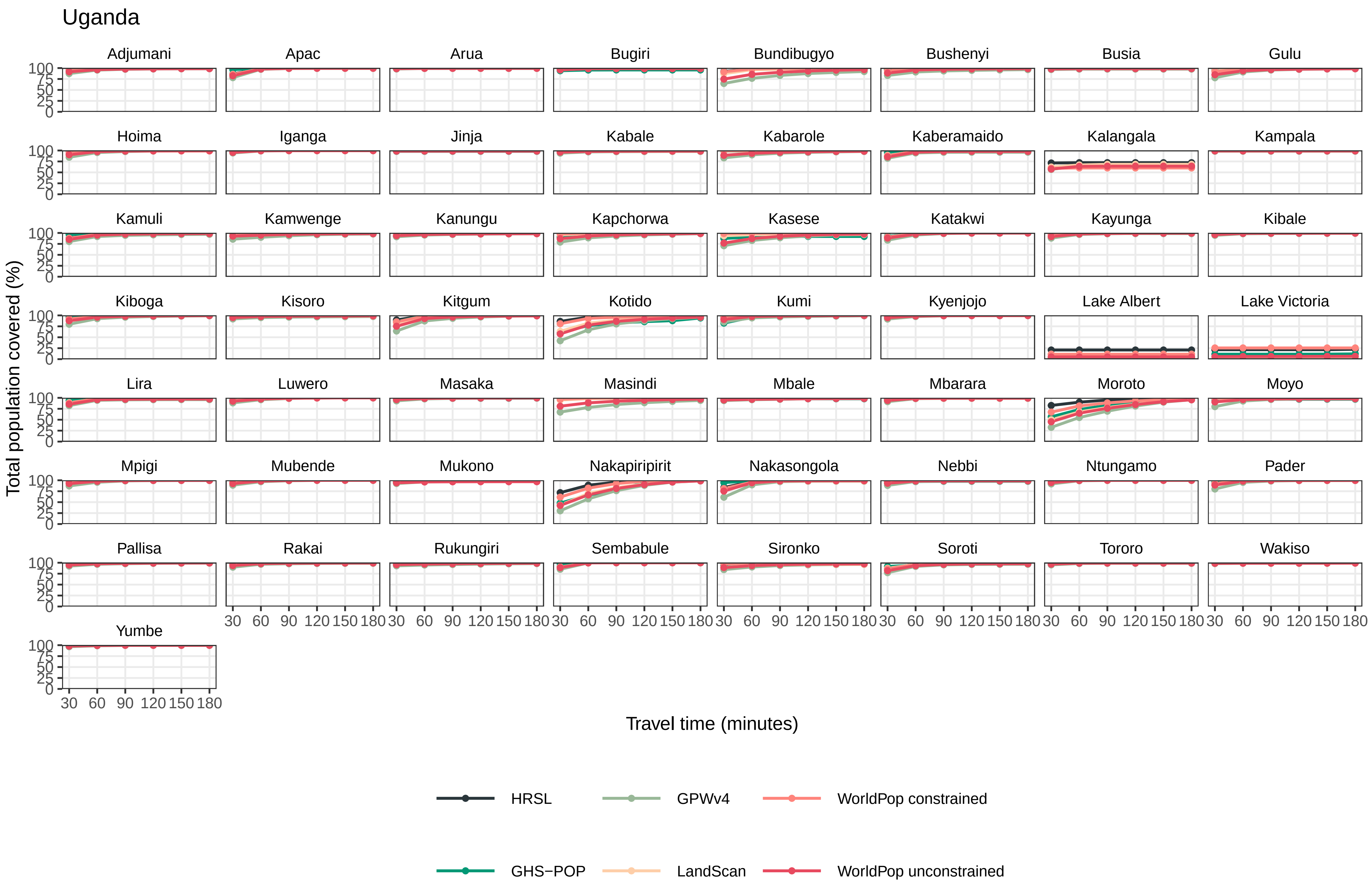

Supplementary Figure 48 − Accessibility coverage at 30, 60, 90, 120, and 150 minutes travel time at administrative level 1 for Uganda.

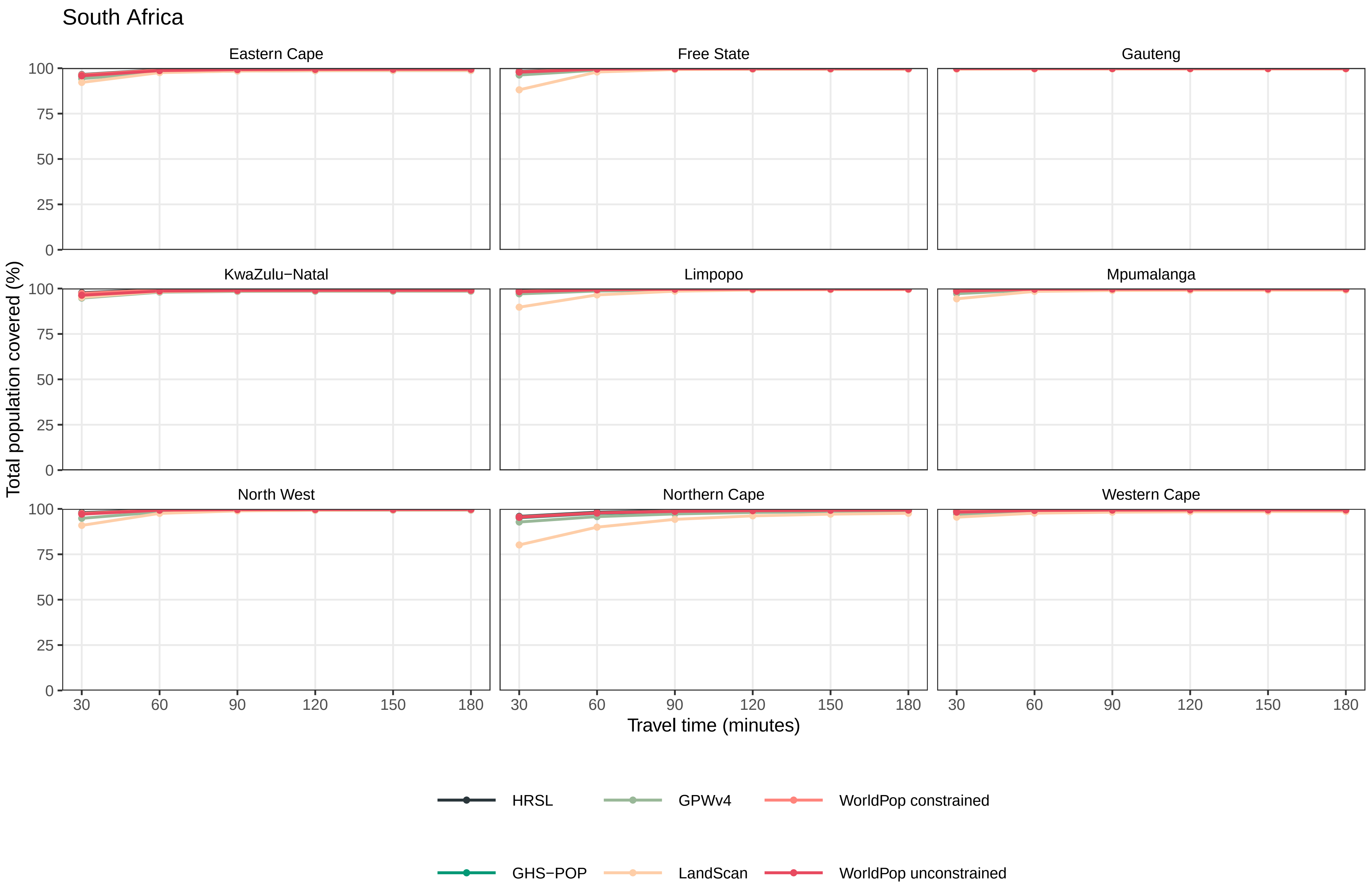

Supplementary Figure 49 − Accessibility coverage at 30, 60, 90, 120, and 150 minutes travel time at administrative level 1 for South Africa.

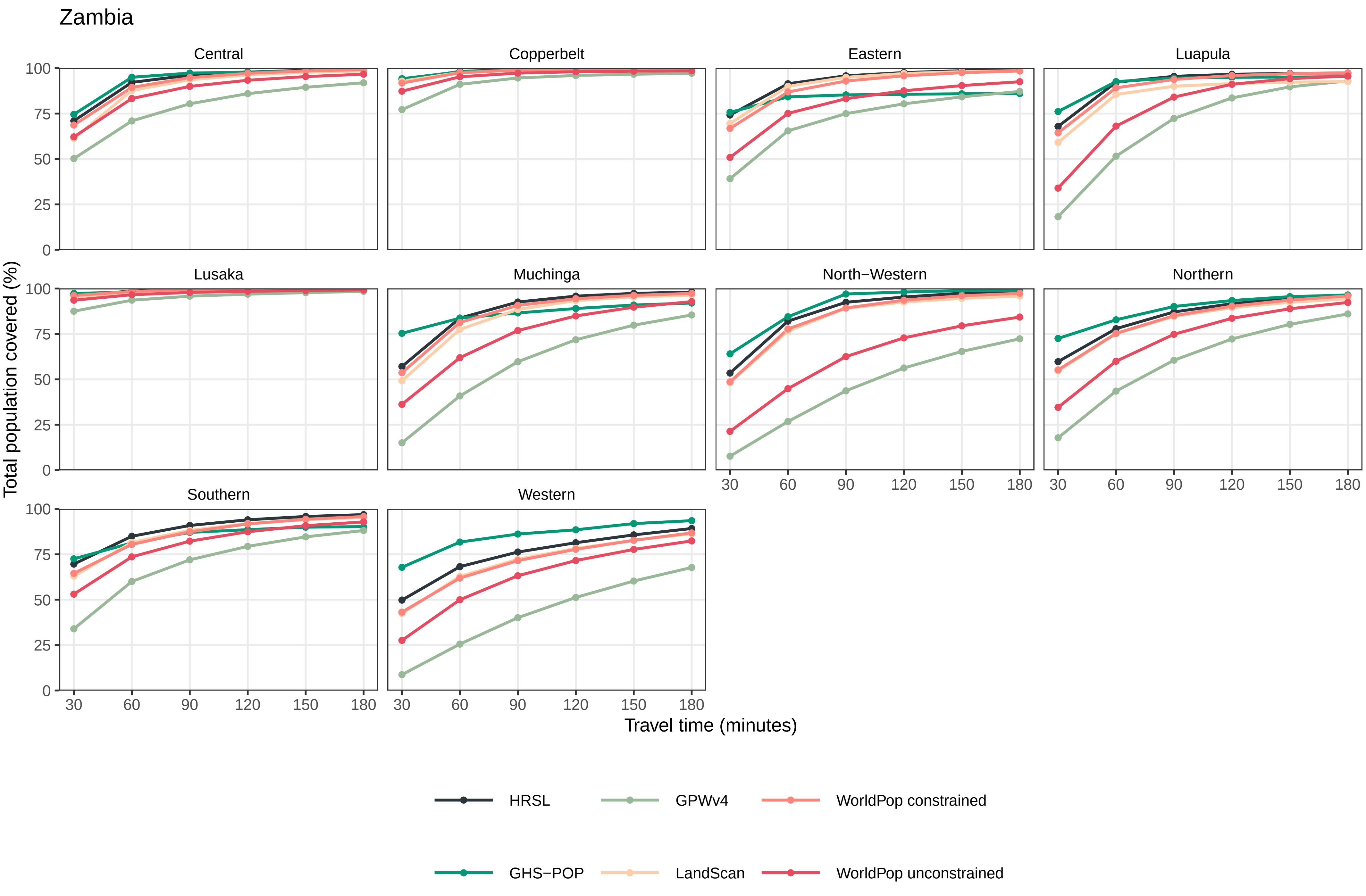

Supplementary Figure 50 − Accessibility coverage at 30, 60, 90, 120, and 150 minutes travel time at administrative level 1 for Zambia.

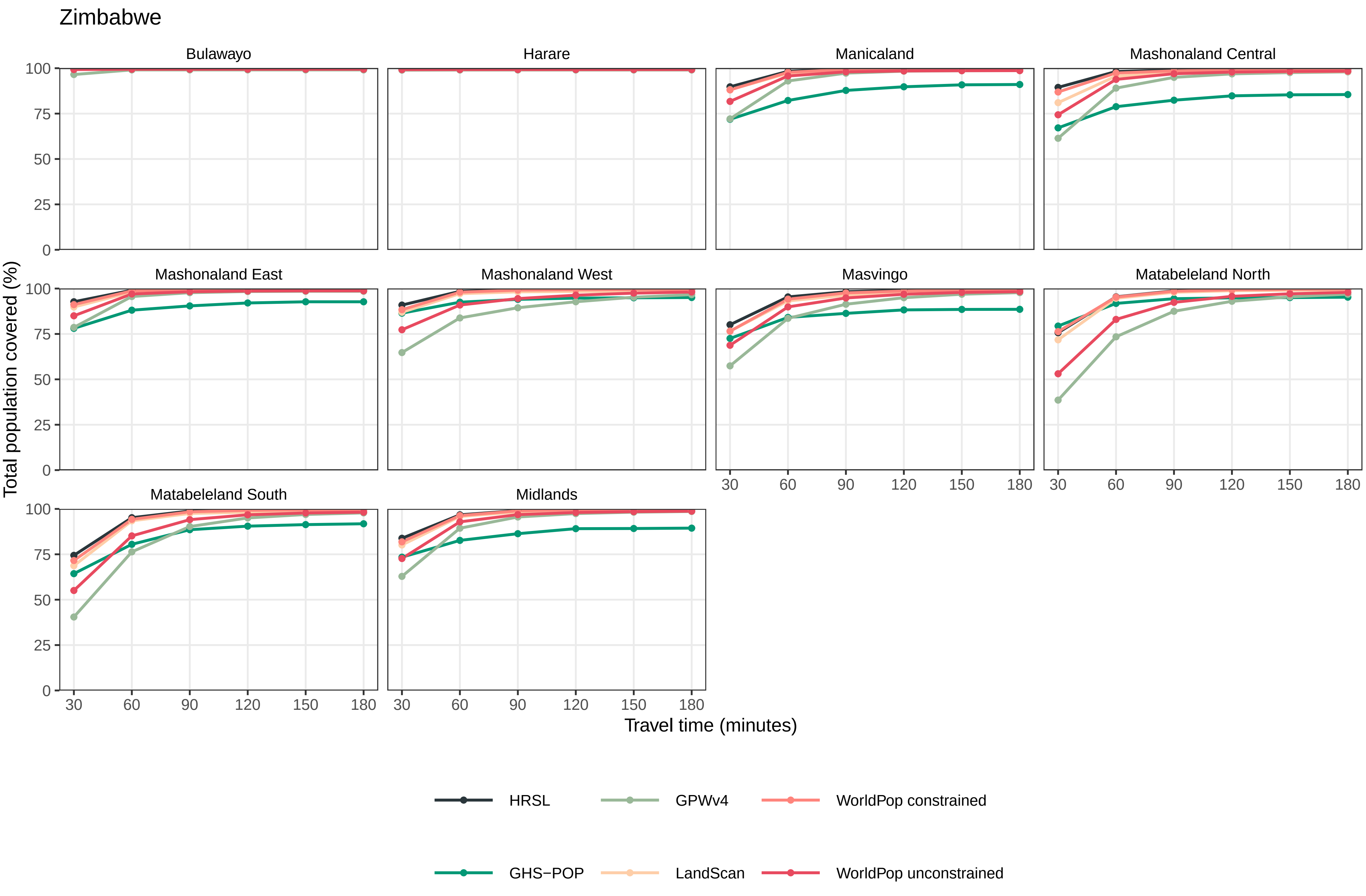

Supplementary Figure 51 − Accessibility coverage at 30, 60, 90, 120, and 150 minutes travel time at administrative level 1 for Zimbabwe.

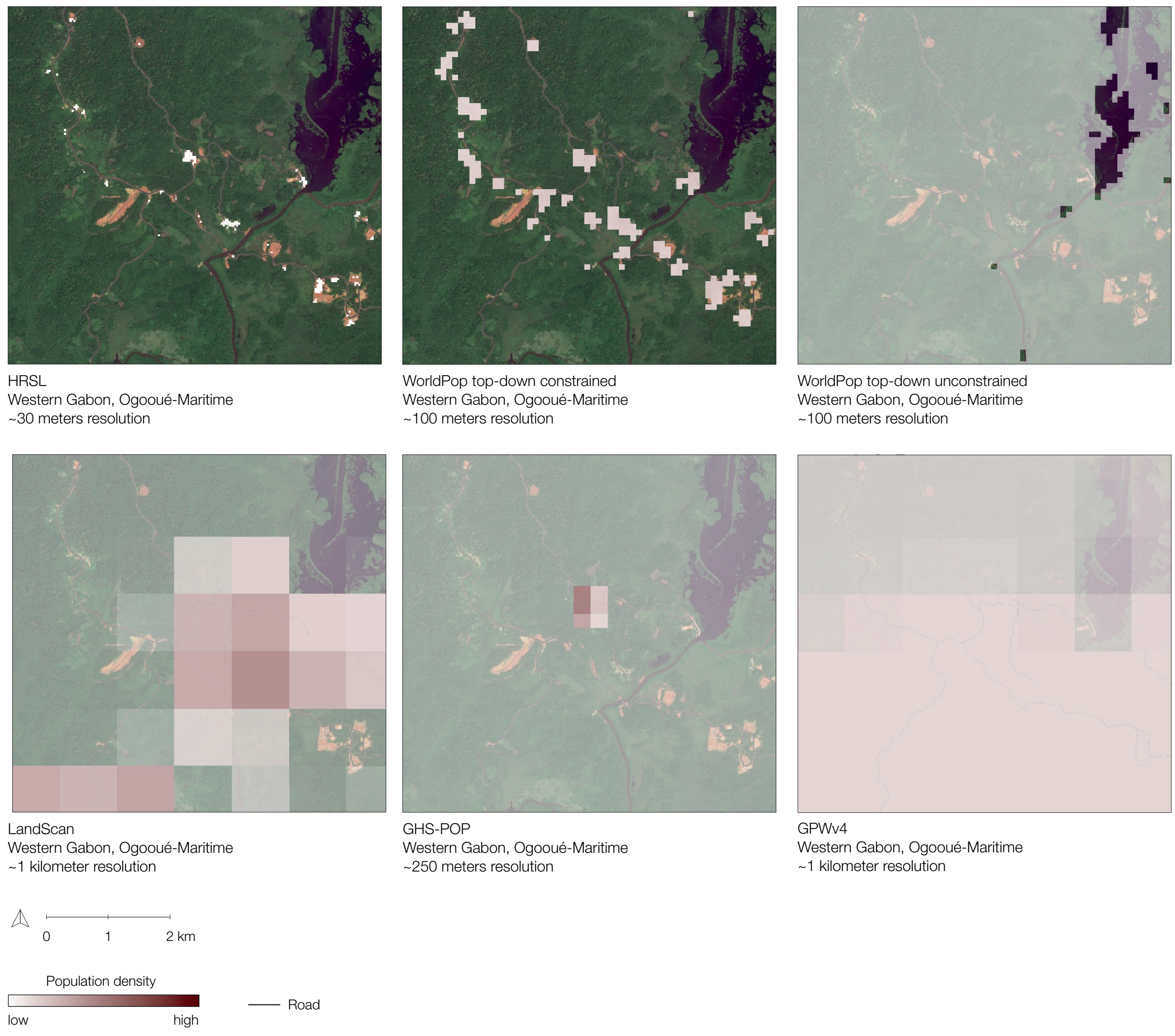

Supplementary Figure 52 – Visual differences between a) HRSL, b) WorldPop top-down constrained, c) WorldPop top-down unconstrained, d) LandScan, e) GHS-POP, and f) GPWv4 for Western Gabon, Ogooué-Maritime. Google satellite imagery as background (Map data© 2015 Google). White transparent color represents low numbers of population density.

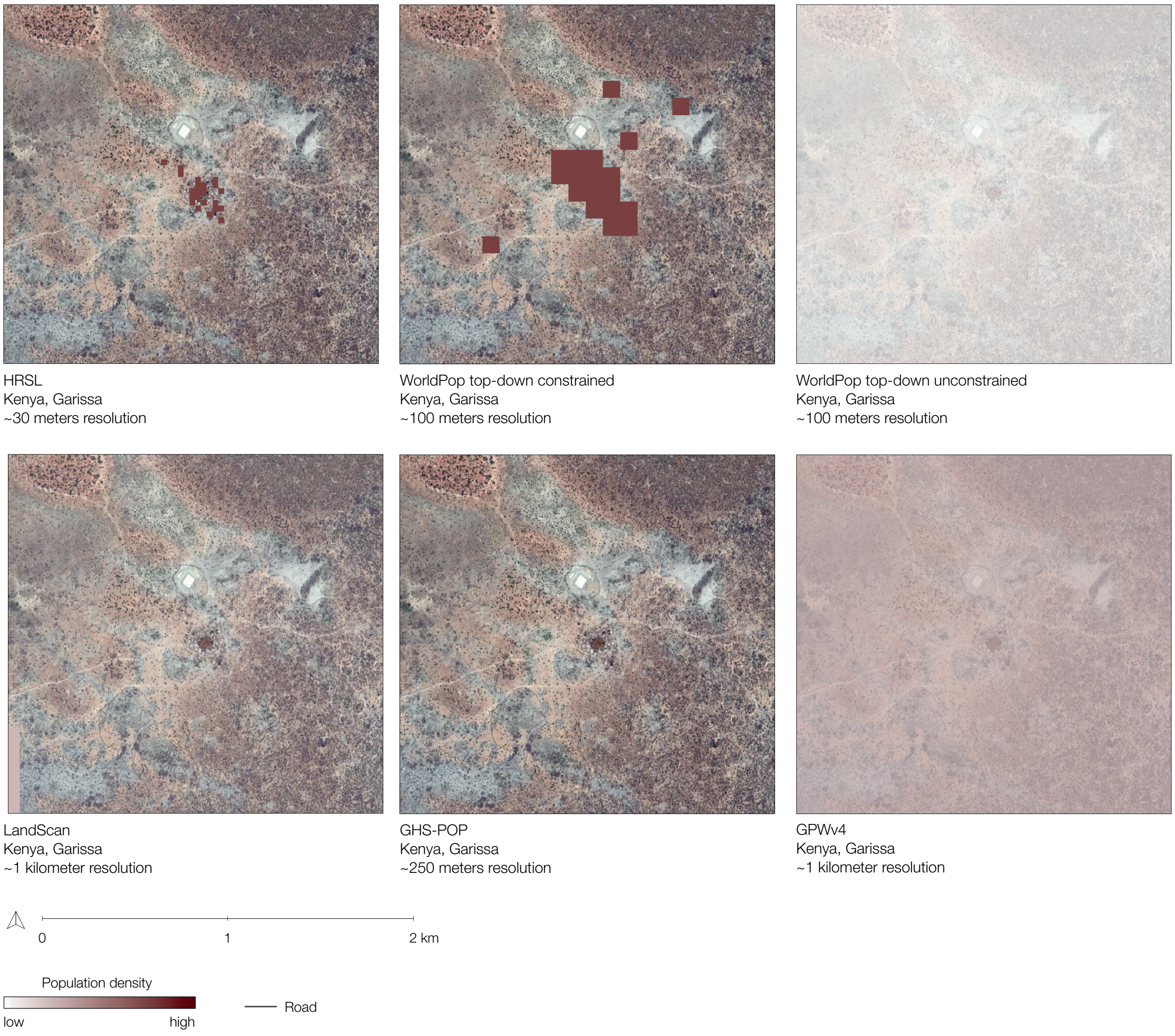

Supplementary Figure 53 – Visual differences between a) HRSL, b) WorldPop top-down constrained, c) WorldPop top-down unconstrained, d) LandScan, e) GHS-POP, and f) GPWv4 for Garissa, Kenya. Google satellite imagery as background (Map data© 2015 Google). White transparent color represents low numbers of population density.

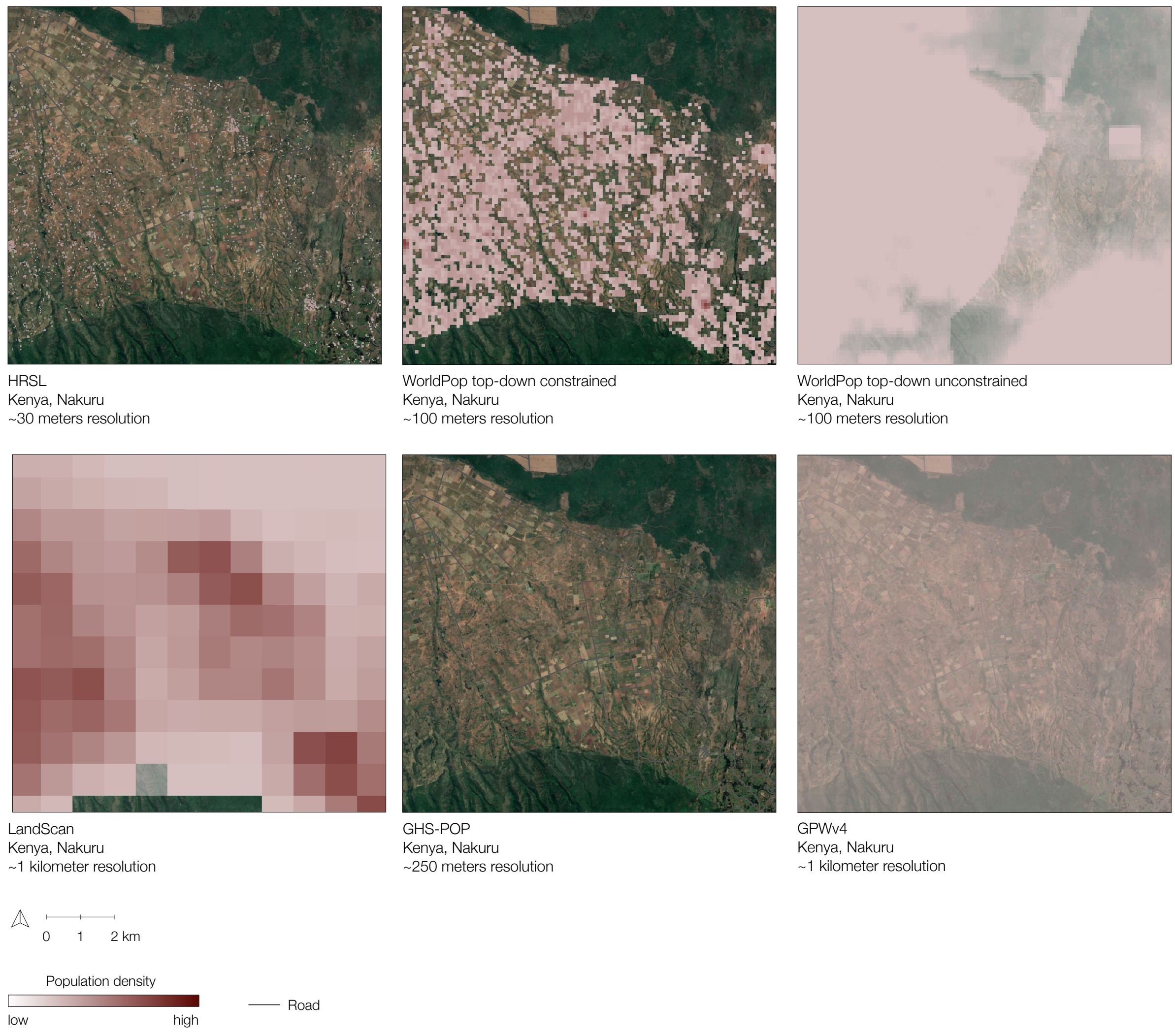

Supplementary Figure 54 – Visual differences between a) HRSL, b) WorldPop top-down constrained, c) WorldPop top-down unconstrained, d) LandScan, e) GHS-POP, and f) GPWv4 for Nakuru, Kenya. Google satellite imagery as background (Map data© 2015 Google). White transparent color represents low numbers of population density.

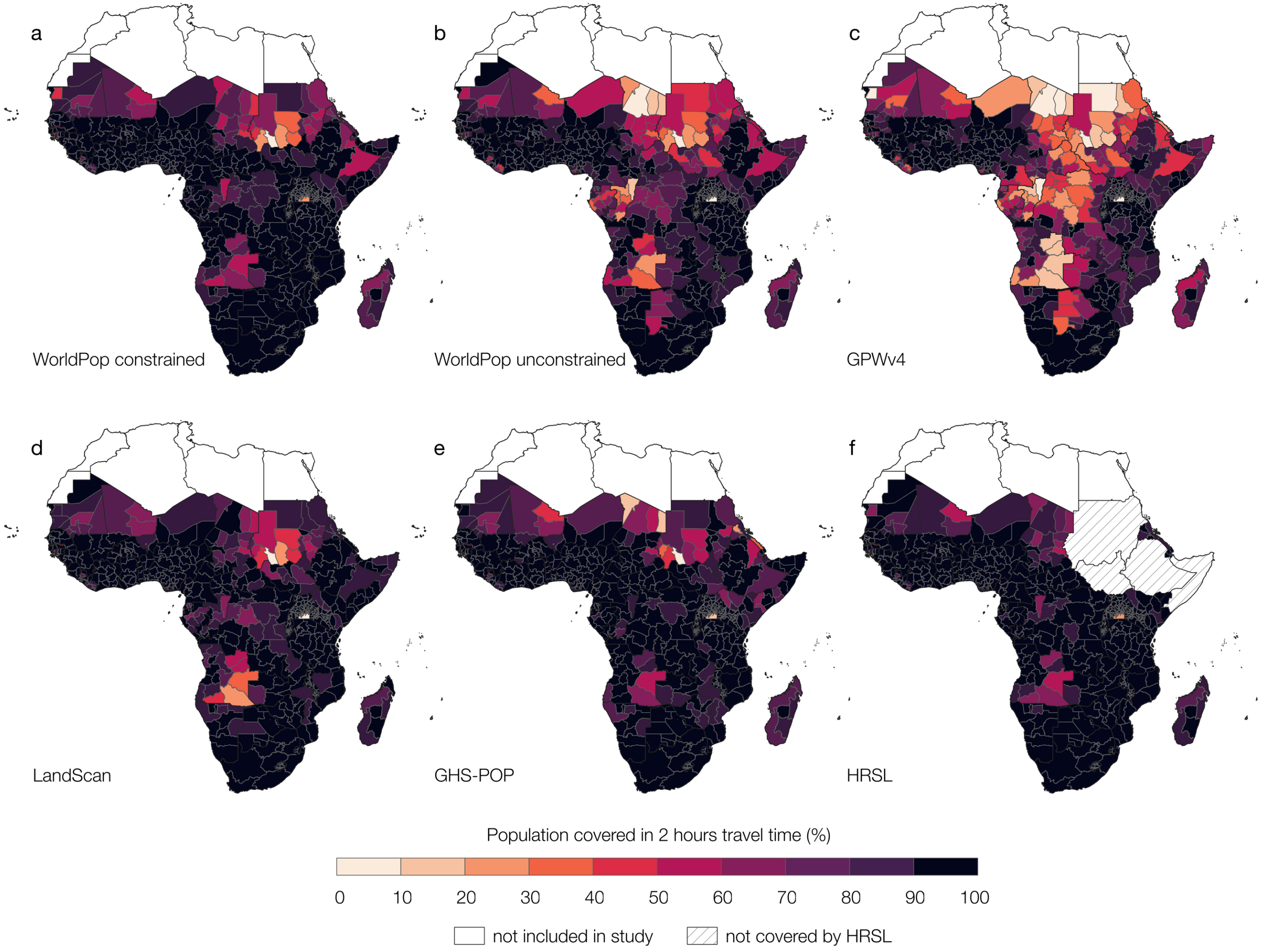

Supplementary Figure 55 – Zonal statistics of accessibility coverage at 2-hours travel time at administrative level 1 for sub-Saharan Africa. All 6 datasets can be compared.
