# Supplementary material for: Differences between gridded population data impact measures of geographic access to healthcare in sub-Saharan Africa
Source: Commun Med (Lond). 2022 Sep 16;2:117. doi: 10.1038/s43856-022-00179-4 (PMC9481590; doi:10.1038/s43856-022-00179-4)
Supplement: Supplementary file 6 — Reporting Summary [file 43856_2022_179_MOESM6_ESM.pdf]

nature research

Corresponding author(s): Fleur Hierink

Last updated by author(s): Aug 18, 2022

Reporting Summary

Nature Research wishes to improve the reproducibility of the work that we publish. This form provides structure for consistency and transparency in reporting. For further information on Nature Research policies, see our Editorial Policies and the Editorial Policy Checklist.

Statistics

For all statistical analyses, confirm that the following items are present in the figure legend, table legend, main text, or Methods section.

n/a	Confirmed	
☒	☐	The exact sample size (n) for each experimental group/condition, given as a discrete number and unit of measurement
☒	☐	A statement on whether measurements were taken from distinct samples or whether the same sample was measured repeatedly
☒	☐	The statistical test(s) used AND whether they are one- or two-sided<br>*Only common tests should be described solely by name; describe more complex techniques in the Methods section.*
☒	☐	A description of all covariates tested
☒	☐	A description of any assumptions or corrections, such as tests of normality and adjustment for multiple comparisons
☒	☐	A full description of the statistical parameters including central tendency (e.g. means) or other basic estimates (e.g. regression coefficient) AND variation (e.g. standard deviation) or associated estimates of uncertainty (e.g. confidence intervals)
☒	☐	For null hypothesis testing, the test statistic (e.g. F, t, r) with confidence intervals, effect sizes, degrees of freedom and P value noted<br>*Give P values as exact values whenever suitable.*
☒	☐	For Bayesian analysis, information on the choice of priors and Markov chain Monte Carlo settings
☒	☐	For hierarchical and complex designs, identification of the appropriate level for tests and full reporting of outcomes
☒	☐	Estimates of effect sizes (e.g. Cohen's d, Pearson's r), indicating how they were calculated

Our web collection on statistics for biologists contains articles on many of the points above.

Software and code

Policy information about availability of computer code

Data collection	Raw data that was used to derive the findings of this study are openly available, as indicated in Table 1. Respective versions and URLs linking to the gridded population data can be extracted from Supplementary Table 2 and the references. The gridded population data from LandScan are not publicly available, but can be requested from their database, however specific licensing rules may apply.
Data analysis	The R and Python code for data processing and analysis are available at Github [https://github.com/fleurhierink/Population_Access] and Zenodo [https://doi.org/10.5281/zenodo.7004009].

For manuscripts utilizing custom algorithms or software that are central to the research but not yet described in published literature, software must be made available to editors and reviewers. We strongly encourage code deposition in a community repository (e.g. GitHub). See the Nature Research guidelines for submitting code & software for further information.

Data

Policy information about availability of data

All manuscripts must include a data availability statement. This statement should provide the following information, where applicable:

- Accession codes, unique identifiers, or web links for publicly available datasets
- A list of figures that have associated raw data
- A description of any restrictions on data availability

The open and raw data that were used to derive the findings of this study are available via the relevant resources as indicated in Table 1 and Supplementary Table 2. The gridded population data from LandScan are not publicly available, but can be requested from their database, however specific licensing rules may apply. All results that were obtained from this study are accessible in Supplementary Data 2. Source data for the main figures are available in Table 2 and Supplementary Data 2.

Field-specific reporting

Please select the one below that is the best fit for your research. If you are not sure, read the appropriate sections before making your selection.

☐ Life sciences ☐ Behavioural & social sciences ☒ Ecological, evolutionary & environmental sciences

For a reference copy of the document with all sections, see nature.com/documents/nr-reporting-summary-flat.pdf

Ecological, evolutionary & environmental sciences study design

All studies must disclose on these points even when the disclosure is negative.

Study description	Geospatial analysis aimed at evaluating the impact of using different gridded population data as input to geographic accessibility models to estimate population coverage of health facilities in sub-Saharan Africa. We used six different gridded population datasets to compare differences in healthcare coverage estimates at different administrative levels, namely: sub-Saharan Africa, national, and sub-national. The study comprises a comparison of six datasets for 50 countries in sub-Saharan Africa and also introduces an automated workflow to assess accessibility at large spatial scales, for the first time at a resolution of 100 meters.
Research sample	We systematically assess differences between estimates of geographic healthcare accessibility for all of sub-Saharan Africa using the most popular gridded population data products: 1) WorldPop top-down constrained, 2) WorldPop top-down unconstrained, 3) High Resolution Settlement Layer, 4) Gridded Population of the World v4, 5) LandScan, and 6) Global Human Settlement Population
Sampling strategy	We compared estimates of healthcare coverage using six different population datasets for 50 countries in sub-Saharan Africa at 100 meters resolution. This scale seemed the best compromise between computational efficiency, spatial sufficiency to address smaller scale disparities in health care access, and in terms of granular data availability of the required spatial data described above.
Data collection	All data needed for the geographic accessibility analysis and the coverage comparison was collected from openly available data sources that can be consulted through programming libraries in R or through open databases as indicated in Table 1 and Supplementary Table 2.
Timing and spatial scale	Data were downloaded and processed between January 2021 and October 2021, however reference dates of the data can date back to 2018 for health facility coordinates and 2019 for the land cover and some of the gridded population data sets as indicated in Table 1 and Supplementary Table 2.
Data exclusions	No data was excluded from the analysis.
Reproducibility	The open and raw data that were used to derive the findings of this study are available via the relevant resources as indicated in Table 1 and Supplementary Table 2. The gridded population data from LandScan are not publicly available, but can be requested from their database, however specific licensing rules may apply. All results that were obtained from this study are accessible in Supplementary Data 2. The R and Python code for data processing and analysis are available at Github [https://github.com/fleurhierink/Population_Access] and Zenodo [https://doi.org/10.5281/zenodo.7004009].
Randomization	N/A
Blinding	N/A

Did the study involve field work? ☐ Yes ☒ No

Reporting for specific materials, systems and methods

We require information from authors about some types of materials, experimental systems and methods used in many studies. Here, indicate whether each material, system or method listed is relevant to your study. If you are not sure if a list item applies to your research, read the appropriate section before selecting a response.

Materials & experimental systems

n/a	Involved in the study	
☒	☐	Antibodies
☒	☐	Eukaryotic cell lines
☒	☐	Palaeontology and archaeology
☒	☐	Animals and other organisms
☒	☐	Human research participants
☒	☐	Clinical data
☒	☐	Dual use research of concern

Methods

n/a	Involved in the study	
☒	☐	ChIP-seq
☒	☐	Flow cytometry
☒	☐	MRI-based neuroimaging
